# Apigenin mitigates intestinal barrier dysfunction in sepsis by modulating the AKT signaling pathway

**DOI:** 10.1186/s12876-025-04196-0

**Published:** 2025-08-30

**Authors:** Zheng Lijun, Wang Liping, Zhao Dandan, Zhao Wenjie, Xu Renjie, Ji Xueyan, Che Wenqing, Li Chen, Zhao Lu

**Affiliations:** 1 Department of Pharmacy, Fujian Medical University Union Hospital, Fuzhou, 350122 China; 2 Jiangsu Key Laboratory of New Drug Research and Clinical Pharmacy, Xuzhou Medical University, Xuzhou, 221004 China; 3 Department of Gastroenterology, Xuzhou TCM Hospital, Nanjing University of Chinese Medicine, Xuzhou, 221000 China; 4 Department of Clinical pharmacy, Shaoxing Women and Children’s Hospital, Shaoxing, 312000 Zhejiang China; 5 Department of Emergency Medicine Center, The Affiliated Hospital of Xuzhou Medical University, Xuzhou, 221000 China

**Keywords:** Sepsis, Intestinal barrier, Apigenin, Caco-2, Network pharmacology

## Abstract

Sepsis is a systemic inflammatory response syndrome triggered by infection, which can lead to multiple organ dysfunction. This study untangles the synergistic multi-mechanistic effects of the natural flavonoid apigenin in ameliorating this pathological process. Utilizing a murine sepsis model and Caco-2 cell line, we systematically investigated the impact of apigenin on intestinal barrier function. Apigenin treatment (50 mg/kg) markedly improved intestinal barrier integrity, as shown by reduced serum FITC-dextran levels and restored expression of tight junction proteins Occludin, Claudin-1, and ZO-1. The compound simultaneously attenuated systemic inflammation by lowering IL-6 and TNF-α levels. Network pharmacology and molecular docking identified AKT1 and MMP-9 as key molecular targets of apigenin, which was experimentally validated through observed suppression of MMP-9 and COX-2 protein expression. These results demonstrate apigenin’s capacity to preserve intestinal barrier function during sepsis through coordinated anti-inflammatory and barrier-repair mechanisms.

**Supplementary Information:**

The online version contains supplementary material available at 10.1186/s12876-025-04196-0.

## Introduction

Sepsis represents a life-threatening condition characterized by a dysregulated host response to infection, leading to systemic inflammation and potential organ dysfunction [1]. Although research has advanced our understanding of its pathogenesis, sepsis continues to pose major clinical challenges in intensive care settings [2]. Alarmingly, epidemiological data indicate a 30% increase in sepsis incidence during the past ten years [3]. The destructive systemic inflammatory response during sepsis result in widespread organ overload and damage including acute lung injury, kidney failure, cardiac dysfunction, and other complications.

The intestine is susceptible to sepsis and plays a crucial role in the pathophysiology of sepsis. As a physical and functional barrier, the intestinal barrier prevents pathogen invasion and translocation of microorganisms and bacterial toxins into systemic circulation [4]. Accumulating evidence suggests that during sepsis, the barrier is disrupted, allowing viable bacteria and their antigens to spread to other areas, which contributes to the onset or worsening of sepsis [5]. Identifying compounds capable of preserving intestinal barrier integrity through anti-inflammatory mechanisms therefore represents a critical therapeutic strategy.

Flavonoids, abundant in plant-based foods, demonstrate diverse bioactivities relevant to intestinal health [6, 7]. Apigenin (4’,5,7-trihydroxyflavone), a flavonoid compound widely found in numerous fruits and vegetables, including celery, onion, garlic, bell pepper, guava, passionflower and bilimbi fruit, exhibits a wide range of nutritional and biological functions, such as anti-inflammatory [8], neuroprotection [9], liver protection [10, 11], and anti-tumor properties [12, 13]. In recent years, numerous studies have demonstrated that apigenin can significantly inhibit a variety of inflammatory responses induced by LPS [14, 15]. However, the protective effects of apigenin on intestinal barrier damage caused by sepsis are still unclear. This study investigates this question using LPS-challenged mice and Caco-2 cell models.

Despite the well-established anti-inflammatory properties of apigenin, the specific mechanisms underlying its intestinal barrier protection during sepsis remain largely unexplored. To address this critical knowledge gap, our study pioneers a novel investigation integrating network pharmacology, molecular docking, and experimental validation. We aim to comprehensively elucidate both the protective effects and fundamental mechanisms of apigenin against sepsis-induced intestinal barrier dysfunction, utilizing a combined approach of in silico analyses (network pharmacology and molecular docking) with established in vivo and in vitro models.

## Materials and methods

### Ethics statement

The present study was approved by The Animal Ethics Committee of Xuzhou Medical University (Xuzhou, China No. 202303T019). We minimized animal pain and discomfort throughout the experiments, which were performed at Xuzhou Medical University.

### Mice treatment and septic model building

**Fig. 1:**
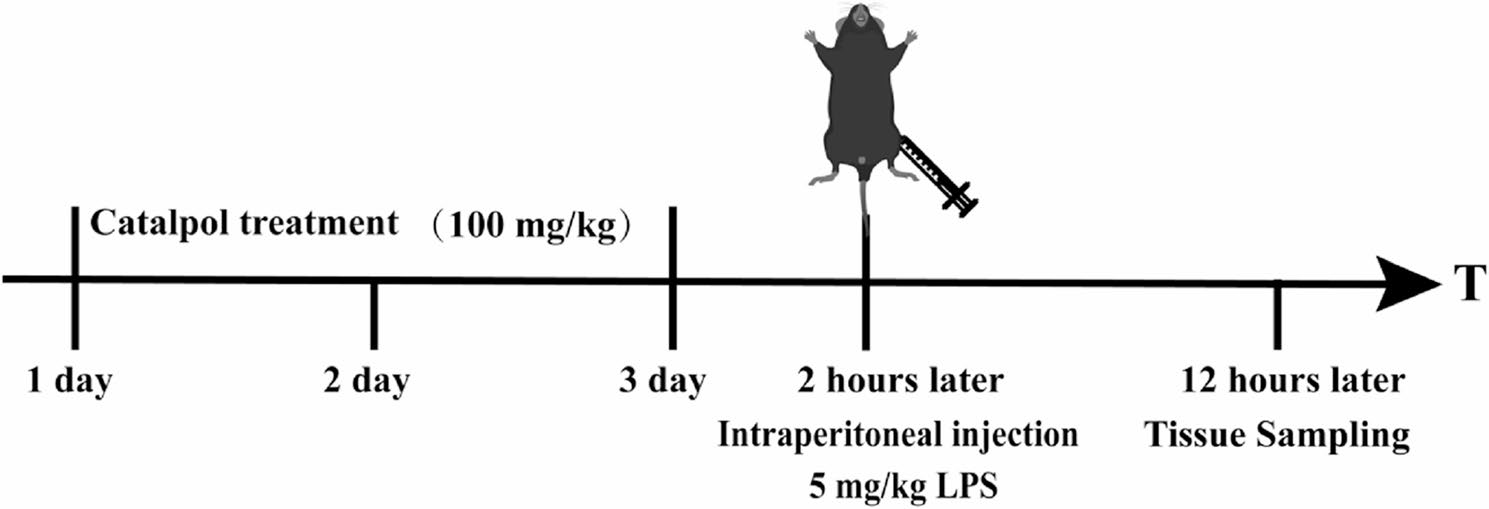
Schematic of the experimental protocol for apigenin treatment in lipopolysaccharide-induced endotoxemia

A total of 30 C57/BL6 male mice (age, 8 weeks; weight, 20 ± 2 g) were obtained from the Laboratory Animal Center of Xuzhou Medical University (Xuzhou, China). All mice were housed in a specific pathogen free laboratory animal room under controlled temperature (23 ± 1℃) and at 65–70% relative humidity. Following randomization, mice were allocated into three groups: [1] Normal control [2], LPS-induced endotoxemia model, and [3] LPS-induced endotoxemia + apigenin treatment. Investigators blinded to group assignments performed all procedures and outcome assessments. The mice of Model group and apigenin group were injected intraperitoneally with LPS at a dosage of 5 mg/kg body weight, and the mice from Normal group injected intraperitoneally with NS. The LPS-induced endotoxemia model is widely used to mimic the inflammatory response and intestinal injury characteristics of clinical sepsis. Mice in the apigenin group received daily intragastric gavage of apigenin (50 mg/kg) for 3 days prior to sepsis induction, while both the normal control and LPS-induced endotoxemia groups underwent identical volume-matched saline gavage [16]. As shown in Fig. 1, after LPS administration, the permeability of FITC-dextran was measured. At the end of the experiment, the mice were anesthetized with 3% pentobarbital sodium and euthanized by cervical dislocation following blood collection from the eyeball. Serum was separated by centrifugation at 3000 rpm for 15 min and stored at– 80 °C for subsequent ELISA analysis. Intestinal tissue samples were immediately collected, rinsed in ice-cold PBS, and either snap-frozen in liquid nitrogen or fixed in 4% paraformaldehyde for further analysis.

### ELISA

Serum was collected after different treatments of mice. Serum samples were analysed by ELISA according to the manufacturer’s protocols. IL-6, TNF-α, IL-10 and TGF-β ELISA kit (Xinle, Shanghai Xinle Biotechnology, China, cat. nos. 2209GF14ZEA, 2209GF19CER, 2307WH16GKS and 2307WH21DDE, respectively) were from Shanghai Xinle Biotechnology.

### HE staining

HE staining was performed using the HE staining kit (Solarbio, Bei Jing, China, cat. no. G1121). Formalin-fixed intestinal tissue samples of the mice from three groups were paraffin-embedded and laterally sliced to a thickness of 5 μm. After dewaxing and dehydration, the intestine sections were stained with hematoxylin and eosin. The images were captured by an inverted microscope.

### Immunohistochemistry

Immunohistochemical staining of intestinal sections followed established protocols [17]. Tissues and cells were fixed in 4% paraformaldehyde, then blocked with 5% BSA before overnight incubation at 4 °C with Occludin primary antibodies (Proteintech, Wuhan, China; 27260-1-AP). Secondary antibody incubation proceeded for 1 h at room temperature. Stained specimens were examined and imaged using a research microscope (Olympus BX43F, Japan).

### Permeability of FITC-dextran

Mice were fasted for 12 h and then administered FITC-dextran (50 mg/kg). Four hours later, peripheral blood was collected from the mice and left to stand in the dark for 30 min and then centrifuged at 1000 g at 4 °C for 10 min to isolate the serum. The fluorescence intensity of the serum was measured, and a standard curve was used to calculate the FITC-dextran concentration in the serum.

### Cell culture and treatment

Caco-2 cells (Shanghai Cell Bank) were cultured in Dulbecco’s Modified Eagle’s Medium-Hight glucose (Gibco, DMEM-H, 12800-017) supplemented with 10% fetal bovine serum (Hyclone, SV30087.02). Cells were kept at 37 °C in a humidified atmosphere of 5% CO_2_ under standard conditions. Caco-2 monolayers were plated on Transwell filters with 0.4-µm pore (Corning Incorporated, Corning, cat. no. 09822025) until they reached confluency and tight junction maturation on day 21. Culture medium was changed every 2 days. Based on our preliminary experiments and other studies on apigenin transport, it has been demonstrated that apigenin at a concentration of 90 µM does not exhibit cytotoxicity [18]. Furthermore, apigenin at 10 µM concentration has shown potent anti-inflammatory effects. Therefore, we selected a concentration of 10 µM for our study [19]. The Caco-2 cells were divided into three groups as follows: Control group, LPS group and LPS + API group. Then, of LPS group and LPS + API group were treated with LPS (10 µg/mL) for 48 h. API (10 µM) were added 30 min prior to adding LPS in LPS + API group. Furthermore, changes in the transepithelial electrical resistance (TEER) value of Caco-2 monolayers in three groups were detected within a 48-hour period.

### TEER measurements

The Caco-2 monolayer integrity was assessed through TEER measurements with a chop-stick electrode device (Millicell-ERS voltmeter, Millipore), following established methods [20]. Inserts with TEER value > 400 Ω·cm^2^ were considered valid for conducting the corresponding experiments. TEER value were examined before and after treatment of LPS (10 µg/mL).

### Prediction of the therapeutic potential and functional pathways of apigenin

We employed several databases, such as PharmMapper [21], Comparative Toxicogenomics Database (CTD) [22], Similarity Ensemble Approach (SEA) [23], and SwissTargetPrediction [24], to gather potential targets of apigenin. We obtained potential targets of sepsis from GeneCards [25] and DrugBank [26]. By comparing the lists, we identified the overlapping targets.

Subsequently, we used the STRING database [27] (https://string-db.org/) to perform an analysis of the interactions among the sepsis-related targets of apigenin. The protein-protein interaction (PPI) data was used to construct a compound-target network through the Cytoscape-v3.9.1 software [28]. Gene Ontology (GO) terms and Kyoto Encyclopedia of Genes and Genomes (KEGG) pathways with a p-value < 0.01 were considered significant. We selected the top 20 entries and visualized them in a bubble chart using the online Omishare website.

### Molecular docking

Key targets and apigenin were selected for molecular docking. The structures of apigenin were retrieved from the PubChem database [29] (https://pubchem.ncbi.nlm.nih.gov/) and converted to MOL2 format using the Open Babel GUI. AutoDockTools (version 1.5.6) facilitated molecular docking between apigenin and the proteins.

### Quantitative real-time PCR

Quantitative real-time PCR was performed as described [19]. Total RNA was extracted from tissue samples and cells using Trizol reagent (cat#: 9109; Invitrogen, Carlsbad, CA, USA) and was reverse transcribed into cDNA using the PrimeScript™ RT Reagent Kit (cat#: RR037A; TAKARA BIO INC, Shiga, Japan) following the manufacturer instructions. The primer sequences used for qPCR are listed in Table 1. The conditions of amplification reaction were 95 ℃ for 10 min, followed by 40 cycles of 95℃ for 15 s and 60 ℃ for 1 min. The relative graphs and statistical analyses of transcript quantities were calculated using the 2^−∆∆Ct^ method with GAPDH as the endogenous reference gene amplified from the samples. The qPCR analysis was carried out 3 times.

**Table 1:** Primer sequences of qRT-PCR primer of mouse

Primer	Sequence(5’ to 3’)
GAPDH	F: AGAAGGTGGTGAAGCAGGCATC
	R: CGAAGGTGGAAGAGTGGGAGTTG
ZO-1	F: TGCTAATGCCTCGGAAAGAGATGAC
	R: GCTGTGGAGACTGCGTGGAATG
Claudin 1	F: GCTGGGTTTCATCCTGGCTTCTC
	R: CCTGAGCGGTCACGATGTTGTC
Occludin	F: TTGGGACAGAGGCTATGGGACAG
	R: ACTAAGGAAGCGATGAAGCAGAAGG
TNF-α	F: CGCTCTTCTGTCTACTGAACTTCGG
	R: GTGGTTTGTGAGTGTGAGGGTCTG
COX-2	F: CTGGTGCCTGGTCTGATGATGTATG
	R: GGATGCTCCTGCTTGAGTATGTCG
IL-6	F: GAAACCGCTATGAAGTTCCTCTCTG
	R: GTATCCTCTGTGAAGTCTCCTCTCC
IL-1β	F: CACCTCACAAGCAGAGCACAAG
	R: GCATTAGAAACAGTCCAGCCCATAC

### Western blot analysis

Western blot analysis was performed using standard procedures. In brief, and tissues were separately lysed using RIPA buffer with 1% phenylmethanesulfonyl fluoride (PMSF) and protease inhibitor cocktail (Roche Diagnostics, Basel, Switzerland). Then, the protein concentration of cell lysates was quantified using the BCA method (Beyotime, Shanghai, China). Subsequently, 20 µg amounts of protein were transferred to polyvinylidene fluoride (PVDF) membranes following separation by 12.5% SDS-PAGE. The membranes were then blocked by 5% bovine serum album in Tris-buffered saline for 1 h at room temperature, followed by incubation with primary antibodies at 4 °C overnight against A. GAPDH (Bioworld, Nan Jing, China, cat. no. AP0063). COX-2(Zenbio, R23971). IL-1β, IL-6, Occludin, Claudin, ZO-1, p-AKT, AKT and MMP-9 antibody were purchased from Proteintech. (cat. no. 26048-1-AP, 66146-1-Ig, 27260-1-AP, 13050-1-AP, 21773-1-AP, 28731-1-AP, 10176-2-AP and 45634-2-AP respectively, Wu Han, China). The excess primary antibody was removed by washing with Tris-buffered Saline and 0.1% Tween-20 (TBST) three times for 5 min, and the membranes were subsequently incubated with secondary antibodies (Bioworld, Nan Jing, China, cat. no. BS13278) at a dilution of 1:5,000 at room temperature for 1 h. After rinsing with TBST three times for 5 min. To ensure accurate protein loading, GAPDH (glyceraldehyde-3-phosphate dehydrogenase) was used as a loading control. Protein levels were quantified using ImageJ software, with the intensity of the target protein bands normalized to GAPDH levels for each sample. All results are presented as relative expression levels.

### Statistical analyses

All data are presented as mean ± SD. Differences between groups in mean values with normal distribution were compared by one-way ANOVA followed by Tukey test. *P* < 0.05 was considered statistically significant.

## Results

### Apigenin treatment alleviates mucosal barrier injury in septic mice

**Fig. 2:**
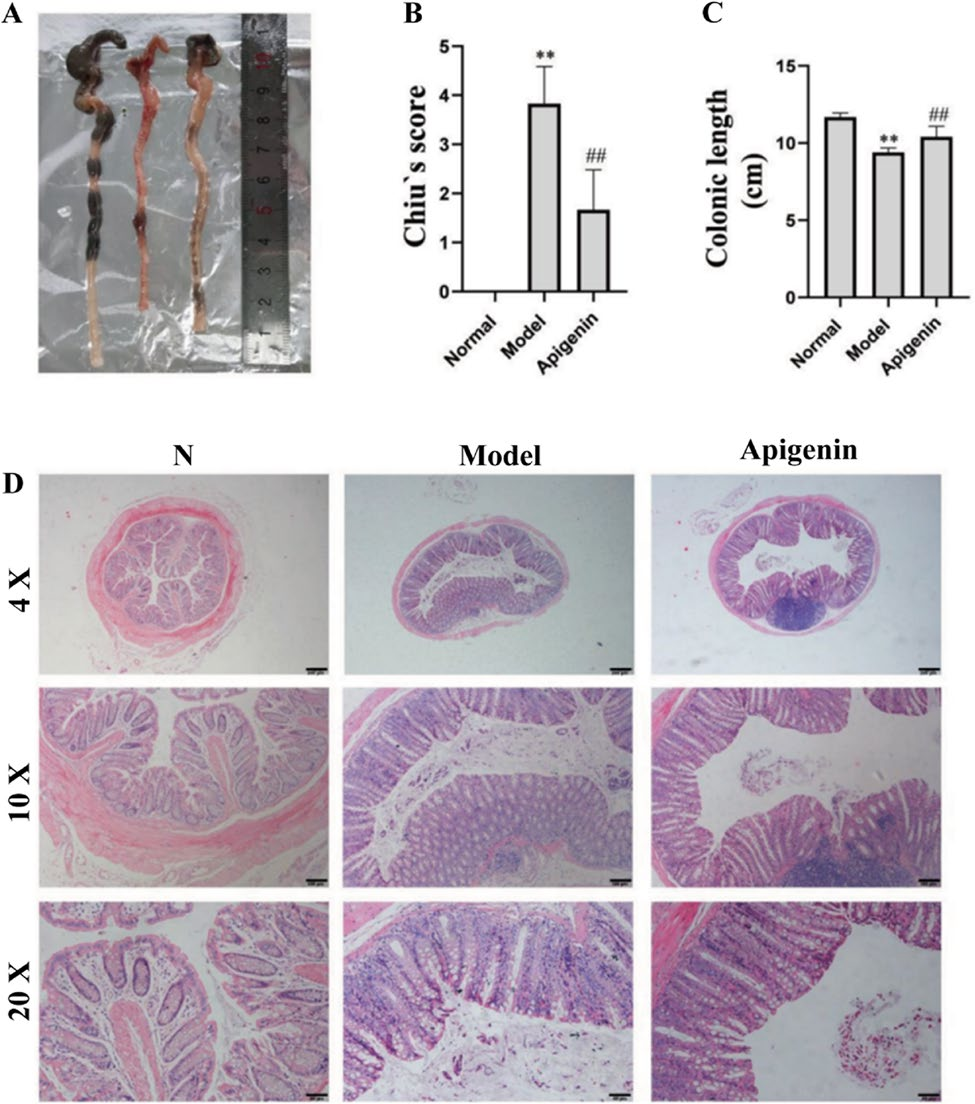
The effect of apigenin on morphology of the intestine of sepsis mice. **A** Representative images of mouse colon. **B** Histopathological assessment by Chiu’s score (0–5 scale). **C** Colonic length (cm) in each group. **D** Representative images of H&E detection of intestinal tissue of sepsis mice (scale bars = 50 μm, 100 μm, 200 μm). Data are mean ± SD (*n* = 3). ^**^P < 0.01 versus the Normal group; ^##^*P* < 0.01 versus the Model group

LPS-treated mice constitute a classic model for studying the protective effects of compounds on sepsis-induced intestinal injury. Intestinal pathological changes were evaluated using the standardized Chiu’s scoring system (0 = normal, 5 = severe injury). Two independent investigators blinded to group allocation performed histological assessments, with the final score representing the average of both evaluations. Discrepancies exceeding predetermined thresholds were resolved through consensus discussion. As shown in Fig. 2A and C, the colon length of model group mice significantly shortened compared to the control group. Treatment with apigenin (100 mg/kg) significantly reversed this shortening of colon length. Furthermore, the results of HE staining and Chiu’s score demonstrate that apigenin effectively protects the intestinal barrier.

### Effects of apigenin on serum inflammatory factors

**Fig. 3:**
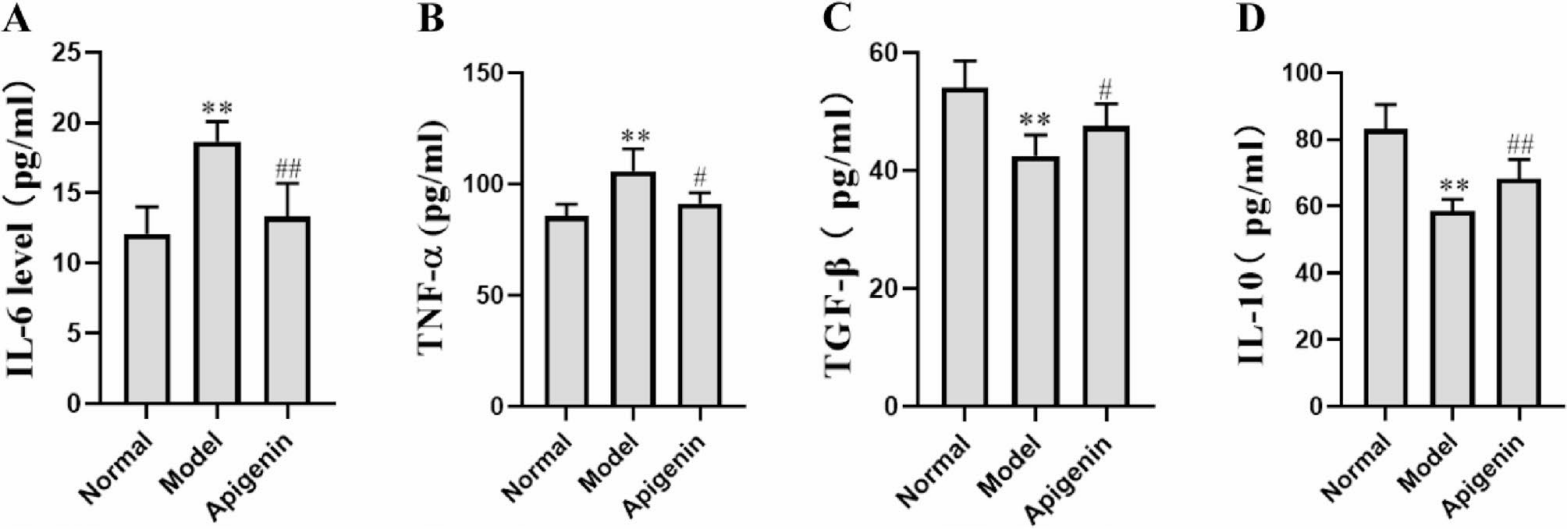
Effects of apigenin on serum inflammatory cytokines at 12 h post-LPS. **A** IL-6, **B** TNF-α, **C** TGF-β, **D** IL-10. Data are mean ± SD (*n* = 3). **P < 0.01 versus the Normal group; #*P <* 0.05, ##*P <* 0.01 versus the Model group

Severe inflammation was observed in peripheral blood and intestinal tissue 12 h after LPS administration in in the model group. As shown in Fig. 3A and B, the serum levels of IL-6 and TNF-α significantly increased in Model group. Apigenin can significantly reduce the levels of inflammatory factors IL-6 and TNF-α. The serum levels of TGF-β (Fig. 3C) and IL-10 (Fig. 3D) significantly reduced by LPS, and significantly increased in apigenin group. The pro-inflammatory cytokines, TNF-α and IL-6, play a crucial role in the initial phase of sepsis. IL-10 has been found to play a crucial role in reducing excessive inflammation during sepsis. Studies in mice have revealed that lack of IL-10 leads to multiple organ failure and increases mortality rates [30]. Apigenin can significantly regulate the levels of pro-inflammatory factors and anti-inflammatory factors, indicating that apigenin can beneficially modulate the inflammatory response in sepsis.

**Fig. 4:**
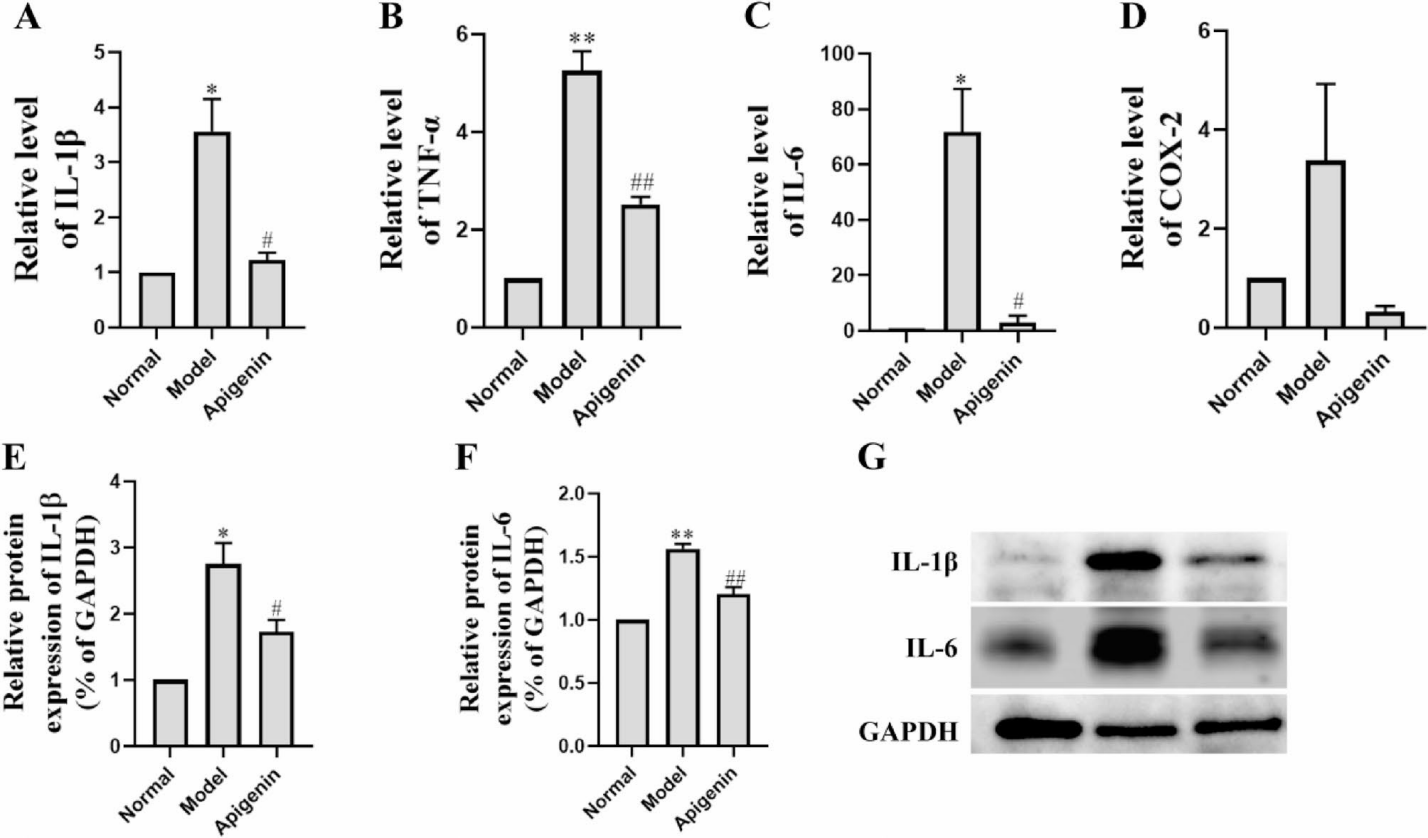
Anti-inflammatory effect of apigenin on sepsis mice. **A**–**D** The level of relative mRNA expression of IL-1β, TNF-α, IL-6, COX-2. Western blot analysis of IL-1β and IL-6 expression in intestinal tissues. **E**, **F** Densitometric analysis of IL-1β and IL-6 protein levels. Data are normalized to GAPDH. **G** Representative Western blot bands showing IL-1β and IL-6 protein expression. Data are mean ± SD (*n* = 3). ^*^P < 0.05, ^**^P < 0.01 versus the Normal group; ^#^*P* < 0.05, ^##^*P* < 0.01 versus the Model group

The results of real-time quantitative PCR confirmed the experimental findings on the effects of apigenin on the serum of mice with sepsis. Compared with the control group, the levels of IL-1β (Fig. 4A), TNF-α (Fig. 4B), and IL-6 (Fig. 4C) in intestinal tissues were significantly elevated in the model group. Additionally, COX-2 expression (Fig. 4D) exhibited an upward trend in the model group. Additionally, apigenin administration attenuated the levels of IL-1β (*P*<0.05), TNF-α (*P*<0.01) and IL-6 (*P*<0.05), Although apigenin exhibited a downward trend in reducing COX-2 mRNA expression, the effect did not reach statistical significance. In addition, the results showed that the protein level of inflammatory factors IL-1β and IL-6 were significantly increased in the intestinal tissue of the mice of model group. Apigenin administration resulted in a significantly decreased protein level of IL-1β (Fig. 4E) and IL-6 (Fig. 4F) compared with normal saline administration. These results demonstrated that apigenin treatment could decrease inflammatory factors and inflammation in peripheral blood and intestinal tissue mice induced by LPS.

### Protective effect of apigenin on tight junction proteins of intestinal tissue of sepsis mice

**Fig. 5:**
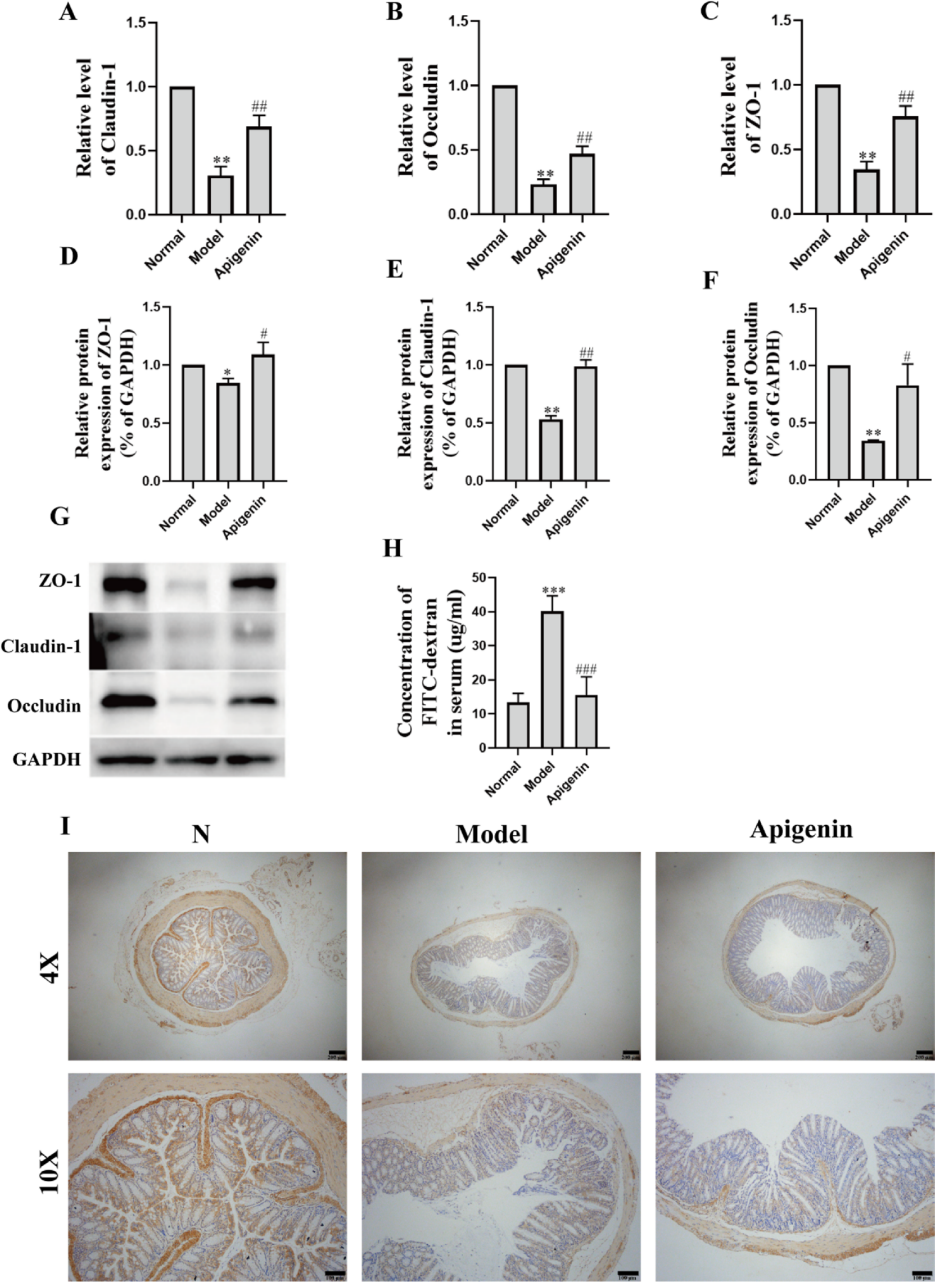
Protective effect of apigenin on tight junction proteins of intestinal tissue of sepsis mice. **A**–**C** The levels of Claudin-1, Occludin, and ZO-1 relative mRNA expression. **D**–**F** The expression of tight junction proteins. **G** The ZO-1, Claudin-1 and Occludin protein expressions in intestinal tissue of sepsis mice were determined via western blotting. **H** Quantitative analysis of FITC-dextran fluorescence intensity in serum from sepsis mice. **I** Representative images of IHC detection of ZO-1 protein in intestinal tissue of sepsis mice (scale bars = 100 μm, 200 μm). Data are mean ± SD (*n* = 3). ^*^P < 0.05, ^**^P < 0.01, ^***^P < 0.001 versus the Normal group; ^#^*P* < 0.05, ^##^*P* < 0.01, ^###^*P* < 0.01 versus the Model group

LPS significantly decreased Occludin, Claudin-1, and ZO-1 mRNA levels, which apigenin treatment effectively restored. Western blot analysis revealed corresponding reductions in these tight junction proteins within intestinal tissues, with apigenin again counteracting these effects. Immunohistochemical staining of ZO-1 (Fig. 5I) corroborated the western blot findings. These data suggest that apigenin mitigates LPS-induced intestinal mucosal injury through tight junction protein preservation. These prove that apigenin significantly attenuated LPS-induced intestinal mucosal injury in mice by increasing the level of tight junction proteins. As shown in Fig. 5H, Key functional experiments demonstrated that LPS induced intestinal barrier dysfunction by increasing serum FITC-dextran concentration, whereas apigenin treatment significantly suppressed this leakage phenomenon. Collectively, these results indicate that apigenin ameliorates LPS-induced intestinal mucosal damage by upregulating tight junction protein expression and maintaining intestinal barrier integrity.

### Apigenin prevents intestinal barrier disruption in Caco-2 cell monolayers

**Fig. 6:**
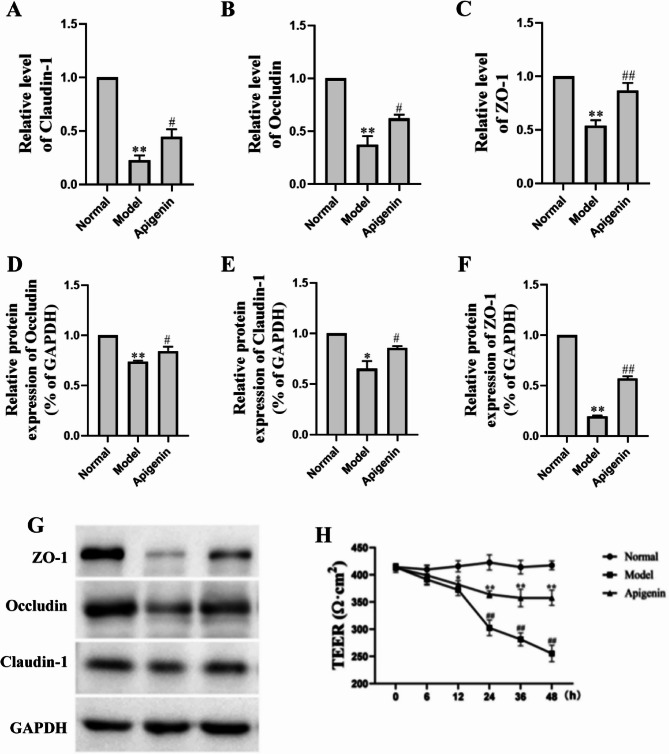
Protective effect of apigenin on tight junction proteins on Caco-2 cells. **A**–**C** Relative mRNA expression of tight junction proteins (Claudin-1, Occludin, ZO-1) in Caco-2 cells. **D**–**F** Quantification of tight junction protein levels (Western blot). **G** Representative Western blots of ZO-1, Occludin, Claudin-1. Data are mean ± SD (*n* = 3) **H** TEER values of Caco-2 monolayers under different treatments. Data are mean ± SD (*n* = 6). ^*^P < 0.05, ^**^P < 0.01 versus the Normal group; ^#^*P* < 0.05, ^##^*P* < 0.01 versus the model group

To further demonstrate the effects of apigenin on tight junction proteins, we employed Caco-2 cells using the methods described in the study [31]. In alignment with animal results, treatment with LPS significantly decreased the level of Occludin, Claudin-1 and ZO-1 protein levels. As is shown in Fig. 6, administration of the apigenin significantly reversed the decreased Occludin, Claudin-1 and ZO-1 levels. Together, these data demonstrated that apigenin could protect the intestinal epithelial barrier induced LPS. The TEER assays were performed to investigate whether apigenin had protective effects against LPS-induced integrity disruption of Caco-2 cell monolayers. As shown in Fig. 6H, LPS exposure caused a significant decrease in TEER, suggesting that the treatment of Caco-2 cell monolayers with LPS led to disruption of intestinal barrier function and loss of barrier integrity. The experiment was repeated 6 times, followed by statistical analysis. Notably, the TEER value was significantly elevated after apigenin treatment.

### PPI network of therapeutic targets for apigenin against sepsis

**Fig. 7:**
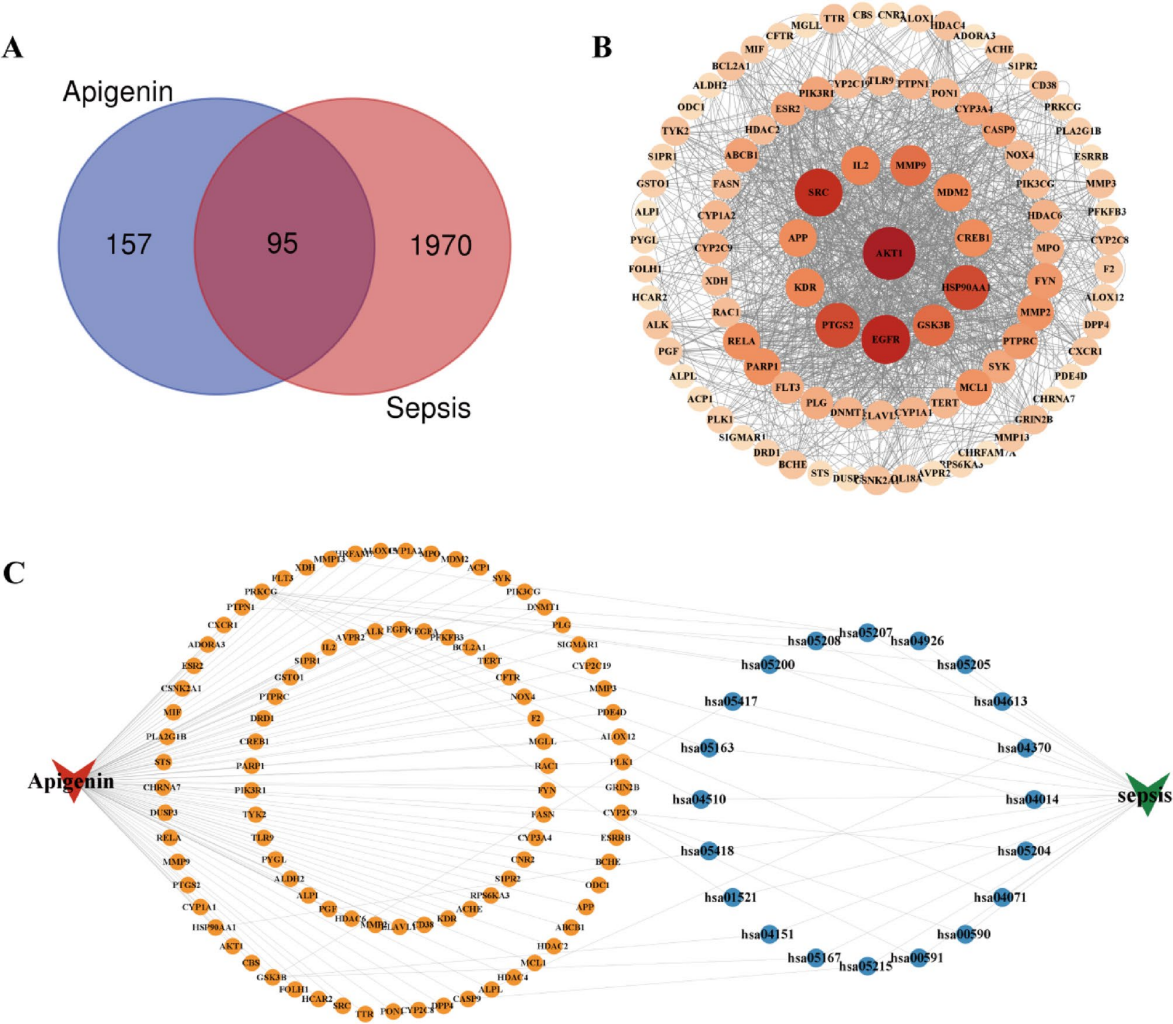
PPI Network of Therapeutic Targets for apigenin and sepsis. **A** Apigenin therapeutic targets and targets associated with sepsis. **B** PPI network map of potential targets of apigenin in the treatment of sepsis (by Degree). **C** “Component-target-pathway-disease” network diagram

To systematically unravel the molecular mechanisms of apigenin, we conducted an integrated network pharmacology analysis. A Venn diagram was generated using the Venny online tool (http://bioinformatics.psb.ugent.be/webtools/Venn/) to visualize the overlap, revealing 95 target genes shared between apigenin and sepsis (Fig. 7A). The intersected target genes were imported into the String data platform for PPI network analysis and visualized using Cytoscape-v3.9.1 (Fig. 7B). This network comprises 95 nodes and 626 edges. Notably, In addition to AKT1, network analysis suggested that hub proteins such as EGFR (growth factor signaling pathway) and GSK3B (inflammation/metabolism regulation) might be involved in the pathological processes of the intestinal barrier. Although the experimental focus was on the AKT pathway and its downstream effector molecules, the protective effect of apigenin may be multi-targeted. Future studies could further explore these potential targets.

We constructed a “compound-target-disease-pathway” network by integrating apigenin’s potential targets with sepsis-related pathways and genes using Cytoscape 3.9.1 (117 nodes and 135 edges; Fig. 7C). In the network diagram, the red inverted triangles represent apigenin, green inverted triangles indicate sepsis, orange circles denote genes, and blue circles signify pathways.

### GO functional annotation analysis and KEGG pathway analysis

**Fig. 8:**
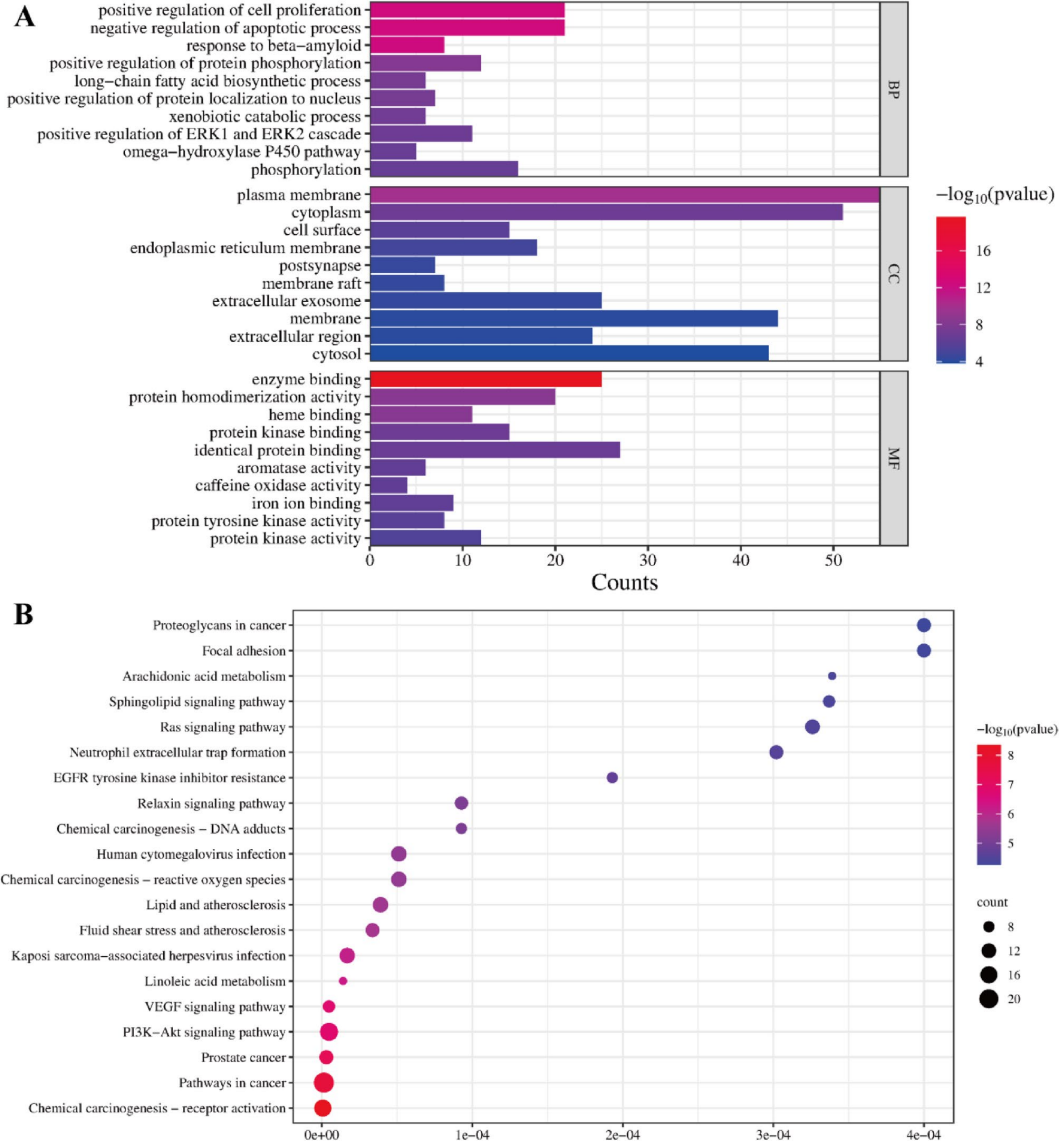
GO functional annotation analysis and KEGG pathway analysis. **A** GO functional enrichment analysis bubble map of apigenin in the treatment of sepsis. **B** KEGG functional enrichment analysis bubble map of apigenin in the treatment of sepsis

GO analysis revealed biological processes (BP), cellular components (CC), and molecular functions (MF) associated with the targets. A total of 521 GO terms were significantly enriched, comprising 351 BP, 66 CC, and 104 MF terms. The top 10 categories for BP, CC, and MF are illustrated in Fig. 8A. The top enriched pathways (by gene count and significance) included PI3K-AKT signaling, VEGF signaling, Relaxin signaling, Ras signaling, Sphingolipid signaling, as well as broader categories like “Pathways in cancer”. GO and KEGG enrichment analyses provide compelling evidence for AKT1’s central role in sepsis-associated intestinal barrier dysfunction through multiple interconnected mechanisms. The significant enrichment of the PI3K-Akt signaling pathway in KEGG analysis underscores AKT1’s regulatory importance, while molecular function GO terms (“positive regulation of protein phosphorylation” and “protein kinase activity”) directly align with AKT1’s serine/threonine kinase function and our observed phosphorylation abnormalities. Key biological processes including “positive regulation of ERK1/ERK2 cascade” and “negative regulation of apoptosis” - though not explicitly inflammatory - demonstrate established connections to AKT1/NF-κB-mediated cytokine release and cell survival [32, 33]. Furthermore, the enrichment of cellular component terms (“plasma membrane” and “cell surface”) implicates AKT1 in membrane-associated signaling relevant to barrier integrity. Collectively, these findings reveal AKT1’s multifaceted involvement in sepsis-induced intestinal injury through: [1] dysregulated phosphorylation cascades [2], NF-κB-driven inflammation [3], anti-apoptotic pathways, and [4] membrane signaling modulation, providing a robust mechanistic framework for further investigation.

### Validation of compound-target interaction by molecular docking

**Fig. 9:**
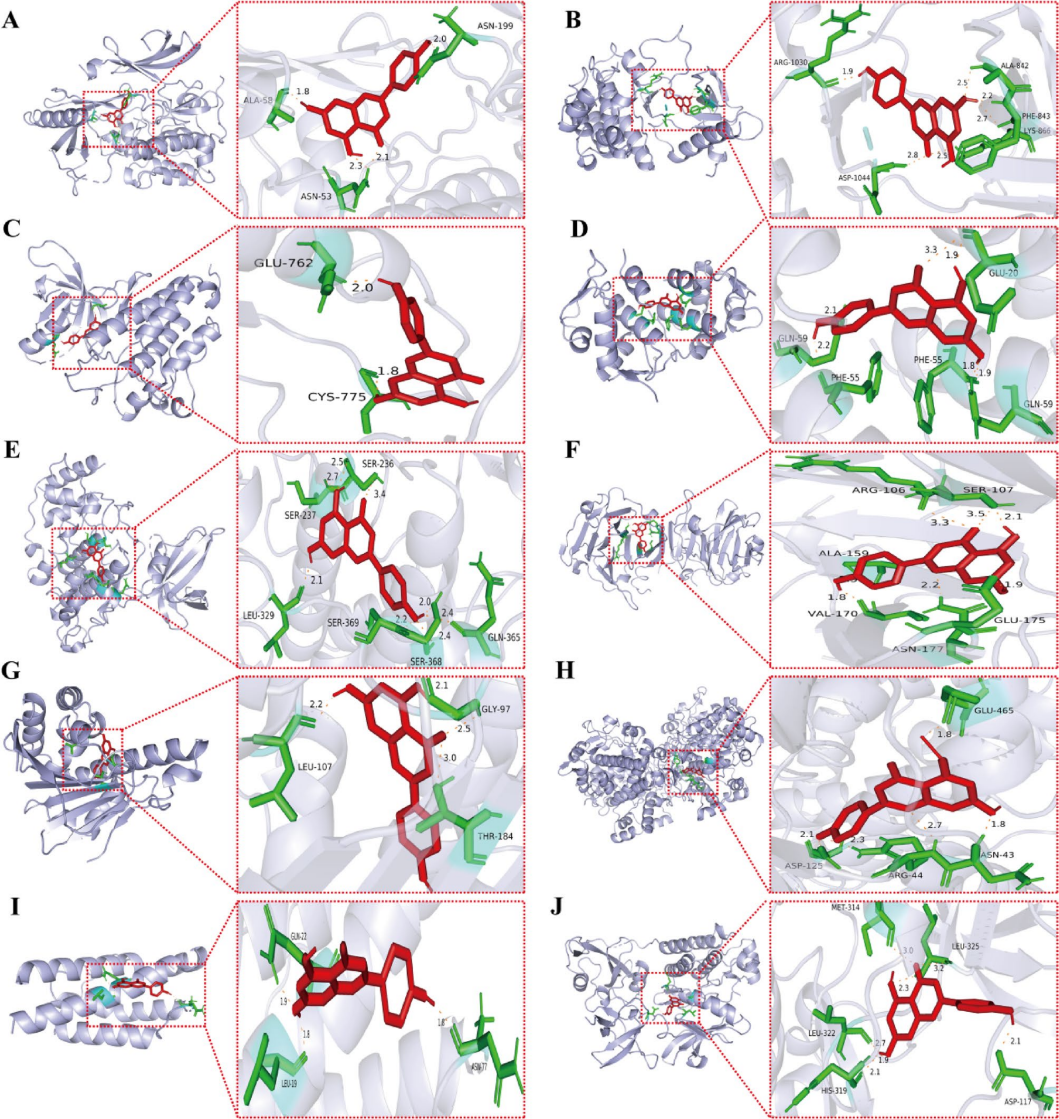
Optimal binding conformation map of key target gene docking with apigenin. Apigenin is displayed as red stick models bound to the protein binding sites (shown as ribbon diagrams). **A** AKT1 and apigenin. **B** Vascular endothelial growth factor receptor 2 (KDR = VEGFR2) and apigenin. **C** Epidermal growth factor receptor (EGFR) and apigenin. **D** E3 ubiquitin-protein ligase Mdm2 (MDM2) and apigenin. **E** Glycogen synthase kinase-3 beta (GSK3B) and apigenin. **F** MMP-9 and apigenin. **G** Heat shock protein HSP 90-alpha (HSP90AA1) and apigenin. **H** PTGS2 (COX-2) and apigenin. **I** IL-2 and apigenin. **J** (Proto-oncogene tyrosine-protein kinase Src) SRC and apigenin

This study conducted systematic molecular docking analyses on the top 10 key target proteins ranked by degree values, including AKT1, EGFR, SRC, GSK3B, HSP90AA1, IL-2, MMP-9, MDM2, PTGS2, and KDR. According to the evaluation criteria of molecular docking energetics, a binding energy of less than − 5 kcal/mol indicates a favorable binding tendency between the small molecule and the protein, while a binding energy of <– 7 kcal/mol suggests a strong interaction. The results demonstrate that apigenin exhibits high-affinity binding to AKT1 (PDB: 3O96; binding energy:– 7.36 kcal/mol), competitively occupying its ATP-binding pocket through hydrogen bonds with residues such as ALA-58, ASN-53, and ASN-199 (Fig. 9A). This binding mode spatially hinders ATP from accessing the activation loop of AKT1, potentially inhibiting its phosphorylation. The binding energy parameters of apigenin with COX-2 (– 7.03 kcal/mol) and MMP-9 (– 6.35 kcal/mol) were also highly favorable, comparable to those of reported inhibitors for these targets. Although the high binding energies suggest the possibility of direct interactions, further experimental validation (e.g., binding assays) is warranted. Notably, despite IL-2 being a typical cytokine lacking a classical small-molecule binding pocket, exploratory docking analysis was performed due to its hub position in the protein-protein interaction network (degree = 64). The results showed that the binding energy between apigenin and IL-2 was − 5.22 kcal/mol, significantly weaker than that with other targets, which aligns with the functional characteristics of cytokines that typically mediate signaling via protein-protein interactions. The optimal docking conformations for each target protein were selected for visualization (Fig. 9), with detailed docking parameters provided in Table 2.

**Table 2:** Molecular Docking binding energy of apigenin with target proteins

Gene Name	PDB ID	Binding Energy(kcal/mol)
AKT1	3O96	– 7.36
EGFR	5UG9	– 7.32
SRC	1FMK	– 7.39
GSK3B	7SXJ	– 6.46
HSP90AA1	1UY1	– 6.35
IL-2	1M47	– 5.22
MMP-9	1ITV	– 6.35
MDM2	3GO3	– 7.27
PTGS2	5IKR	– 7.03
KDR	1YWN	– 6.23

### In vitro experimental validation

**Fig. 10:**
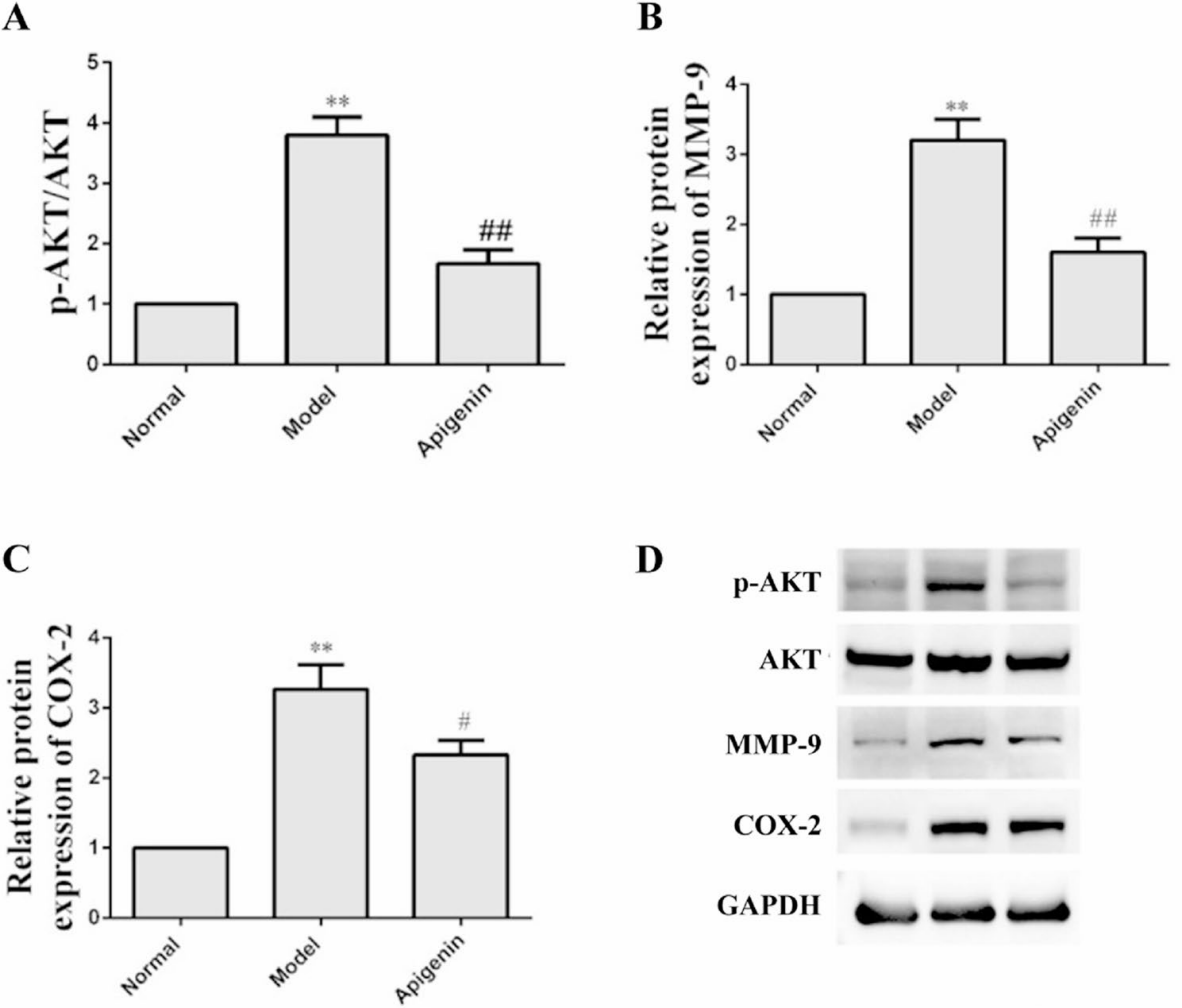
Effects of apigenin on AKT, MMP-9, and COX-2 in LPS-stimulated Caco-2 cells. **A** The protein expressions of p-AKT/AKT. **B** The protein expressions of MMP-9. **C** The protein expressions of COX-2. Data are mean ± S.D (*n* = 3). ^**^P < 0.01 versus the Normal group; ^#^*P* < 0.05, ^##^*P* < 0.01 versus the model group

Based on the targets AKT, MMP-9, and COX-2 identified through network analysis and molecular docking, the pharmacological effects of apigenin were further experimentally validated. As shown in Fig. 10, model group markedly increased the expression of MMP-9, COX-2, as well as the level of phosphorylated AKT, in Caco-2 cells. Apigenin treatment reversed these changes, bringing the levels of these proteins closer to control values.

## Discussion

Apigenin, a naturally occurring flavonoid abundant in vegetables, fruits, and legumes, has attracted research interest due to its safety profile and therapeutic potential [34]. Our observations indicate that apigenin attenuates sepsis-induced intestinal barrier dysfunction, a critical complication of this life-threatening systemic infection [35]. In the initial stage of sepsis, the release of a large number of pro-inflammatory cytokines including TNF-α and IL-1β are associated with intestinal barrier impairment [36]. In addition, damage to the intestinal mucosal barrier allowing LPS to translocate into the bloodstream, leading to further complications associated with sepsis [37]. Therefore, protecting the integrity of the intestinal barrier is critical in preventing inflammation and reducing the risk of sepsis.

Experimental results indicated that apigenin administration was associated with reduced inflammatory cytokines and upregulated tight junction proteins (Occludin, Claudin-1, and ZO-1) in both septic mice and LPS-challenged Caco-2 cells [38]. These proteins are essential regulators for maintaining the structural and functional integrity of the intestinal barrier [39]. Functional assays revealed that apigenin intervention was associated with reduced FITC-dextran leakage and partial restoration of tight junction protein expression. These findings are consistent with a potential protective mechanism involving anti-inflammatory effects and junctional protein regulation.

Consistent with our study, previous research has also shown that LPS significantly decreases transepithelial electrical resistance TEER within 48 h [40]. Additionally, it reduces the levels of tight junction protein markers, including ZO-1, Occludin, and Claudin-1, at both the protein and mRNA levels [41]. The inflammatory cytokines induced by LPS disrupt the tight junction structure of intestinal epithelium. This finding underscores the importance of mitigating inflammatory injury, which holds critical implications for preventing sepsis-associated intestinal barrier dysfunction. To explore potential mechanisms, we first employed network pharmacology and molecular docking, which suggested AKT, MMP-9, and COX-2 as possible targets. Subsequent experiments showed apigenin treatment was associated with reduced expression of these markers in inflamed Caco-2 cells. While these results align with reports of apigenin reducing TNF-α, TGF-β, and IL-6 in TNBS/DSS models [42] and inhibiting NF-κB in IEC-6 cells [43]. Overall, our findings suggest that apigenin’s protective effect on the intestinal barrier is largely attributable to its anti-inflammatory action. Furthermore, apigenin’s anti-inflammatory properties extend beyond the gut to other organs as well. Specifically: (1) In the spleen, it significantly suppresses pro-inflammatory cytokine production through inhibition of the NF-κB signaling pathway [44]; (2) In brain tissue, this flavonoid effectively attenuates neuroinflammation by modulating microglial activation [45]; (3) For pancreatic function, apigenin demonstrates protective effects against diet-induced islet inflammation. These findings collectively elucidate the pleiotropic mechanisms underlying apigenin’s multi-organ anti-inflammatory actions [46]. These findings highlight the broad anti-inflammatory properties of apigenin and its potential for protecting various tissues and organs from inflammatory damage. In addition, we measured apigenin plasma concentrations using UPLC-MS/MS and found that it is well-absorbed with high bioavailability, showing 26.20 ng/ml at 2 h and 9.12 ng/ml at 24 h post-administration, indicating its potential therapeutic efficacy in reducing sepsis-related inflammatory damage.

This study on apigenin’s effects on sepsis-induced intestinal barrier damage has several limitations. Firstly, the sepsis mouse model has known differences from human sepsis, particularly in terms of immune responses and organ function. This highlights the need for more diverse animal models or clinical trials to validate the findings. Secondly, Caco-2 monocultures lack critical in vivo elements including immune-microbiome interactions. Given the rapid progression of sepsis, this study employed a prophylactic drug administration strategy. Apigenin was administered prior to LPS challenge to ensure stable plasma concentrations before inducing septic injury, thereby allowing evaluation of its effects on the intestinal barrier in mice. Additionally, post-modeling therapeutic intervention represents a critical avenue for further investigation. The study primarily examines short-term effects, overlooking the long-term impact of apigenin on sepsis recovery. Furthermore, the optimal dosage and potential toxicity in humans remain unaddressed, requiring further investigation. While network pharmacology can predict drug interactions, we also conduct validation at the cellular level, as it may oversimplify molecular interactions. Future research should include clinical trials, long-term studies, and deeper exploration of molecular mechanisms, optimal dosage, and combination therapies.

Although the LPS-induced endotoxemia is commonly used, some studies have also chosen the CLP model [47, 48]. The CLP model more accurately replicates the pathophysiology of human sepsis through polymicrobial infection and a progressive disease course, in contrast to LPS models, which rely solely on endotoxin-induced hyperinflammation. CLP offers greater translational value by mirroring clinical features such as bacteremia and organ dysfunction, allowing for the evaluation of both preventive and therapeutic interventions across different stages of sepsis. Although more technically demanding than LPS models, the physiological relevance of CLP justifies its use. Moreover, combining both models could provide complementary insights into drug efficacy. In our future studies, we may choose both models to investigate the protective effects and mechanisms of compounds.

Our data collectively suggest that apigenin may help maintain intestinal barrier function during sepsis, potentially interrupting the cycle of bacterial translocation and systemic inflammation [5]. Current evidence demonstrates that sepsis-induced activation of the AKT signaling pathway leads to upregulation of downstream effectors including MMP-9 and COX-2, accompanied by elevated inflammatory cytokine levels, ultimately contributing to intestinal barrier dysfunction and multi-organ injury [49, 50]. Our results suggest that apigenin’s protective effects are at least partly mediated by modulation of the PI3K-AKT signaling pathway. Our integrated approach combining network pharmacology and experimental validation reveals apigenin exerts protection through a sequential mechanism: By primarily inhibiting AKT activation, apigenin secondarily reduces expression of MMP-9 and COX-2. This downstream effect then attenuates the inflammatory response, consequently preserving intestinal barrier function and preventing sepsis-induced damage. Although the current study has preliminarily demonstrated through network pharmacology analysis, molecular docking, and assessment of AKT phosphorylation that apigenin’s protective effects correlate with attenuated AKT activation, we recognize that these findings require further validation. The absence of interventional approaches using either AKT pharmacological inhibitors or genetic manipulation represents a key limitation in establishing a definitive mechanistic relationship.

In our future studies, we will explore the impact of various administration methods on septic-induced intestinal barrier damage. The evaluation of systemic sepsis markers, blood lactate levels, and organ function indices will be essential for improving sepsis diagnosis, risk stratification, and therapeutic monitoring. Additionally, we anticipate that the pharmacokinetic profile of apigenin may be altered under septic conditions, an aspect we intend to examine in subsequent research. Building upon the findings of this study, we will further investigate the effects and underlying mechanisms of other flavonoid-like compounds targeting this pathway. Moreover, considering the pivotal role of gut microbiota in both the pathogenesis and protection of the intestinal barrier, we will incorporate this factor into our future investigations. Notably, the limitations of the present study, including the absence of long-term outcome data, dose-response assessments, and toxicity evaluations, will be addressed in subsequent experiments to enhance the robustness of our findings.

## Supplementary Information

Below is the link to the electronic supplementary material.

Supplementary Material 1

Supplementary Material 2

Supplementary Material 3

## Data Availability

Data is provided within the supplementary information files.

## Abbreviations

AKT1: AKT serine/threonine kinase 1
API: Apigenin
BSA: Bovine Serum Albumin
COX-2: Cyclooxygenase-2
CTD: Comparative Toxicogenomics Database
IL-6: Interleukin-6
IL-10: Interleukin-10
MMP9: Matrix Metalloproteinase-9
NS: Normal Saline
PPI: Protein-Protein Interaction
SIRS: Systemic Inflammatory Response Syndrome
TEER: Transepithelial Electrical Resistance
TGF-β: Transforming Growth Factor-beta
TNF-α: Tumor Necrosis Factor-alpha
ZO-1: Zonula Occludens-1
