# Supplementary figures and images for: Apigenin mitigates intestinal barrier dysfunction in sepsis by modulating the AKT signaling pathway
Source: BMC Gastroenterol. 2025 Aug 30;25:626. doi: 10.1186/s12876-025-04196-0 (PMC12398084; doi:10.1186/s12876-025-04196-0)

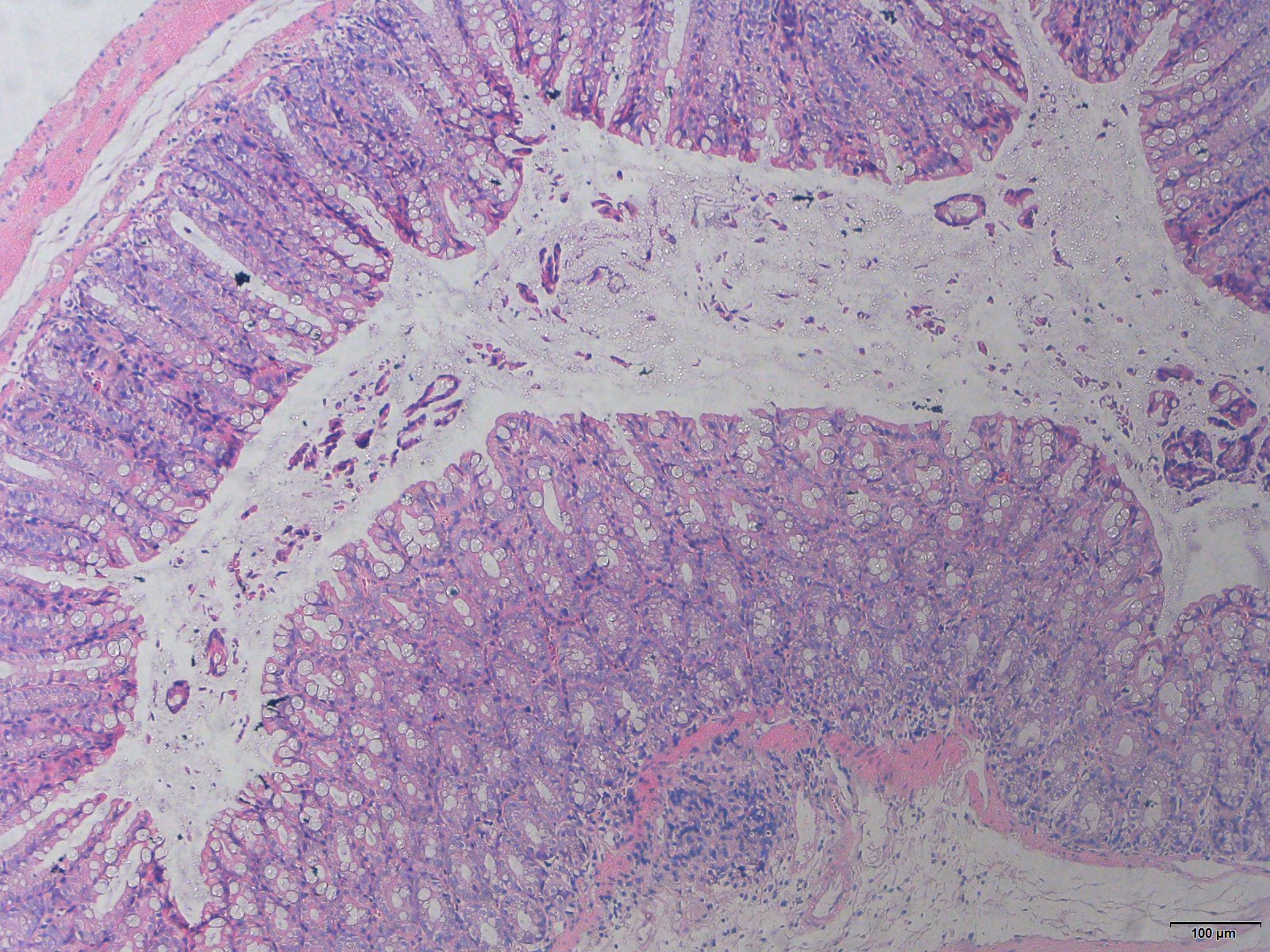

Supplement: Supplementary file 1 — Supplementary Material 1 [file 12876_2025_4196_MOESM1_ESM.zip › Supplementary Materials/Figure 1/D/LPS/LPS-10X.tif]

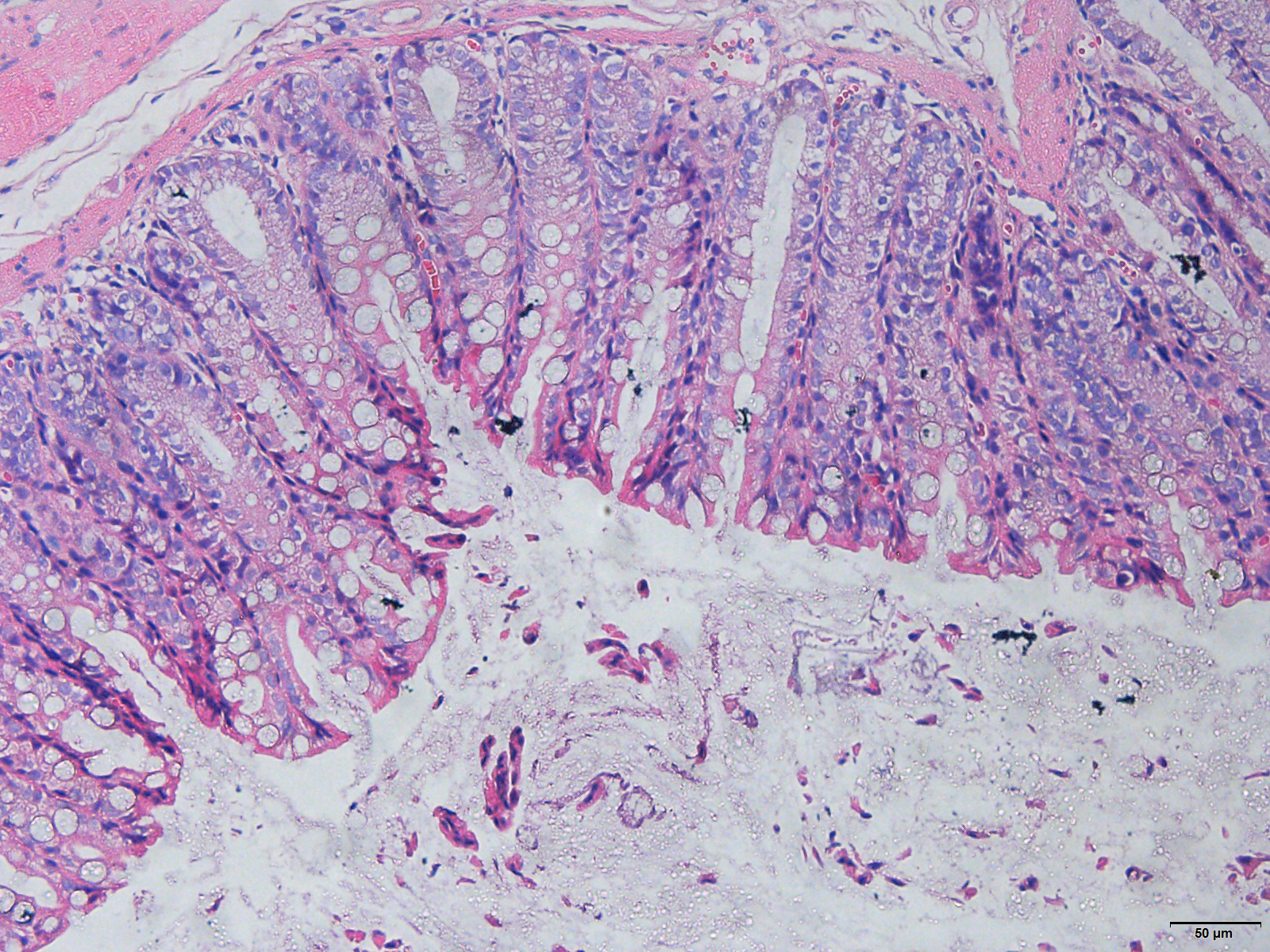

Supplement: Supplementary file 1 — Supplementary Material 1 [file 12876_2025_4196_MOESM1_ESM.zip › Supplementary Materials/Figure 1/D/LPS/LPS-20X.tif]

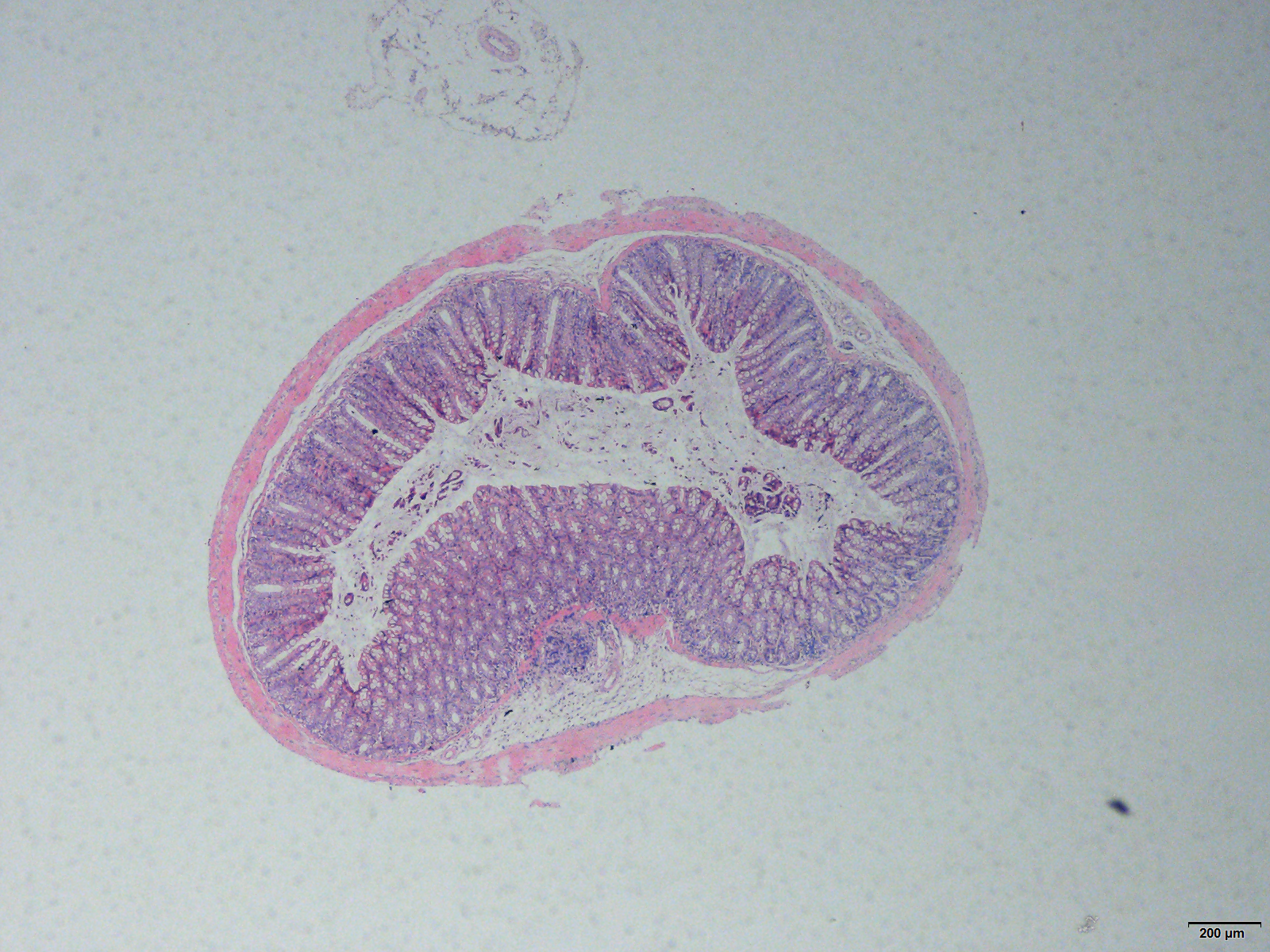

Supplement: Supplementary file 1 — Supplementary Material 1 [file 12876_2025_4196_MOESM1_ESM.zip › Supplementary Materials/Figure 1/D/LPS/LPS-4X.tif]

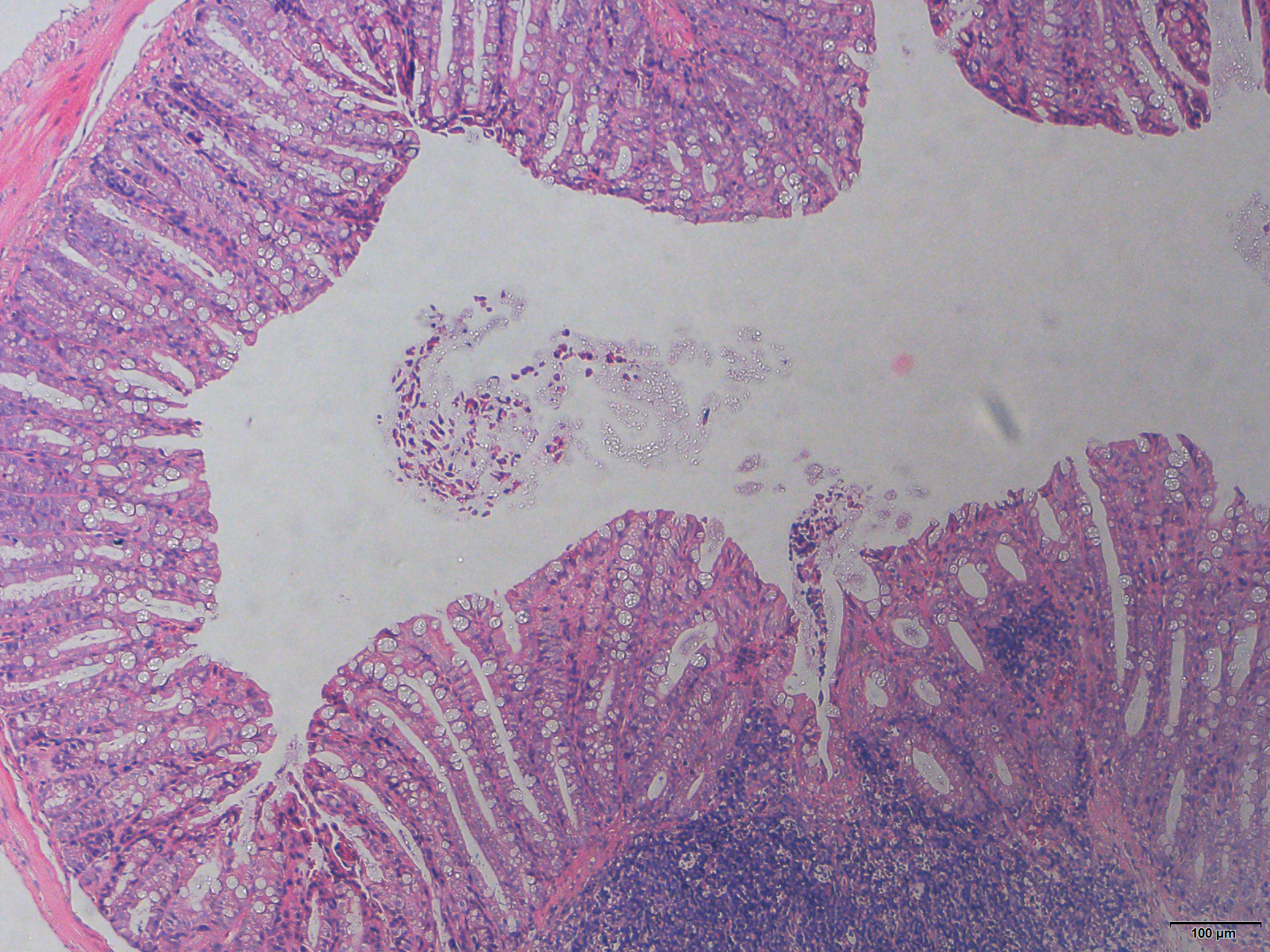

Supplement: Supplementary file 1 — Supplementary Material 1 [file 12876_2025_4196_MOESM1_ESM.zip › Supplementary Materials/Figure 1/D/LPS+API/API-10X.tif]

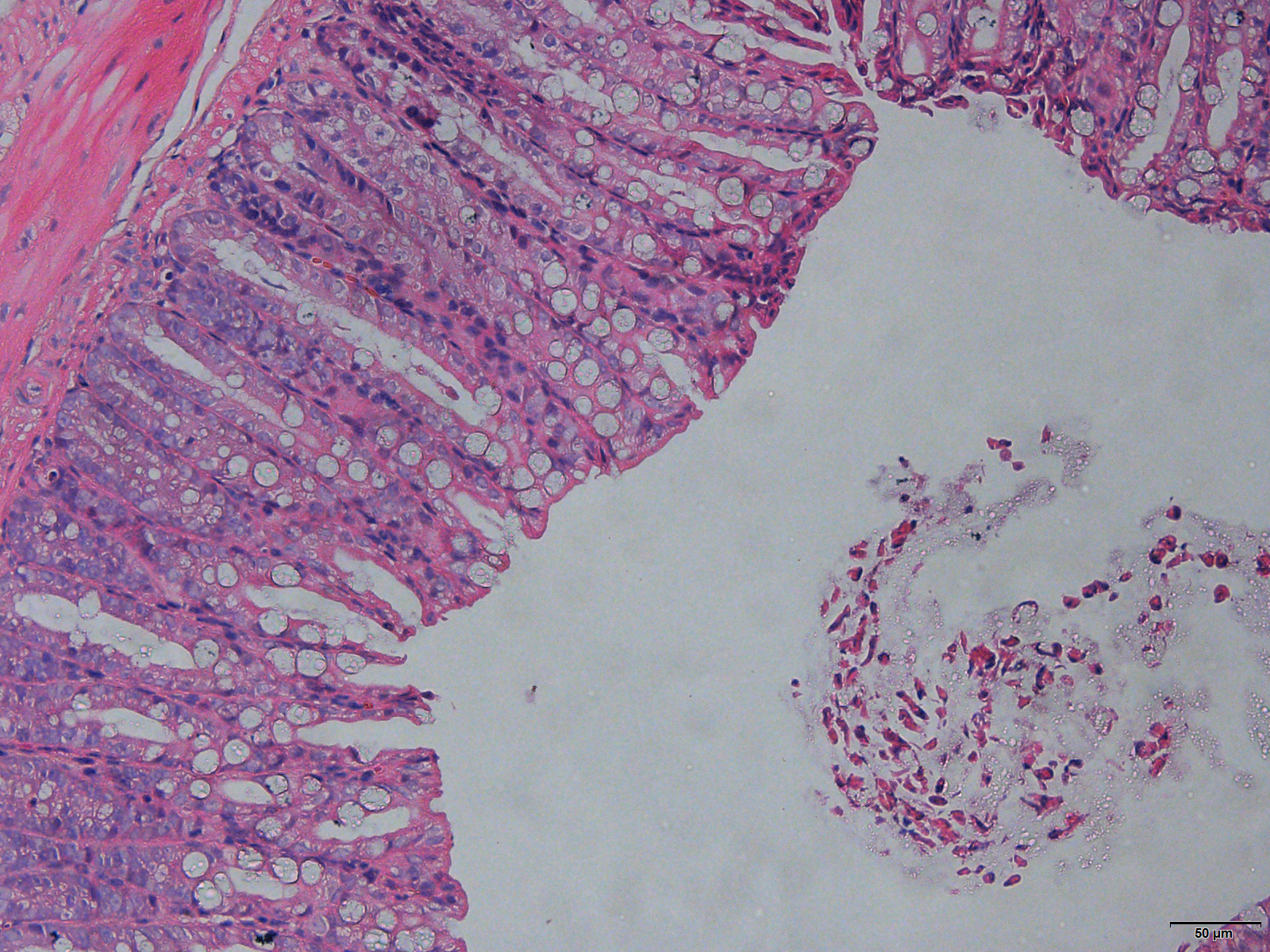

Supplement: Supplementary file 1 — Supplementary Material 1 [file 12876_2025_4196_MOESM1_ESM.zip › Supplementary Materials/Figure 1/D/LPS+API/API-20X.tif]

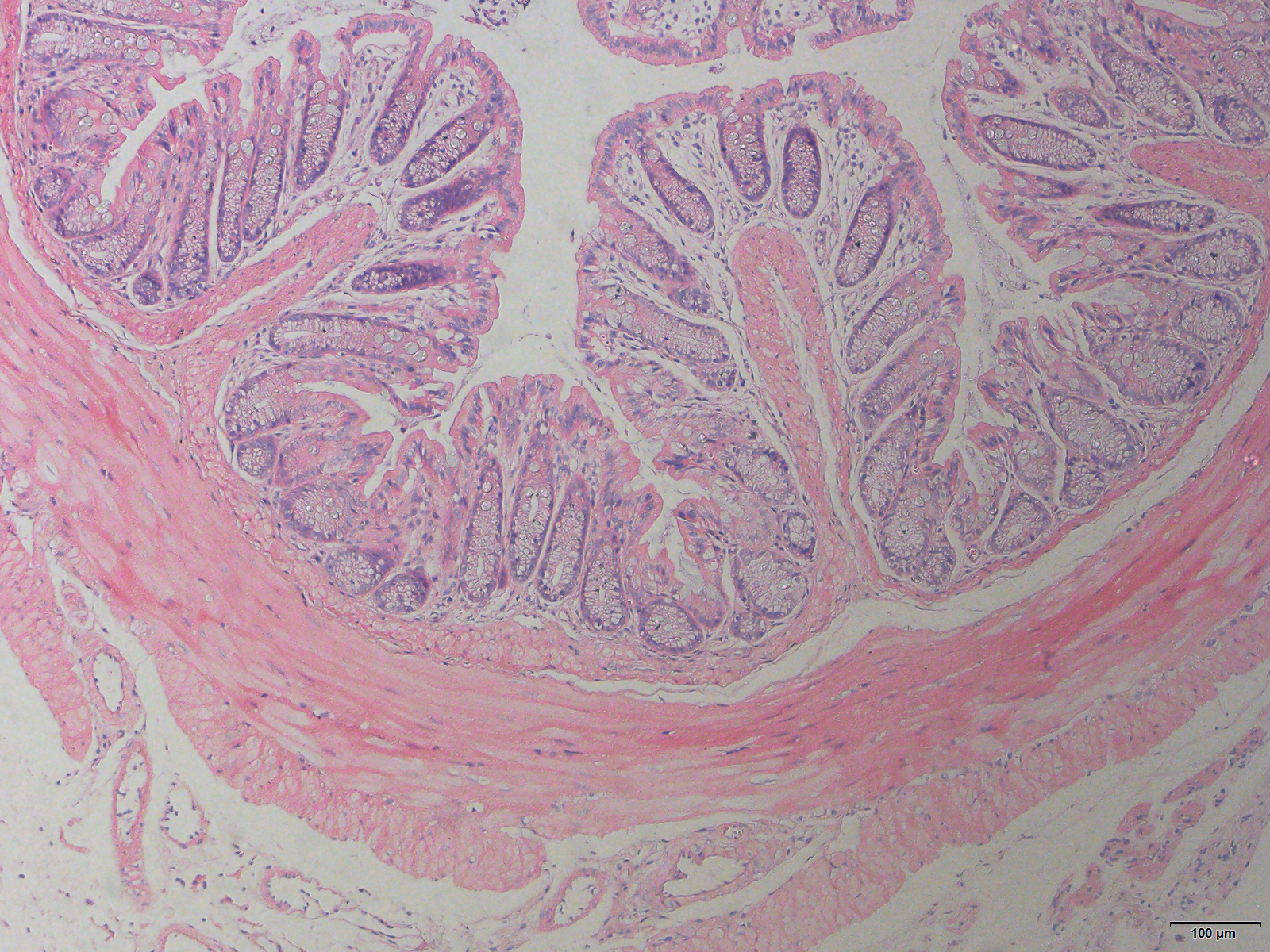

Supplement: Supplementary file 1 — Supplementary Material 1 [file 12876_2025_4196_MOESM1_ESM.zip › Supplementary Materials/Figure 1/D/N/N-10X.tif]

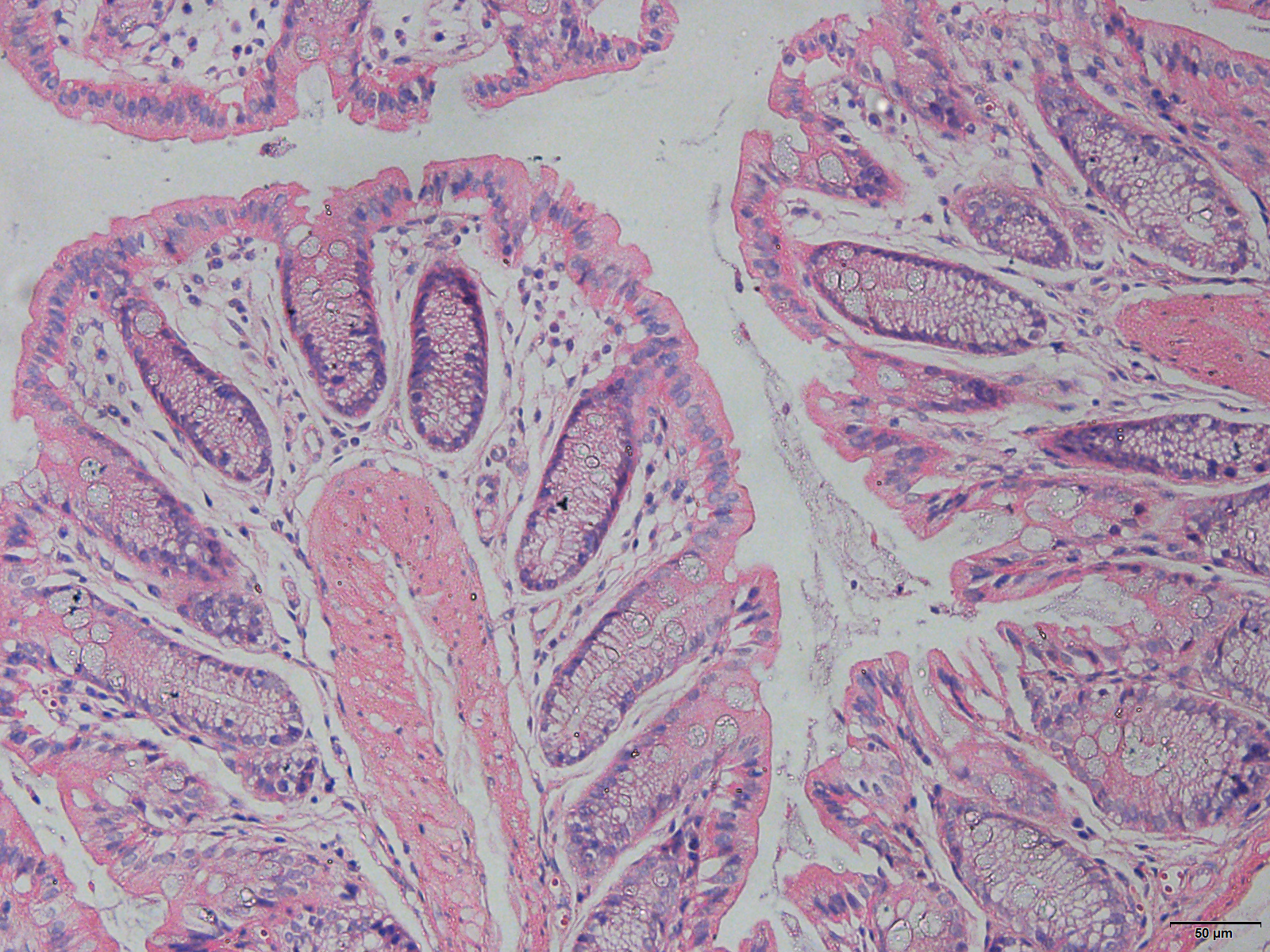

Supplement: Supplementary file 1 — Supplementary Material 1 [file 12876_2025_4196_MOESM1_ESM.zip › Supplementary Materials/Figure 1/D/N/N-20X.tif]

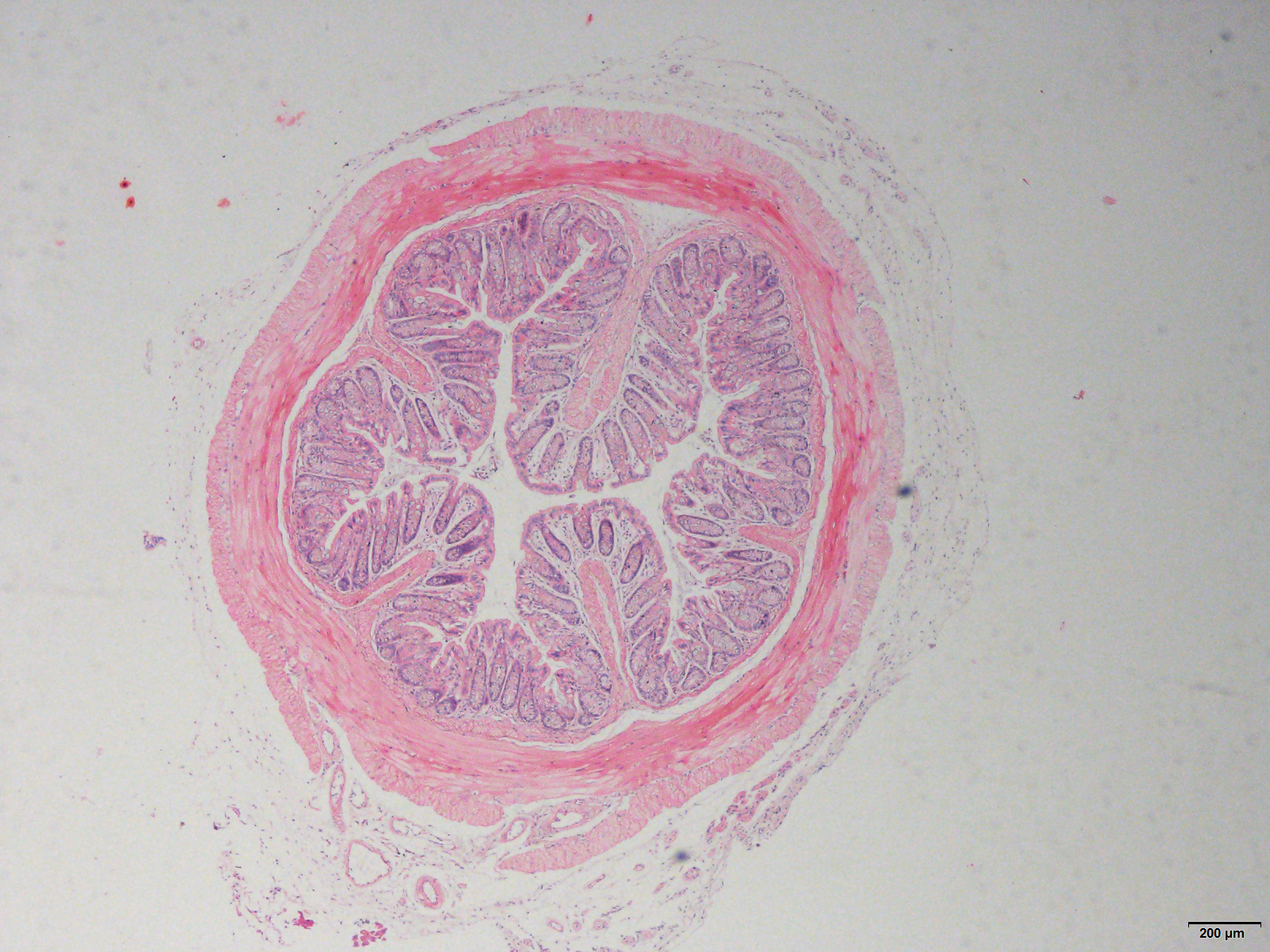

Supplement: Supplementary file 1 — Supplementary Material 1 [file 12876_2025_4196_MOESM1_ESM.zip › Supplementary Materials/Figure 1/D/N/N-4X.tif]

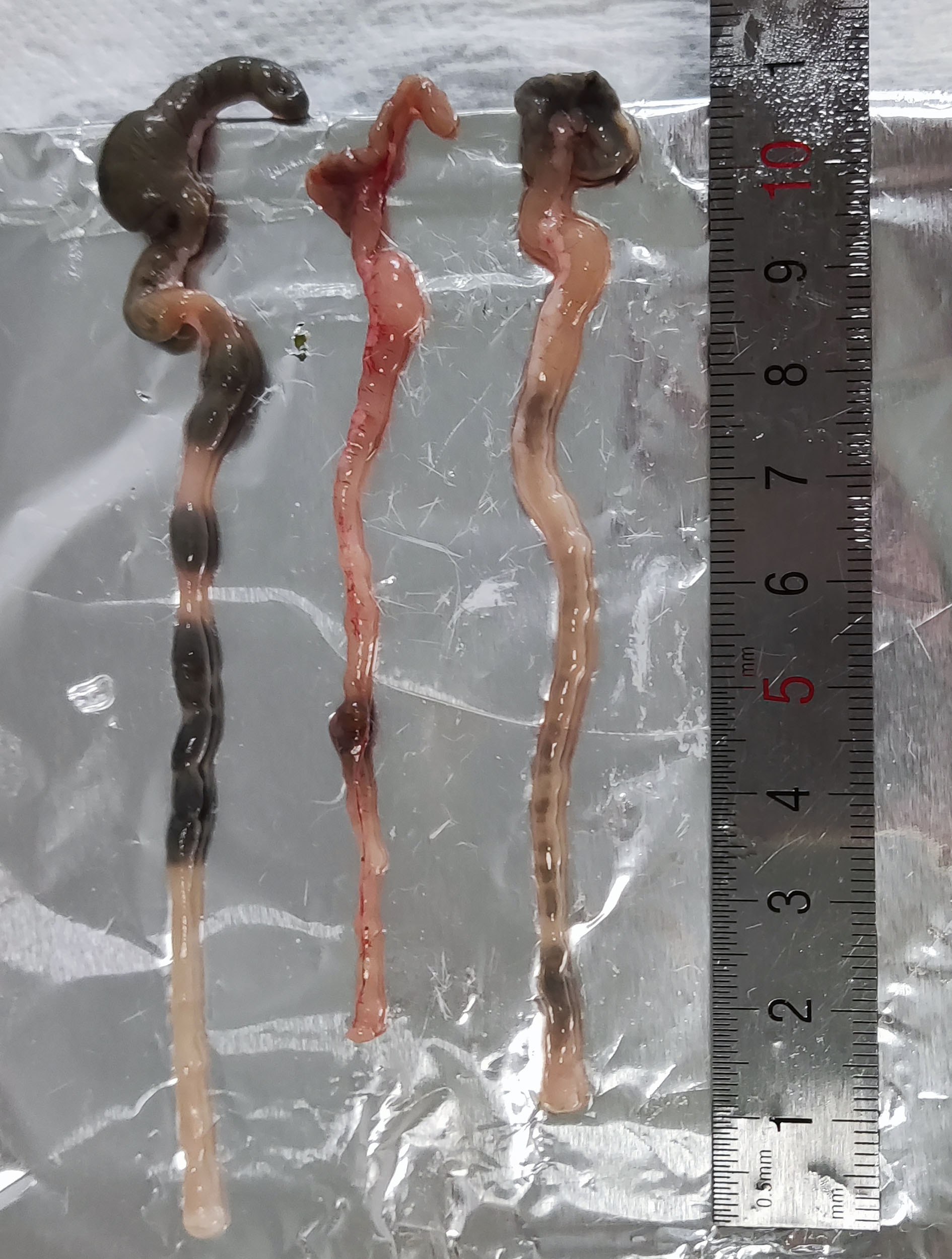

Supplement: Supplementary file 1 — Supplementary Material 1 [file 12876_2025_4196_MOESM1_ESM.zip › Supplementary Materials/Figure 1/Figure1-A.jpg]

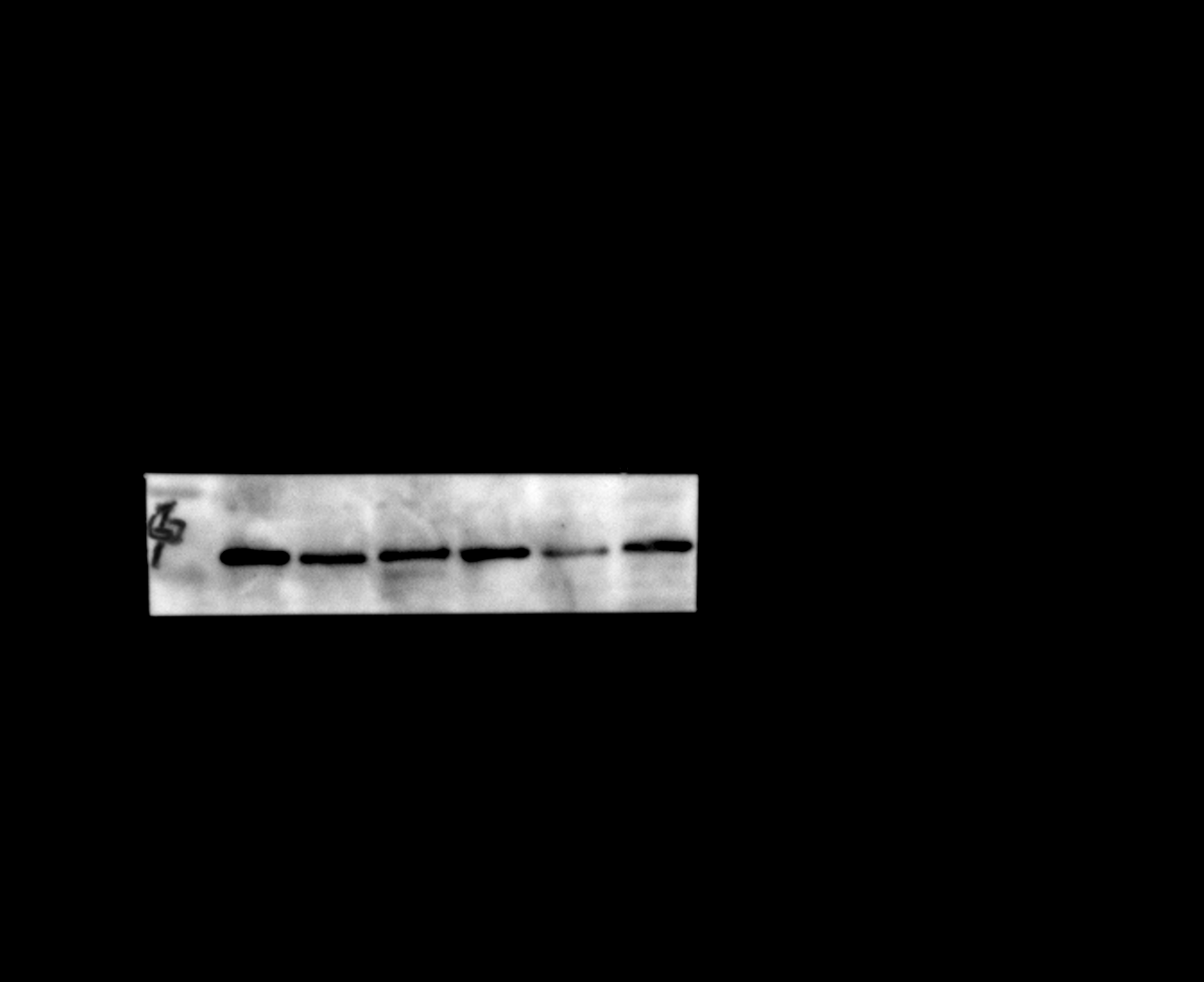

Supplement: Supplementary file 1 — Supplementary Material 1 [file 12876_2025_4196_MOESM1_ESM.zip › Supplementary Materials/Figure 3/G/Figure3-GAPDH1-1.Tif]

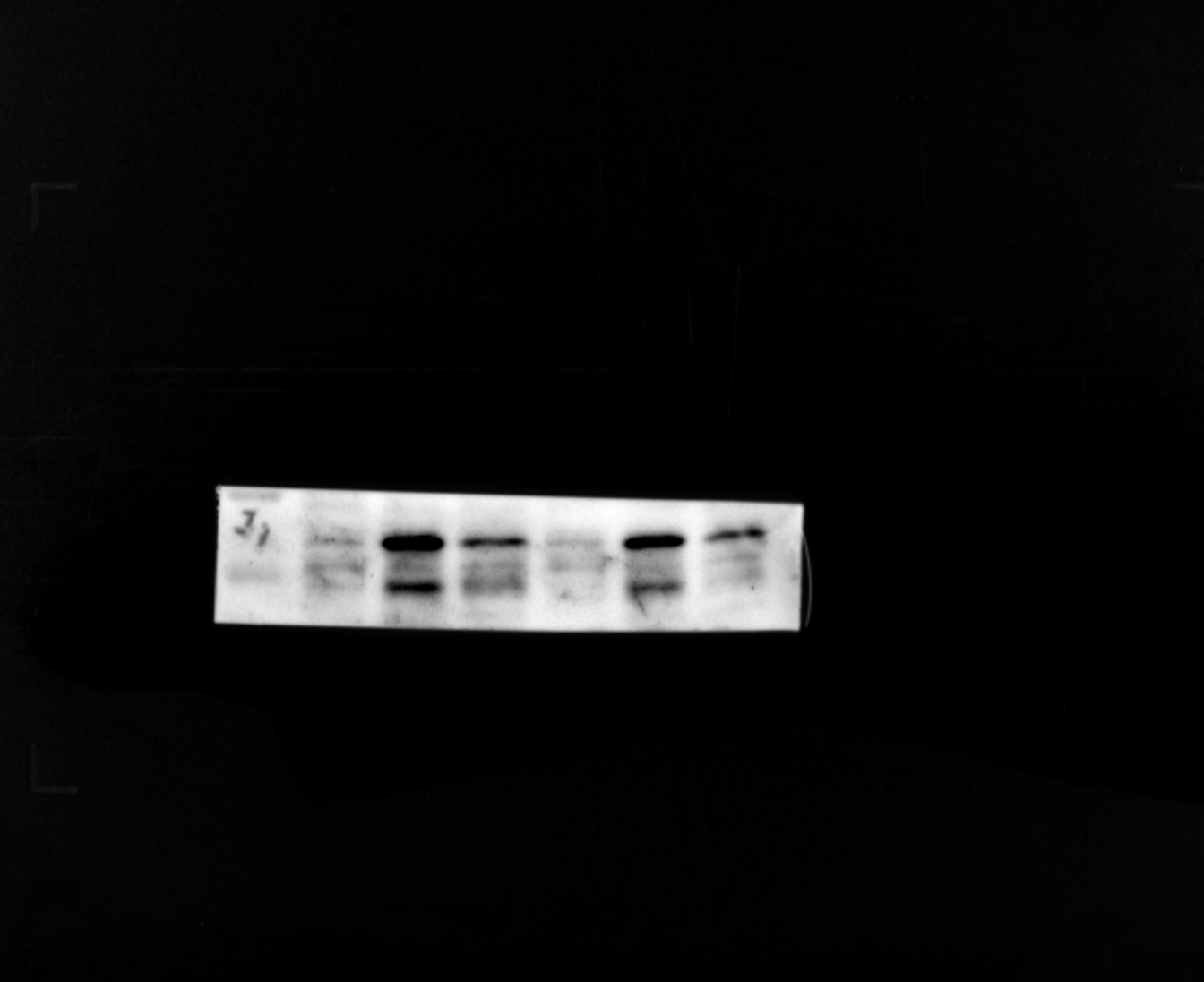

Supplement: Supplementary file 1 — Supplementary Material 1 [file 12876_2025_4196_MOESM1_ESM.zip › Supplementary Materials/Figure 3/G/Figure3-IL-1β.Tif]

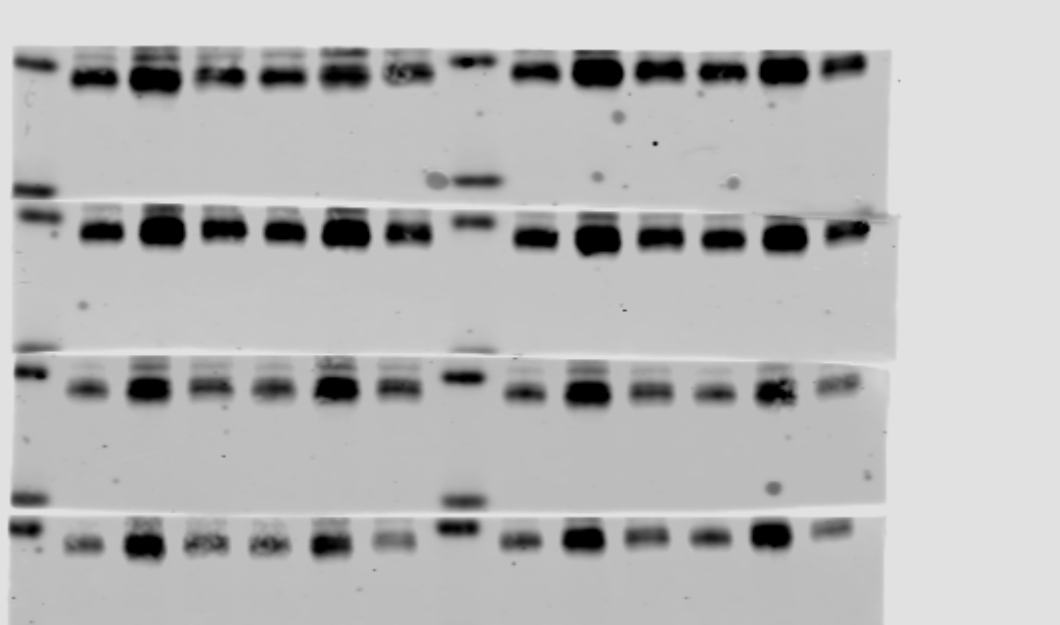

Supplement: Supplementary file 1 — Supplementary Material 1 [file 12876_2025_4196_MOESM1_ESM.zip › Supplementary Materials/Figure 3/G/Figure3-IL-6.tif]

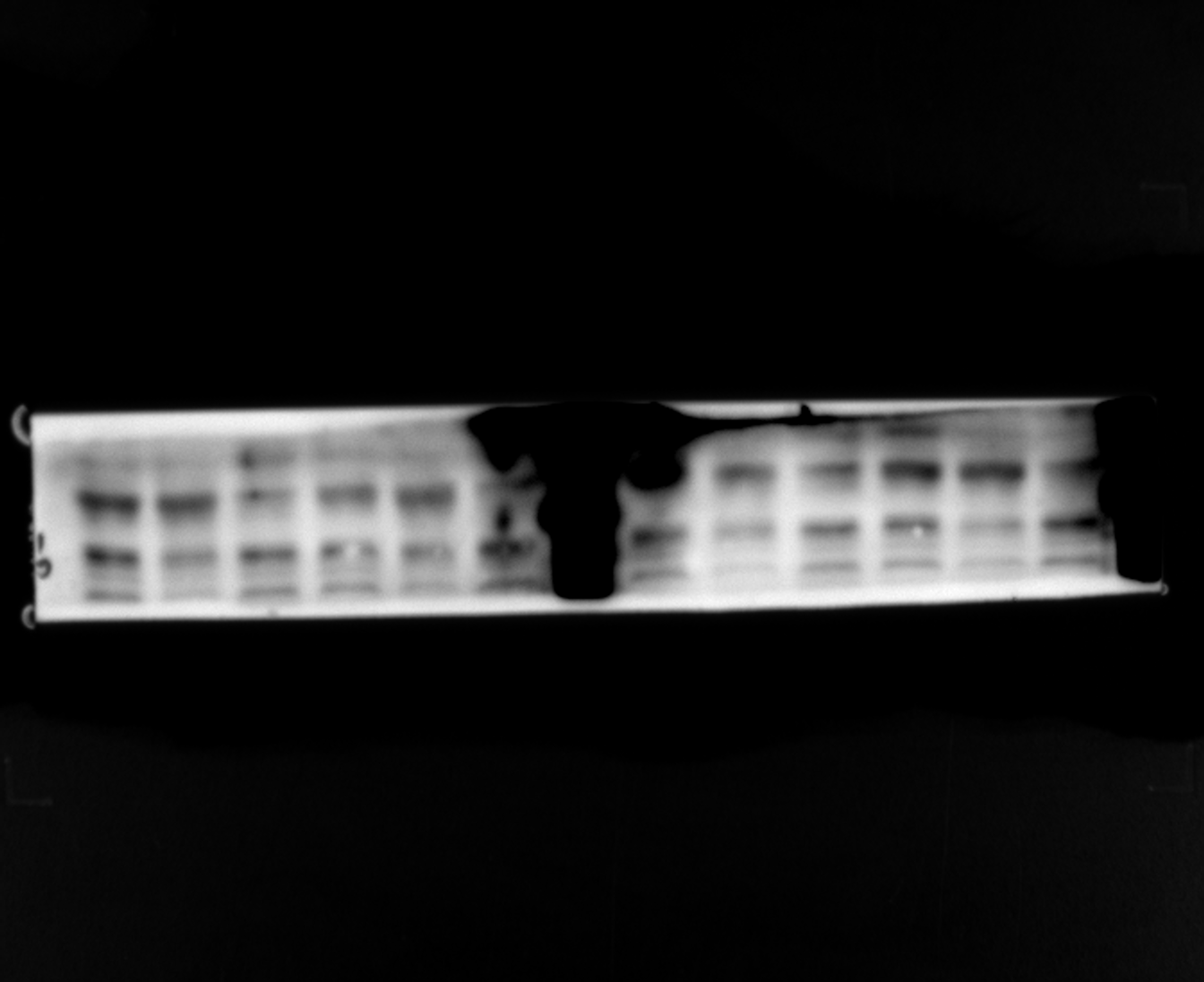

Supplement: Supplementary file 1 — Supplementary Material 1 [file 12876_2025_4196_MOESM1_ESM.zip › Supplementary Materials/Figure 4/Figure4-G/Figure4-Claudin1.Tif]

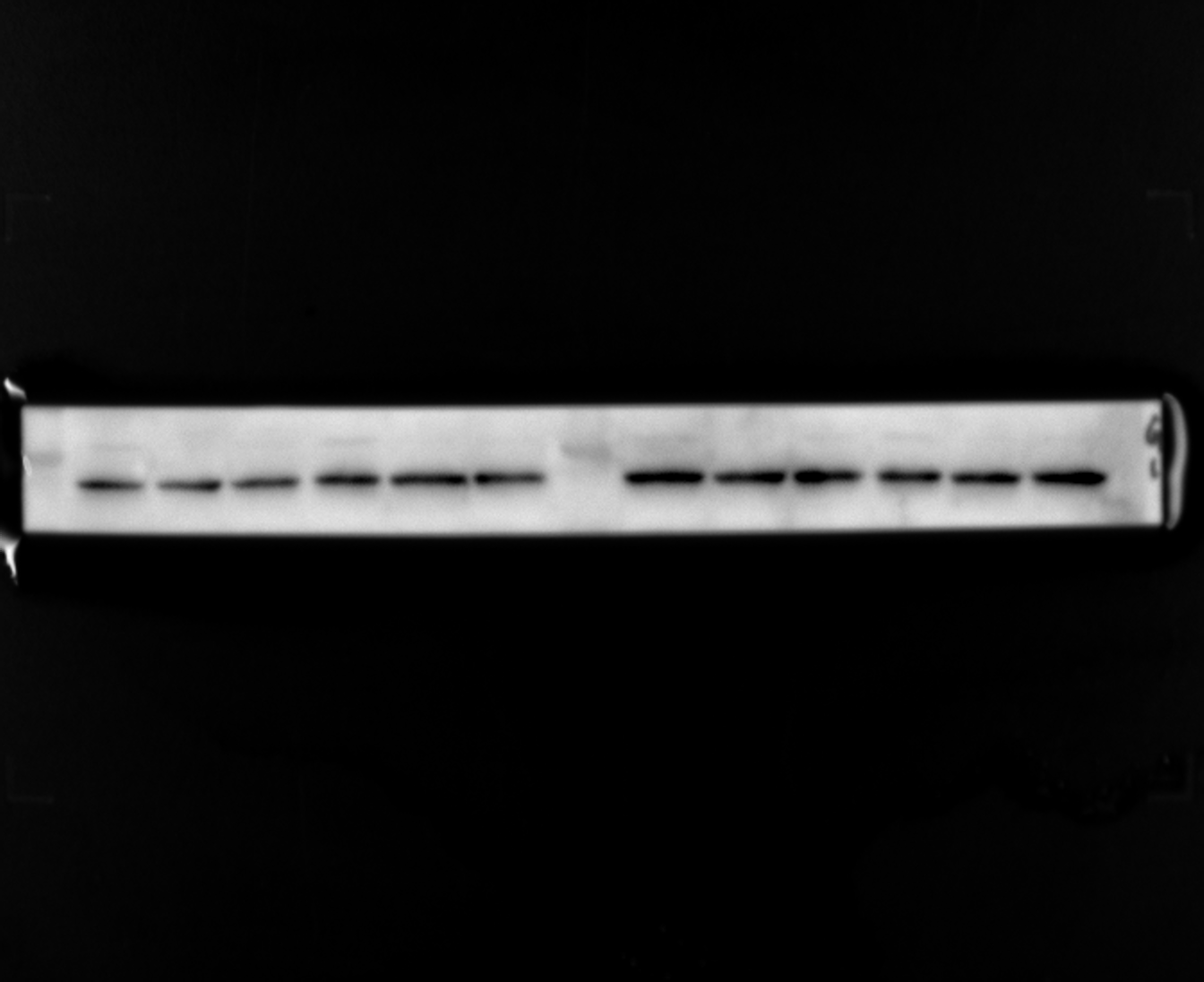

Supplement: Supplementary file 1 — Supplementary Material 1 [file 12876_2025_4196_MOESM1_ESM.zip › Supplementary Materials/Figure 4/Figure4-G/Figure4-GAPDH1.Tif]

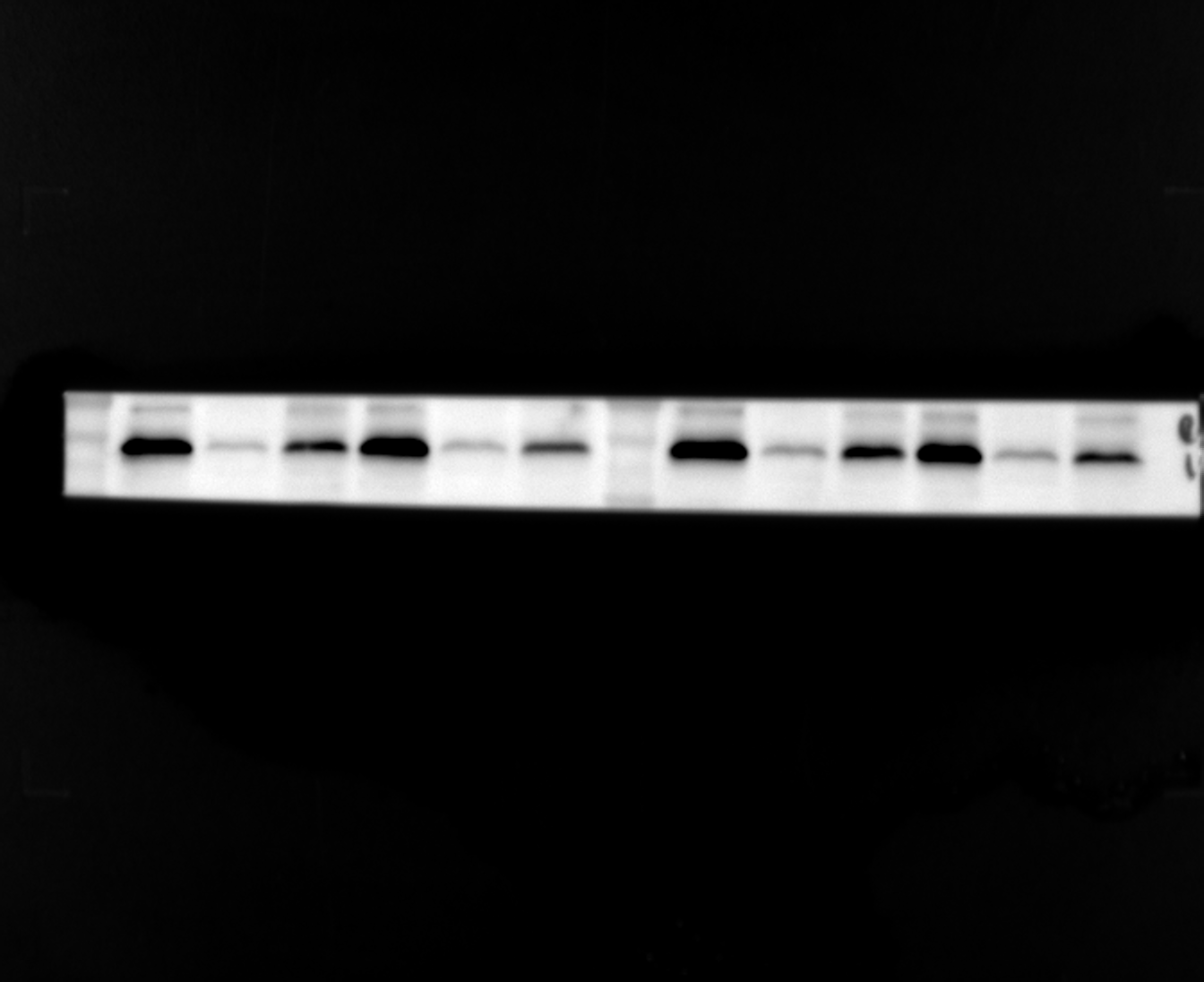

Supplement: Supplementary file 1 — Supplementary Material 1 [file 12876_2025_4196_MOESM1_ESM.zip › Supplementary Materials/Figure 4/Figure4-G/Figure4-Occludin1.Tif]

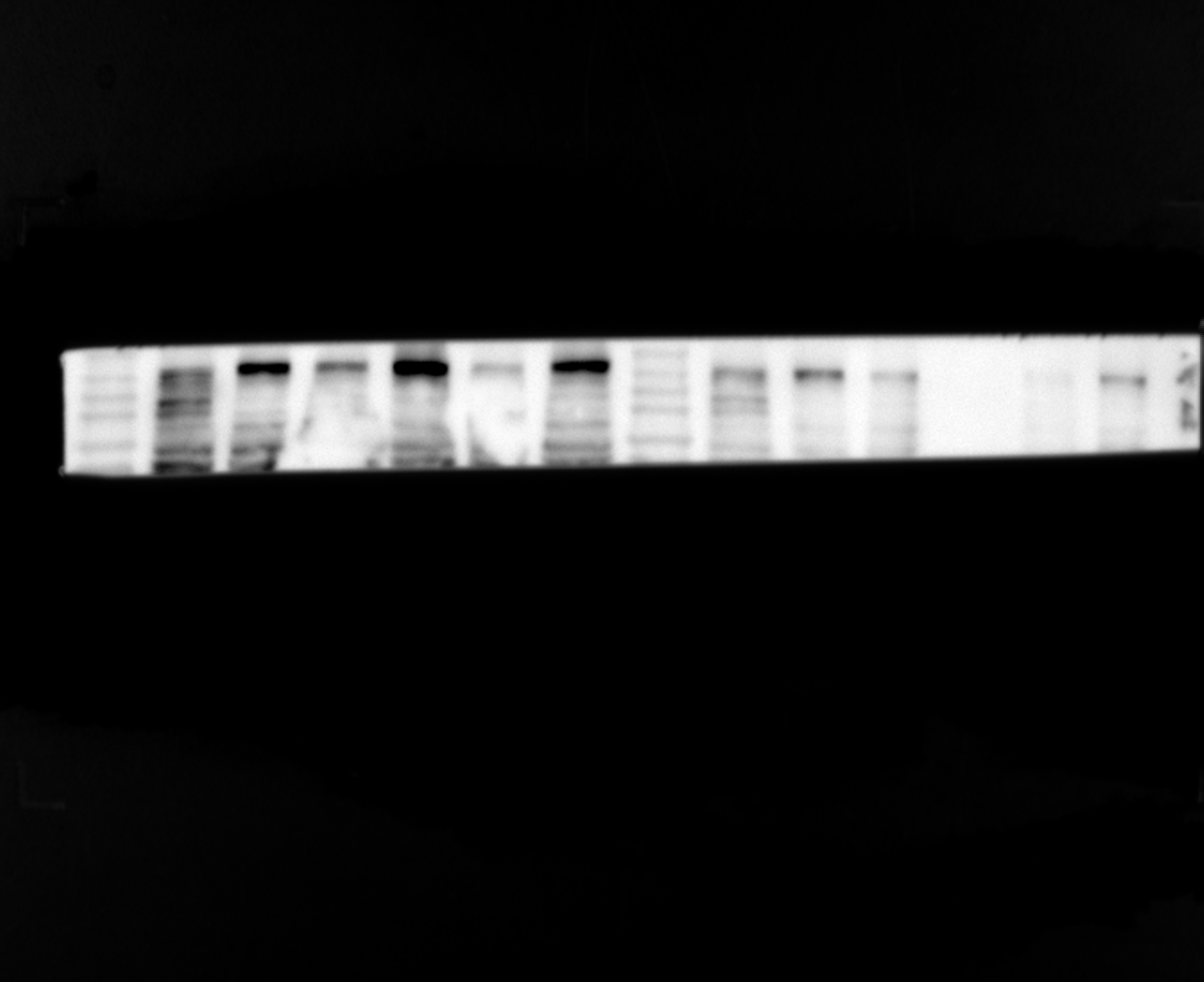

Supplement: Supplementary file 1 — Supplementary Material 1 [file 12876_2025_4196_MOESM1_ESM.zip › Supplementary Materials/Figure 4/Figure4-G/Figure4-ZO-1.Tif]

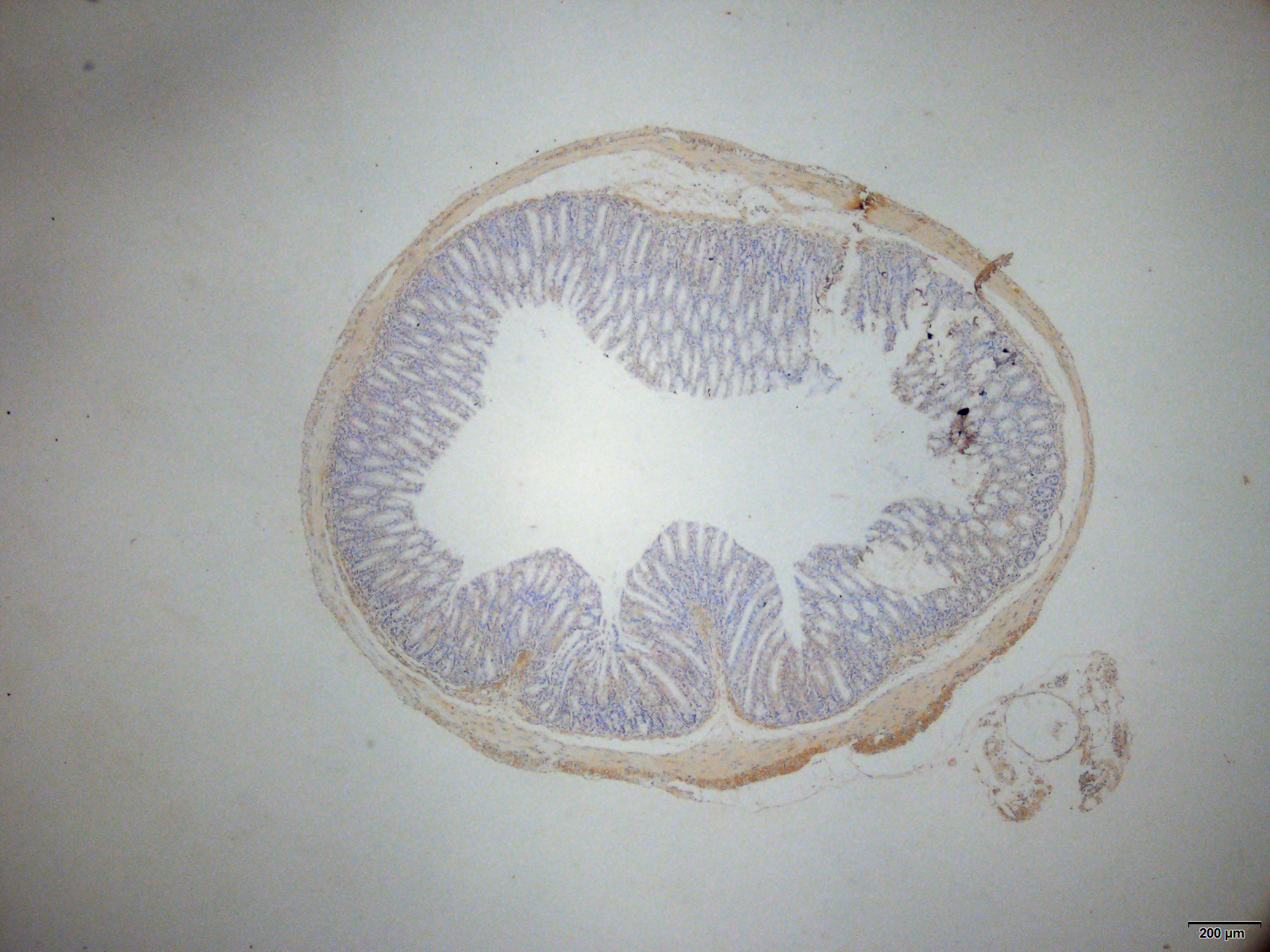

Supplement: Supplementary file 1 — Supplementary Material 1 [file 12876_2025_4196_MOESM1_ESM.zip › Supplementary Materials/Figure 4/Figure4-H/Figure4-API-1.tif]

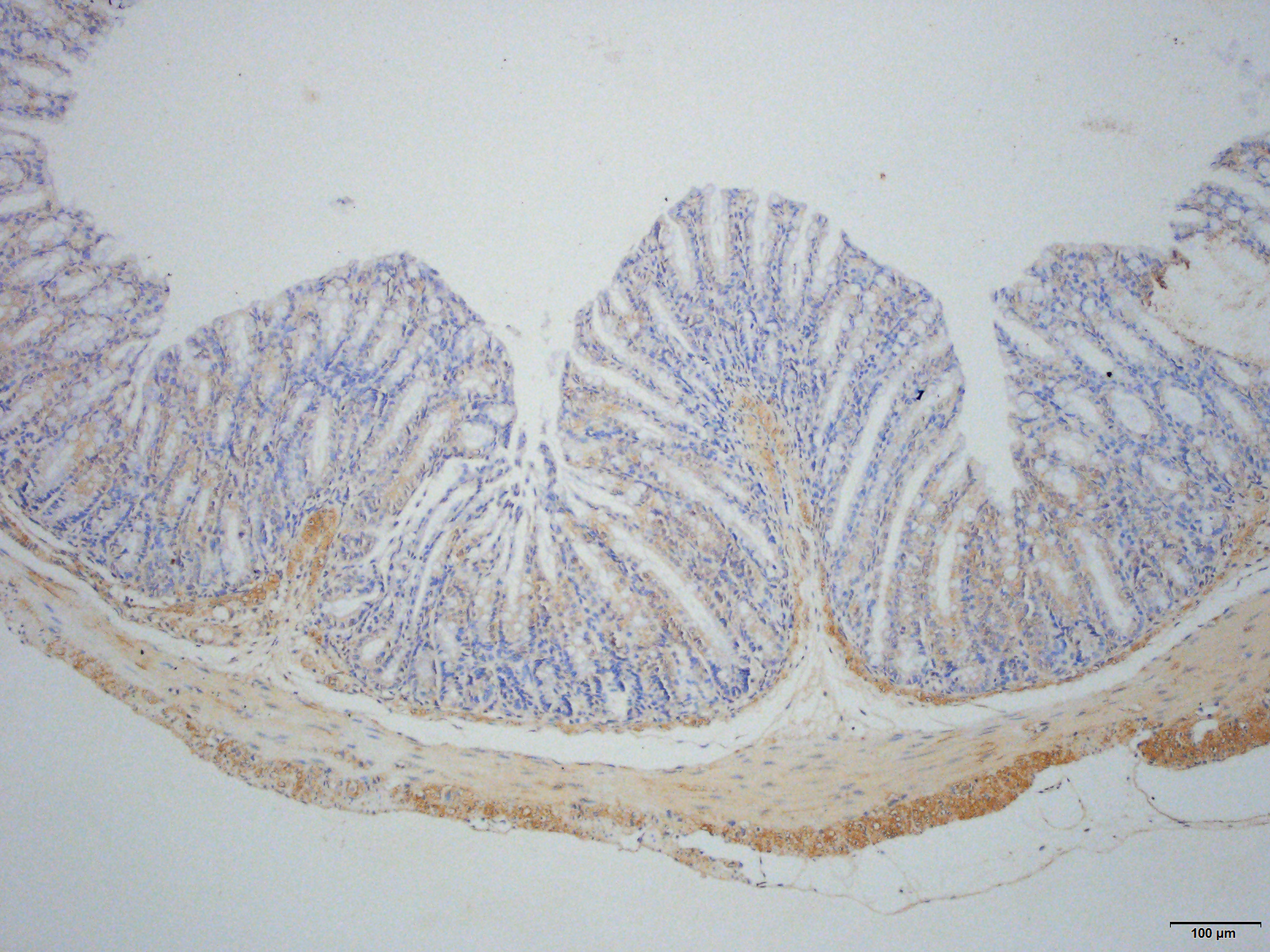

Supplement: Supplementary file 1 — Supplementary Material 1 [file 12876_2025_4196_MOESM1_ESM.zip › Supplementary Materials/Figure 4/Figure4-H/Figure4-API-2.tif]

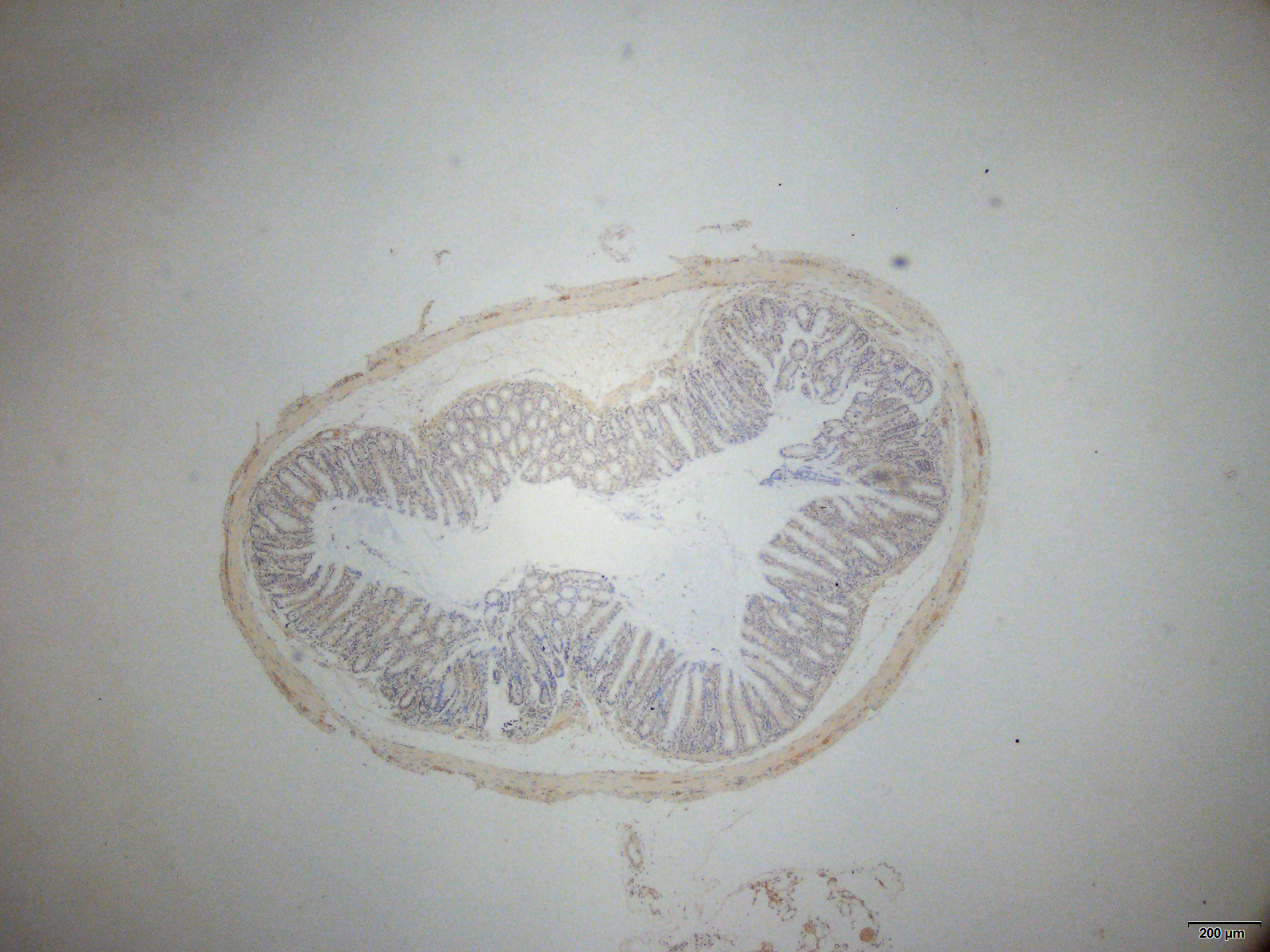

Supplement: Supplementary file 1 — Supplementary Material 1 [file 12876_2025_4196_MOESM1_ESM.zip › Supplementary Materials/Figure 4/Figure4-H/Figure4-LPS-1.tif]

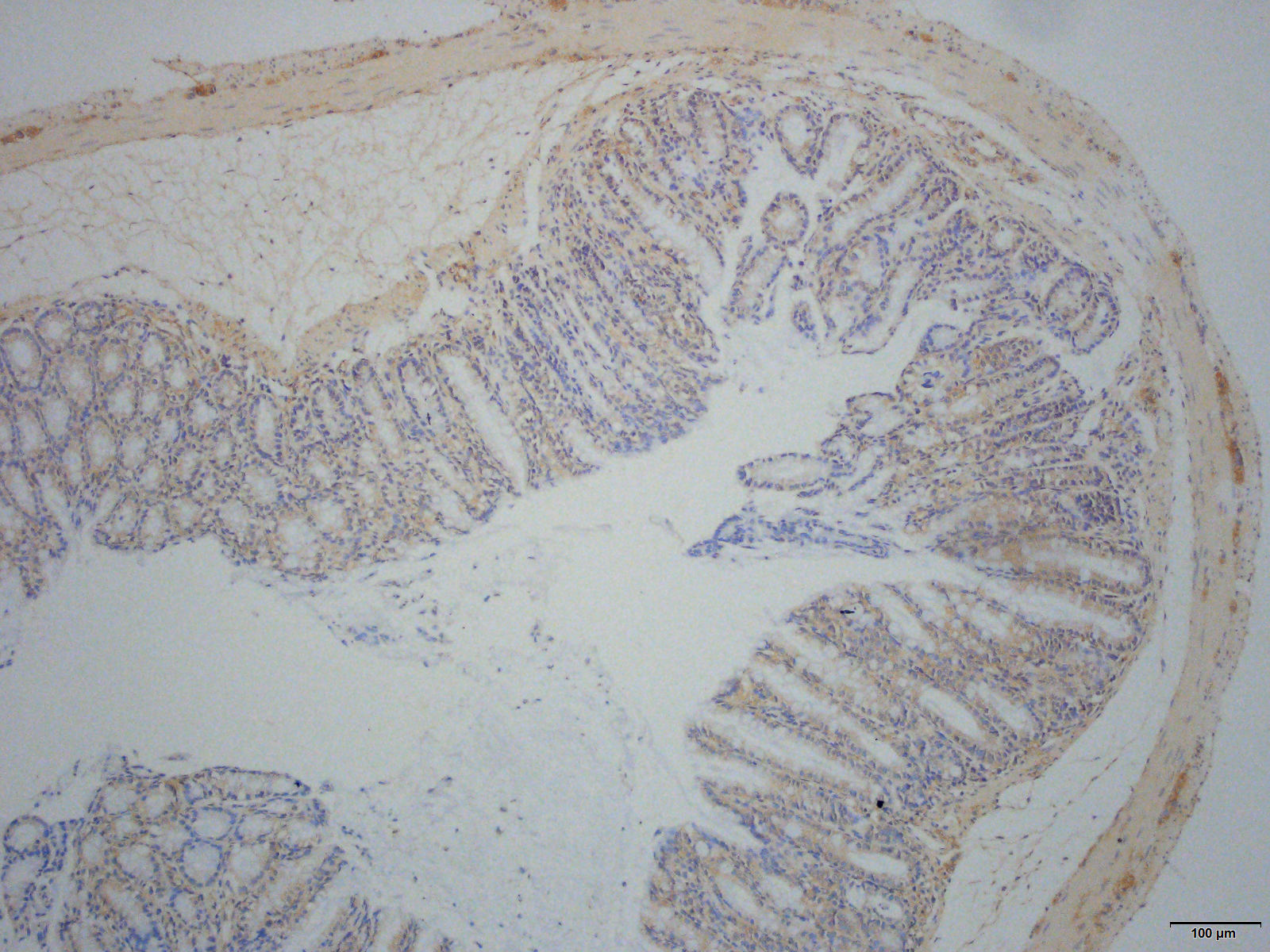

Supplement: Supplementary file 1 — Supplementary Material 1 [file 12876_2025_4196_MOESM1_ESM.zip › Supplementary Materials/Figure 4/Figure4-H/Figure4-LPS-2.tif]

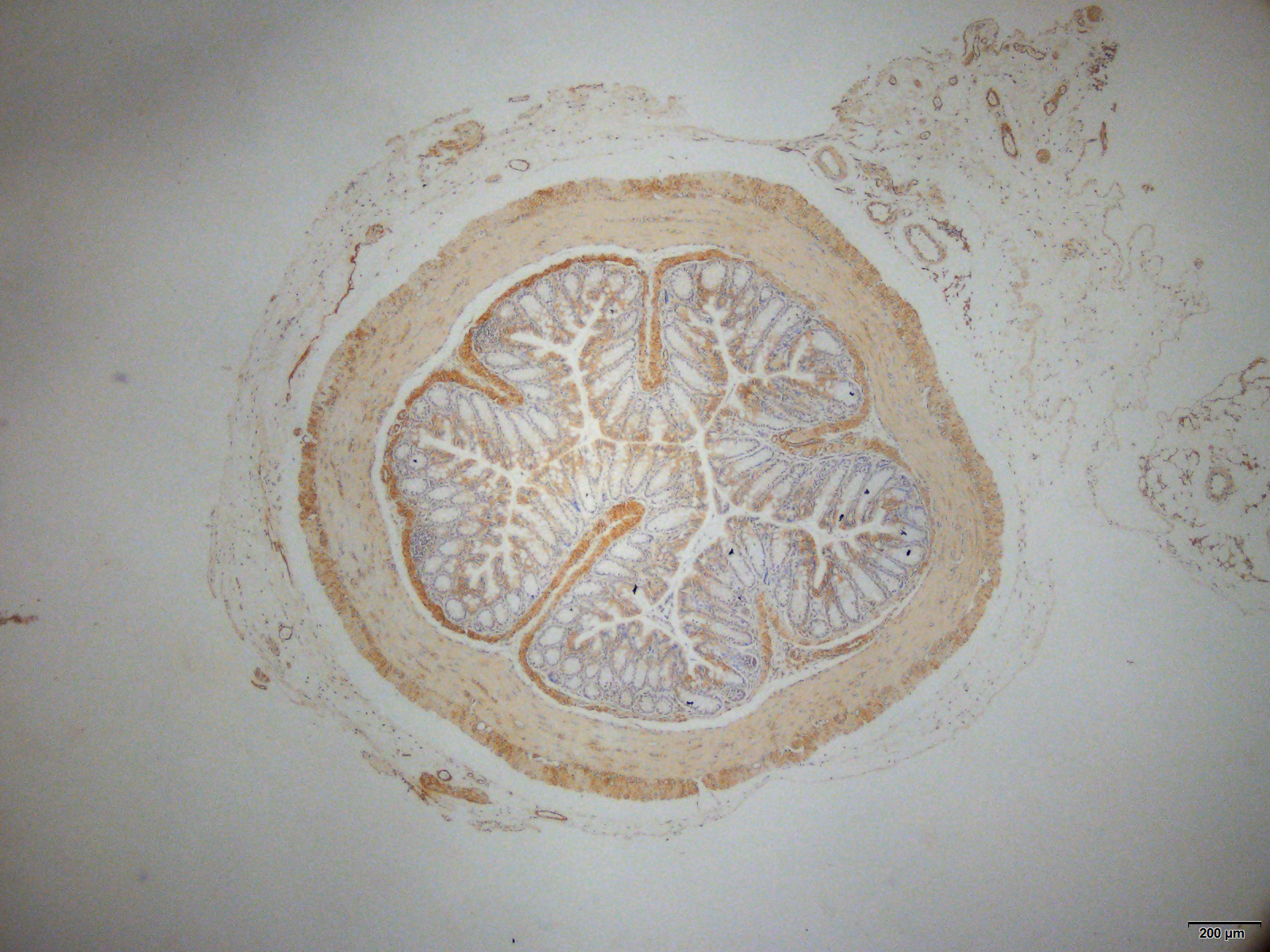

Supplement: Supplementary file 1 — Supplementary Material 1 [file 12876_2025_4196_MOESM1_ESM.zip › Supplementary Materials/Figure 4/Figure4-H/Figure4-N-1.tif]

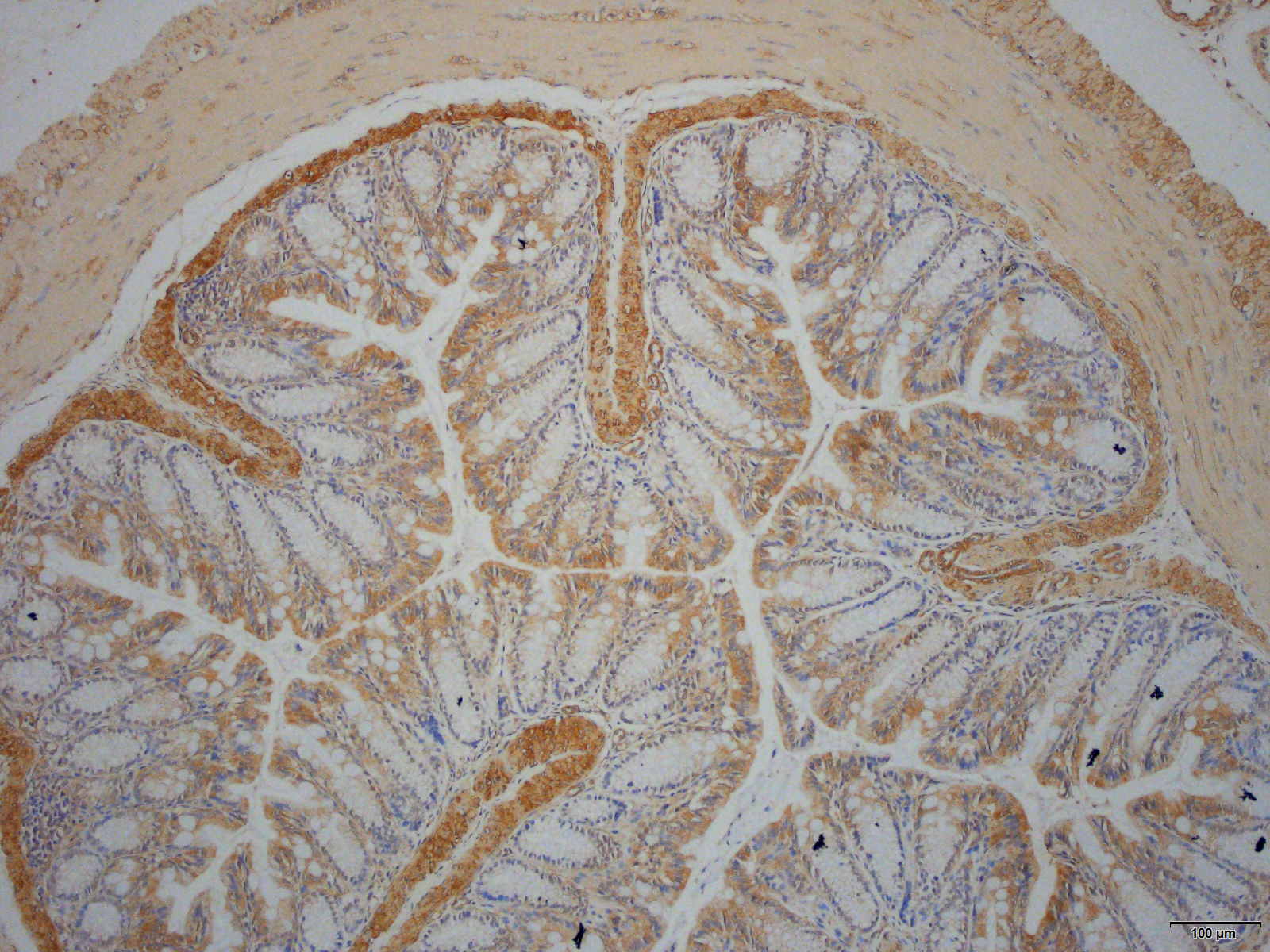

Supplement: Supplementary file 1 — Supplementary Material 1 [file 12876_2025_4196_MOESM1_ESM.zip › Supplementary Materials/Figure 4/Figure4-H/Figure4-N-2.tif]

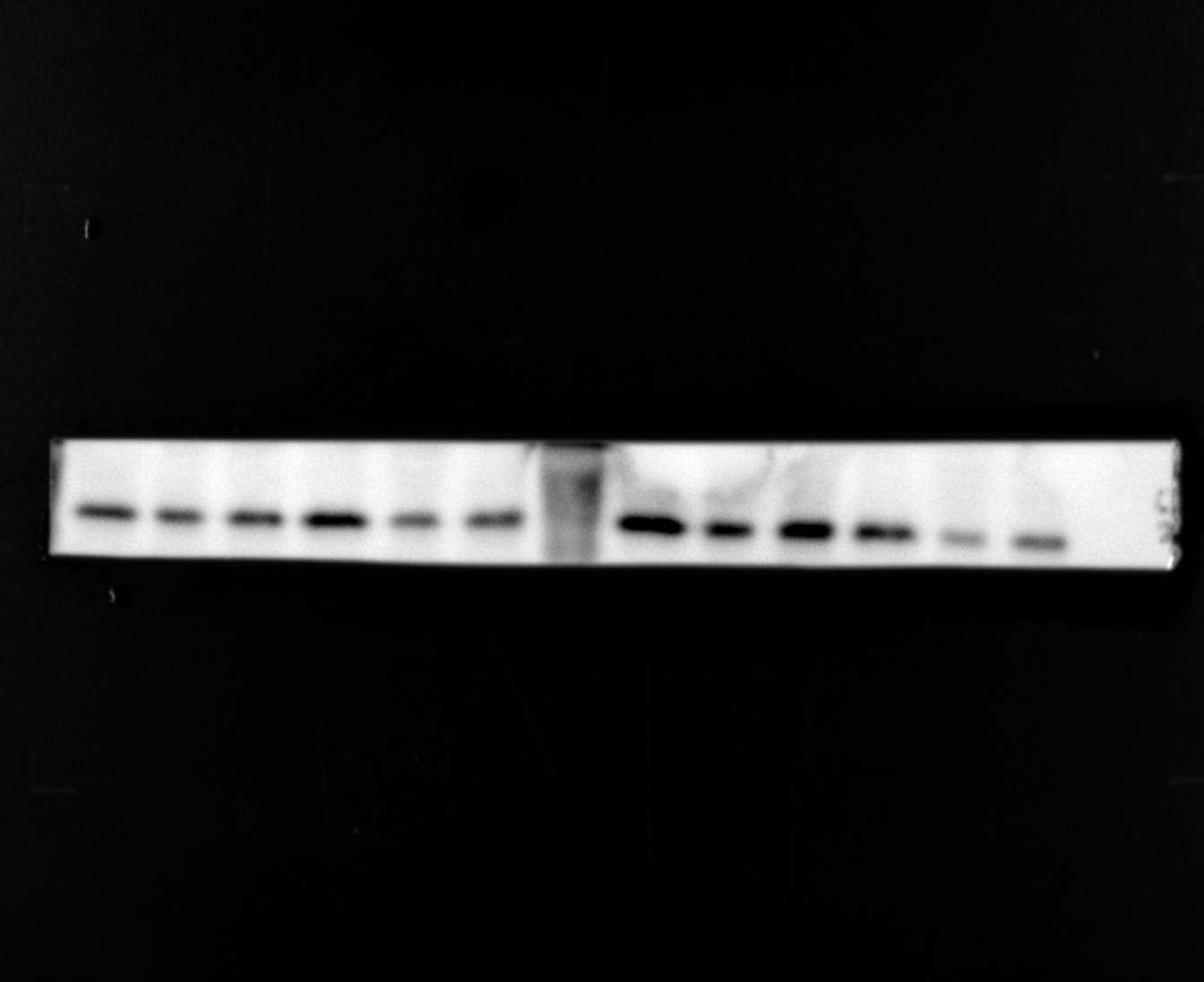

Supplement: Supplementary file 1 — Supplementary Material 1 [file 12876_2025_4196_MOESM1_ESM.zip › Supplementary Materials/Figure 5/Figure5-G/Figure5-Claudin1-2.Tif]

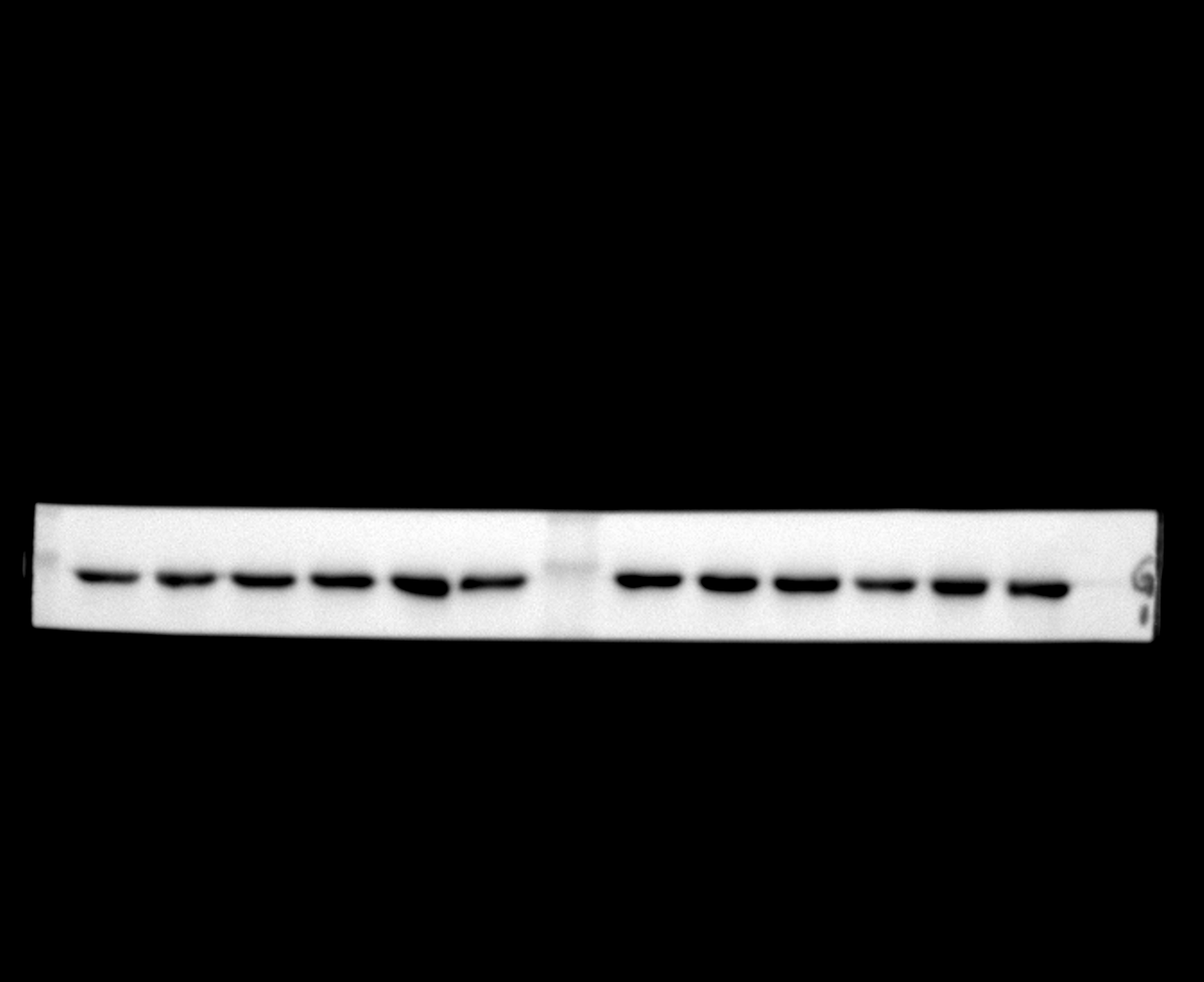

Supplement: Supplementary file 1 — Supplementary Material 1 [file 12876_2025_4196_MOESM1_ESM.zip › Supplementary Materials/Figure 5/Figure5-G/Figure5-GAPDH-1.Tif]

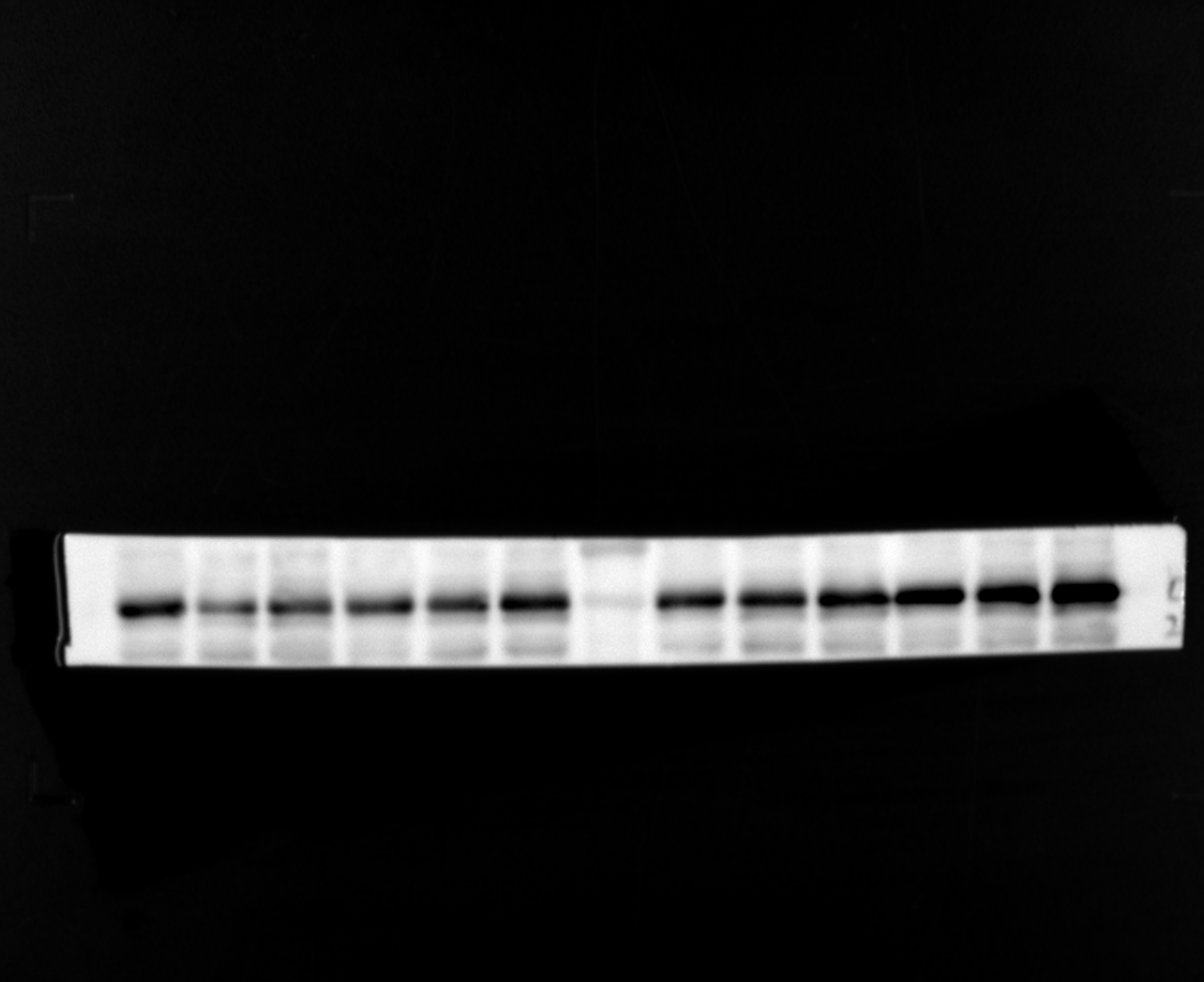

Supplement: Supplementary file 1 — Supplementary Material 1 [file 12876_2025_4196_MOESM1_ESM.zip › Supplementary Materials/Figure 5/Figure5-G/Figure5-Occludin-2.Tif]

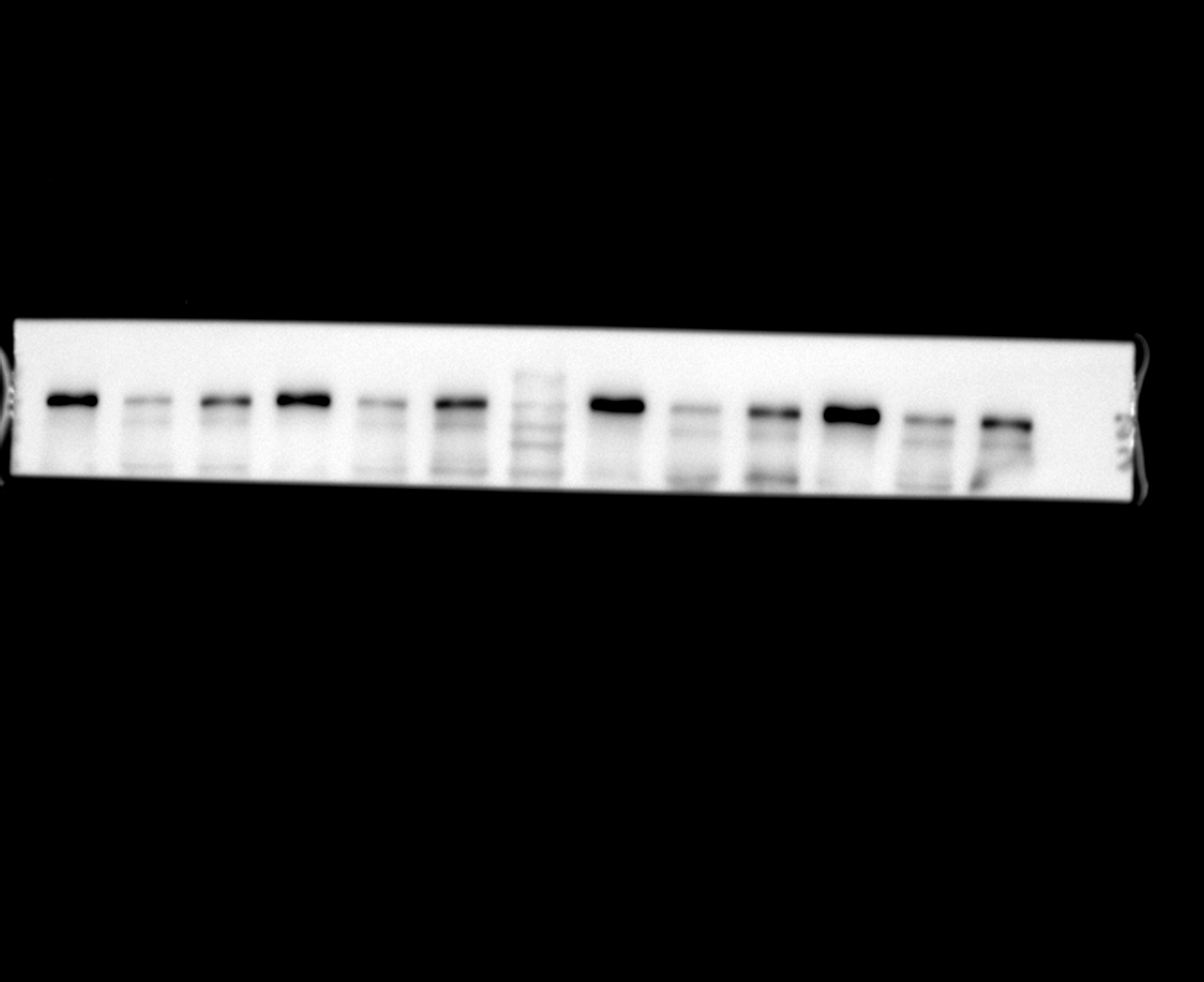

Supplement: Supplementary file 1 — Supplementary Material 1 [file 12876_2025_4196_MOESM1_ESM.zip › Supplementary Materials/Figure 5/Figure5-G/Figure5-ZO-1-2.Tif]

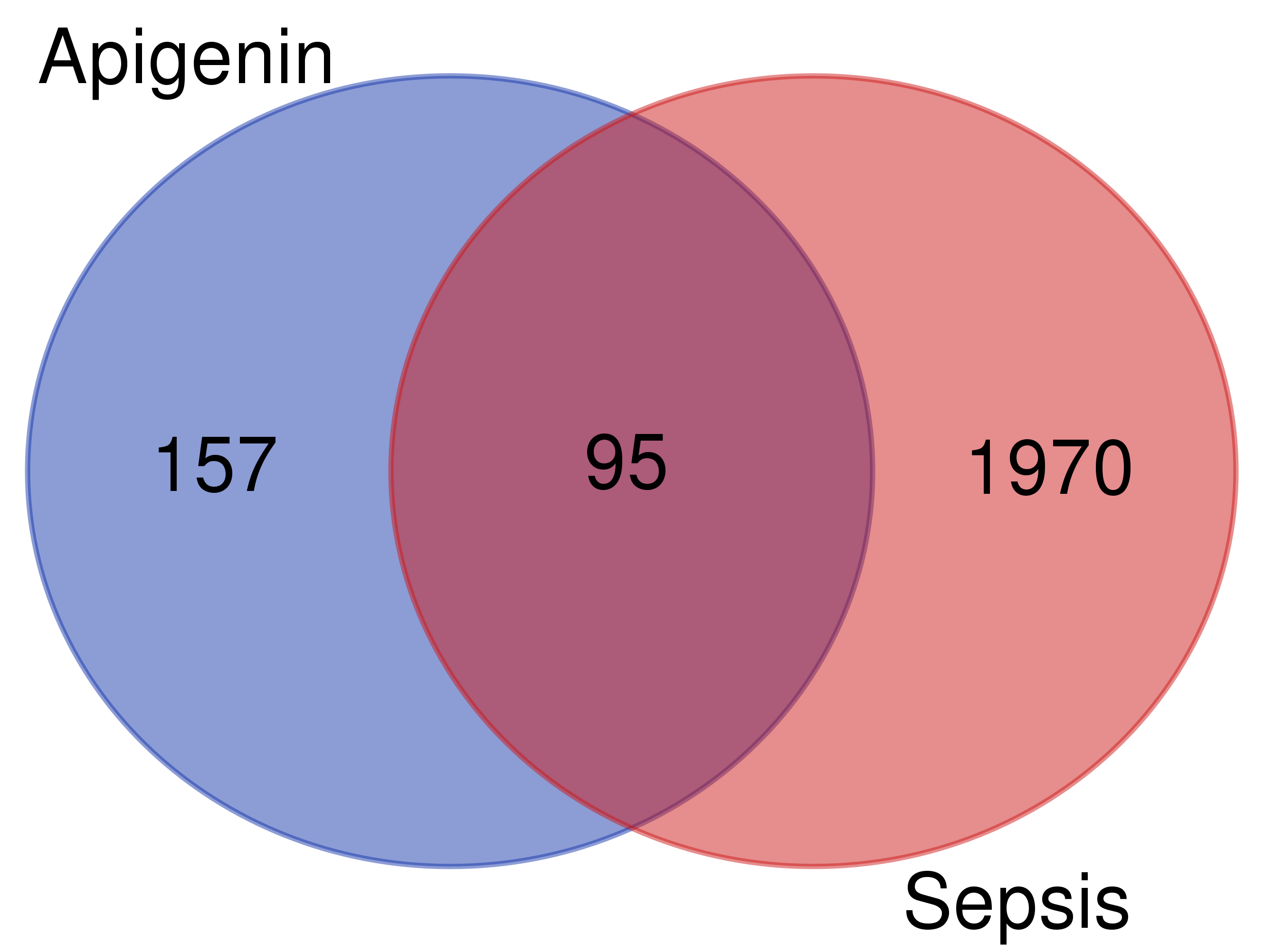

Supplement: Supplementary file 1 — Supplementary Material 1 [file 12876_2025_4196_MOESM1_ESM.zip › Supplementary Materials/Figure 6/Figure6-A.png]

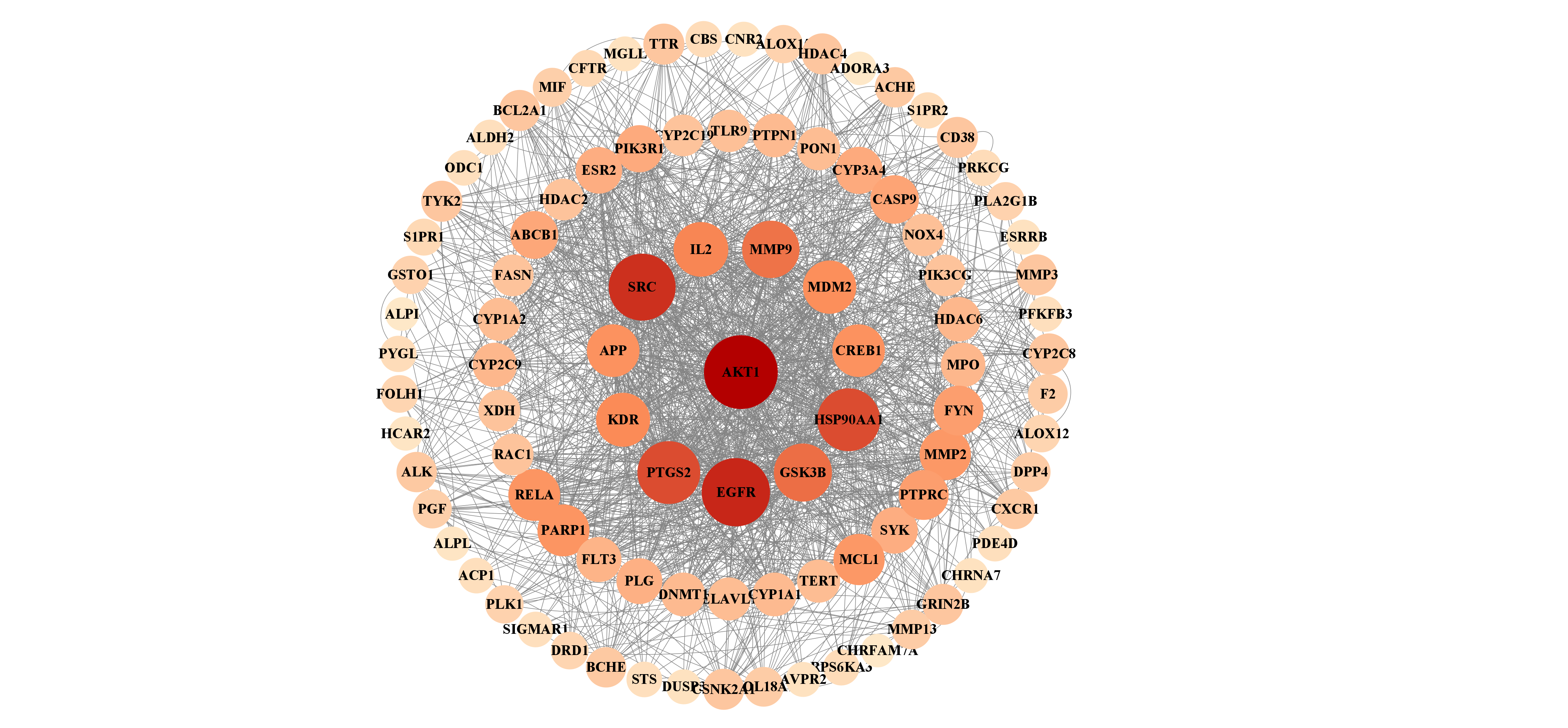

Supplement: Supplementary file 1 — Supplementary Material 1 [file 12876_2025_4196_MOESM1_ESM.zip › Supplementary Materials/Figure 6/Figure6-B.png]

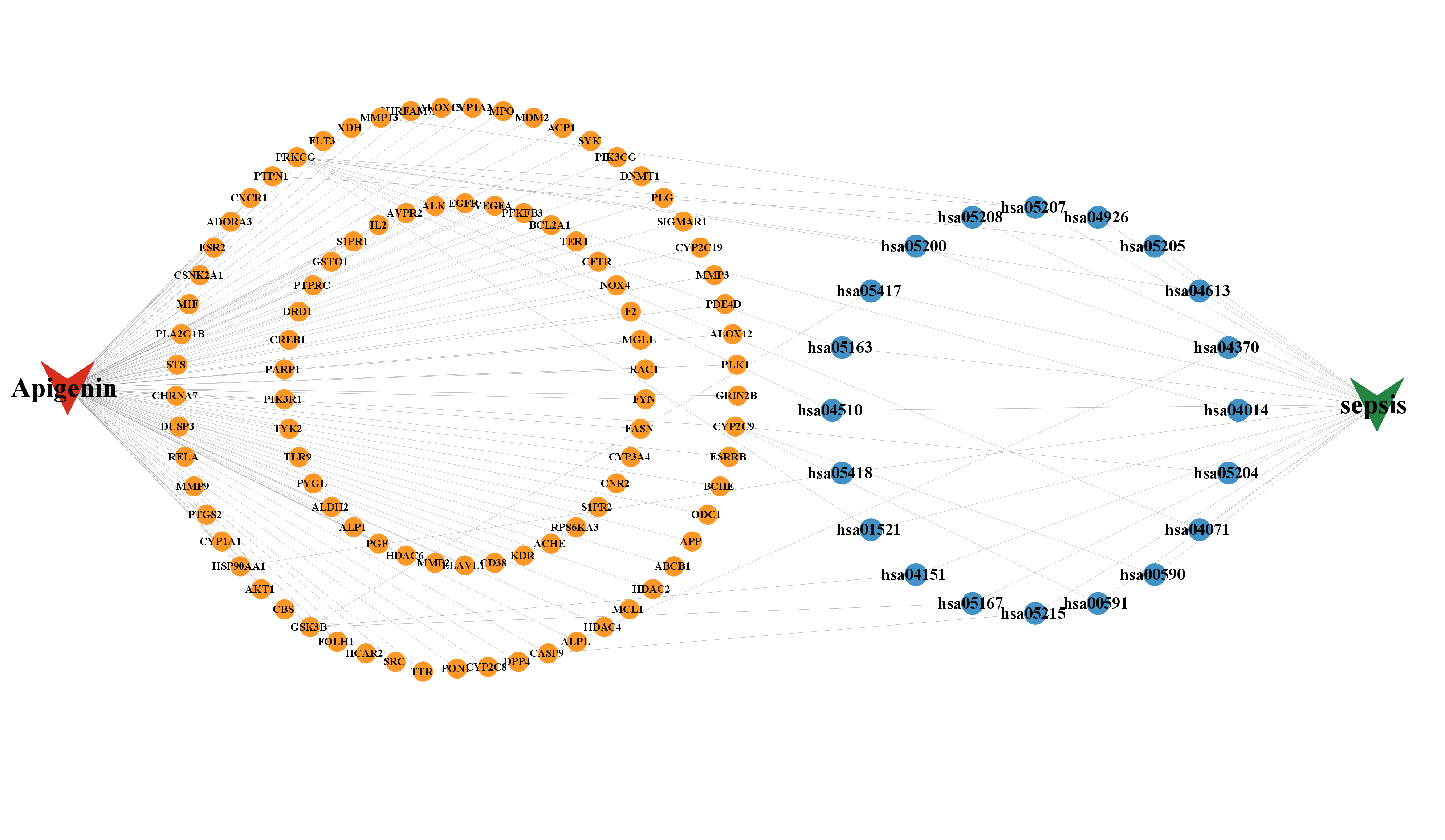

Supplement: Supplementary file 1 — Supplementary Material 1 [file 12876_2025_4196_MOESM1_ESM.zip › Supplementary Materials/Figure 6/Figure6-C.png]

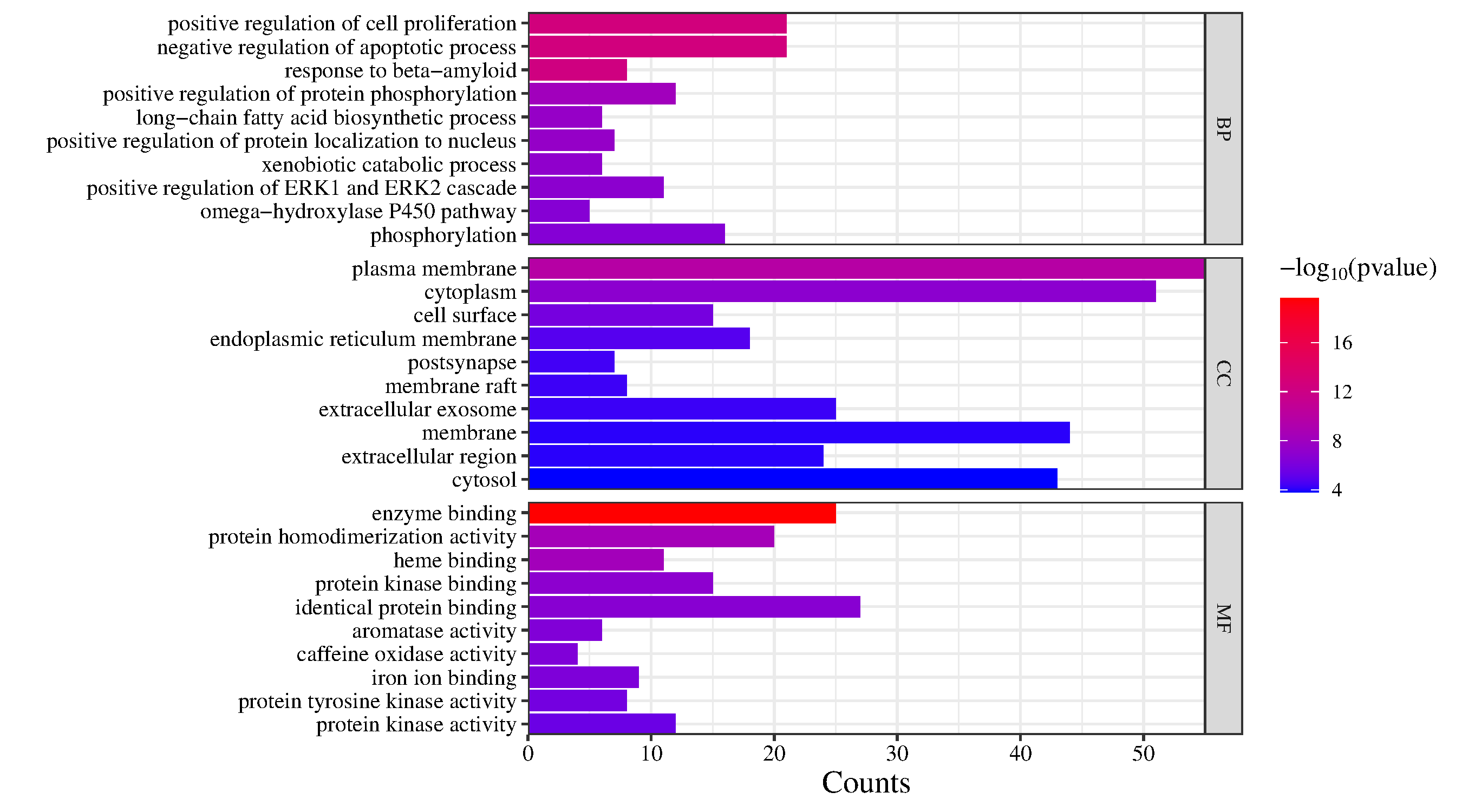

Supplement: Supplementary file 1 — Supplementary Material 1 [file 12876_2025_4196_MOESM1_ESM.zip › Supplementary Materials/Figure 7/Figure7-A.png]

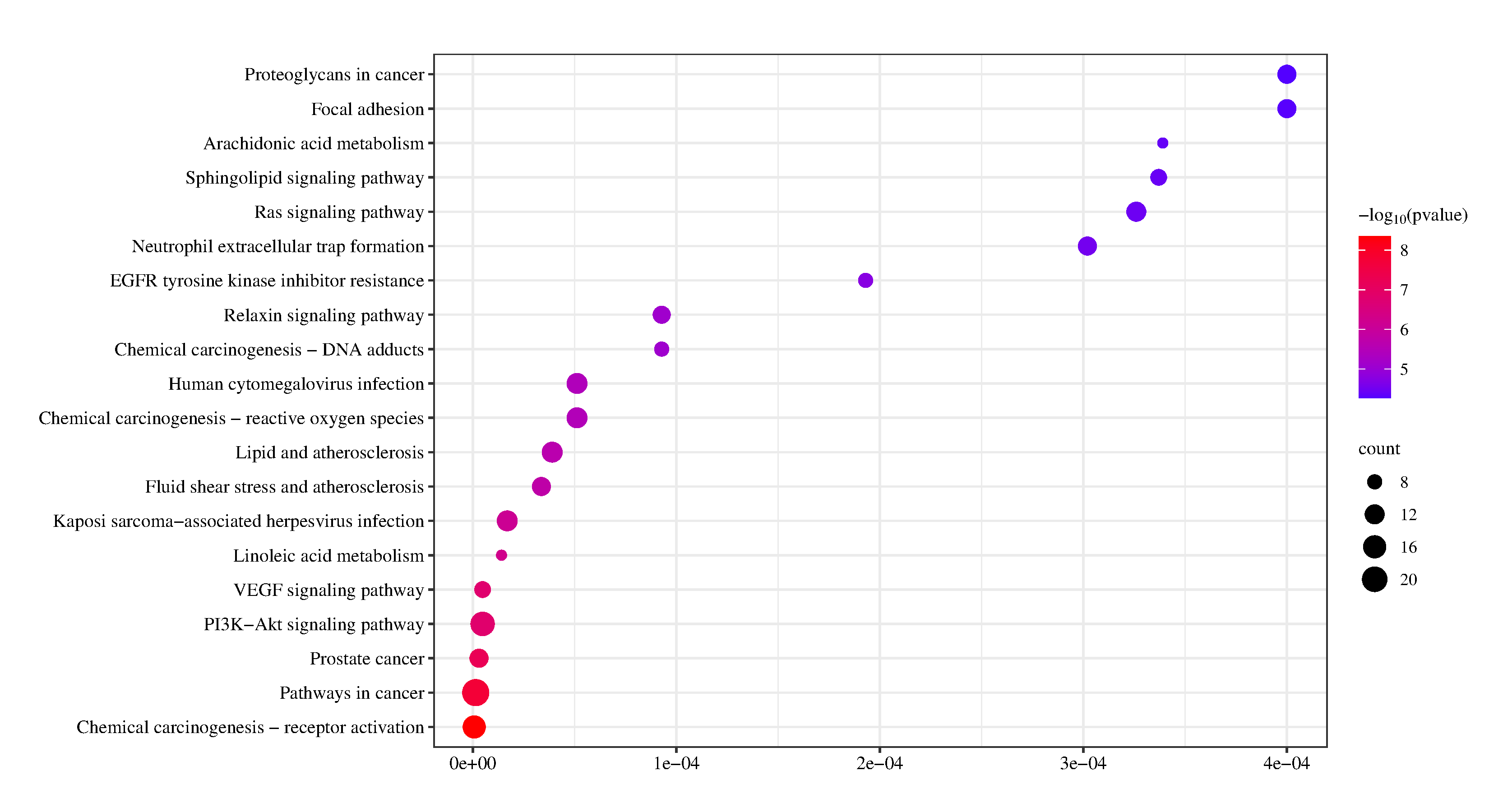

Supplement: Supplementary file 1 — Supplementary Material 1 [file 12876_2025_4196_MOESM1_ESM.zip › Supplementary Materials/Figure 7/Figure7-B.png]

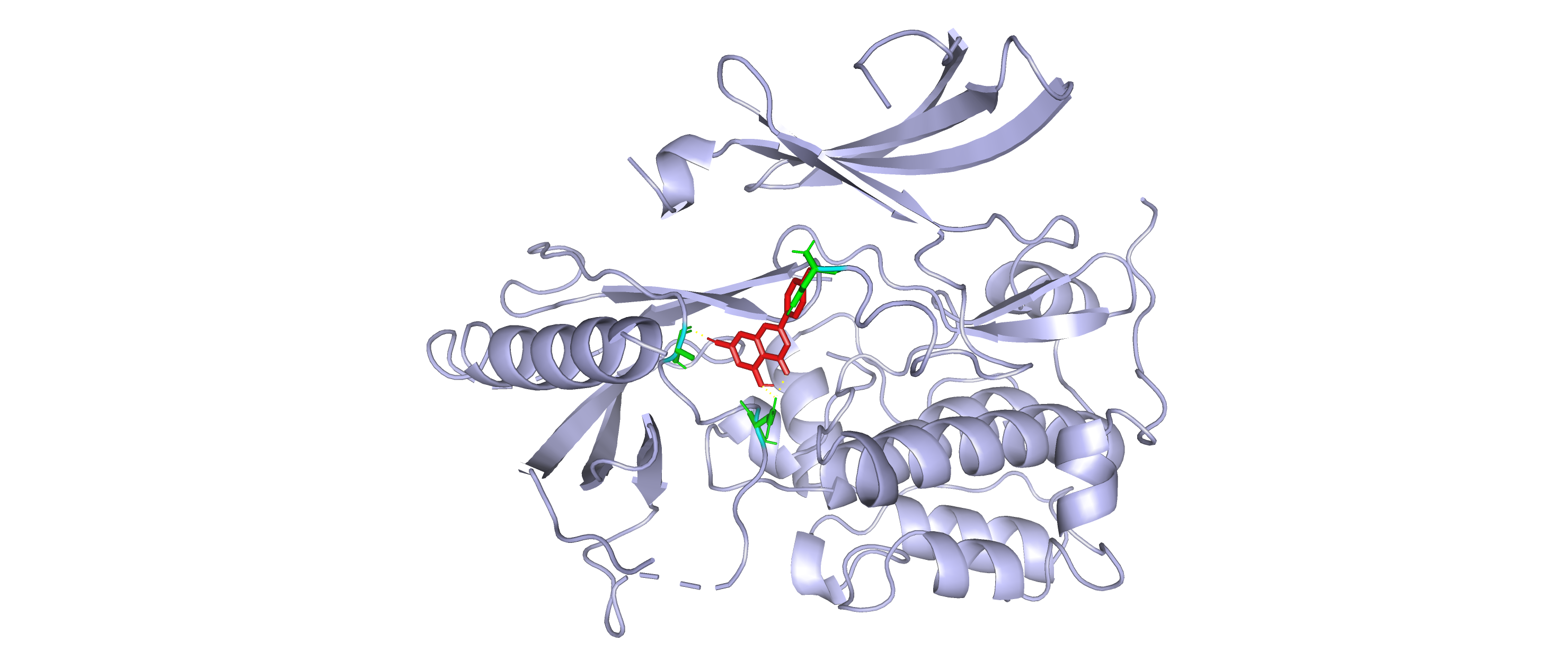

Supplement: Supplementary file 1 — Supplementary Material 1 [file 12876_2025_4196_MOESM1_ESM.zip › Supplementary Materials/Figure 8/Figure8-AKT-API-A.png]

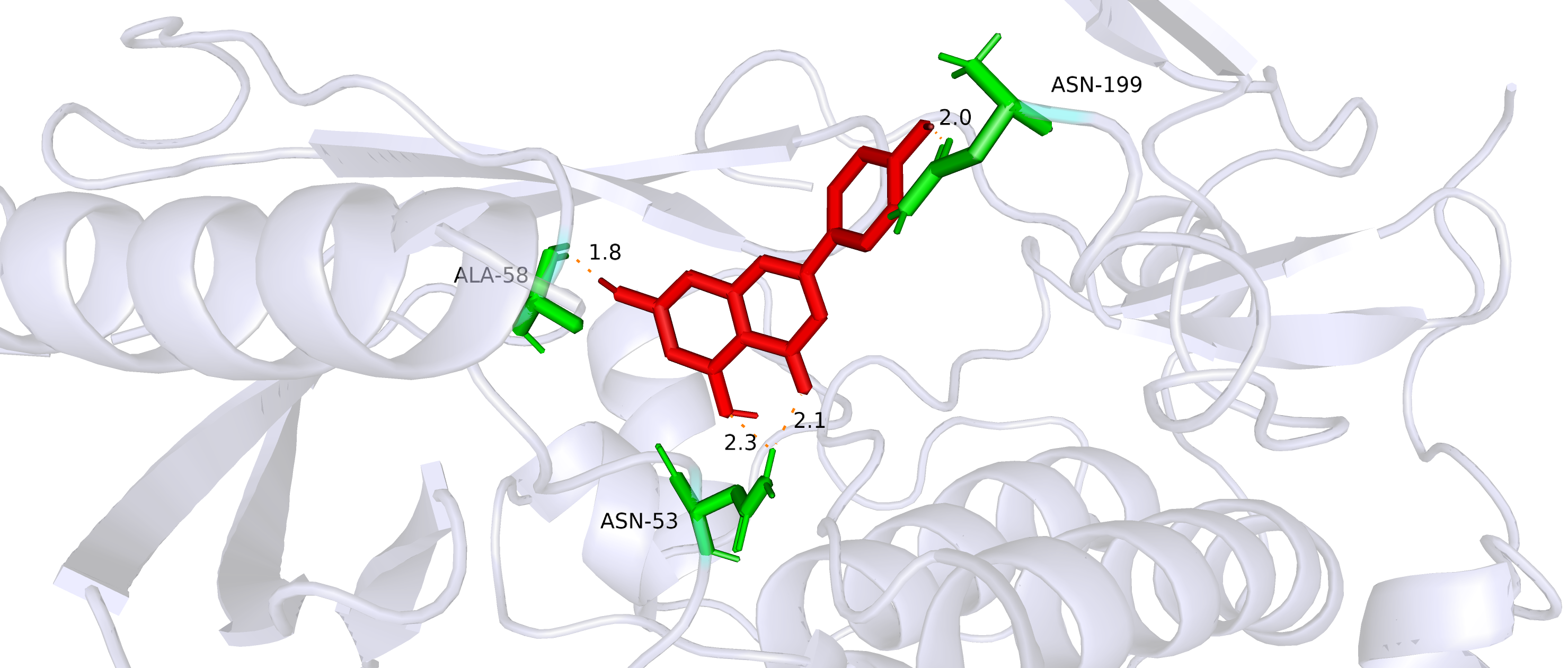

Supplement: Supplementary file 1 — Supplementary Material 1 [file 12876_2025_4196_MOESM1_ESM.zip › Supplementary Materials/Figure 8/Figure8-AKT-API-B.png]

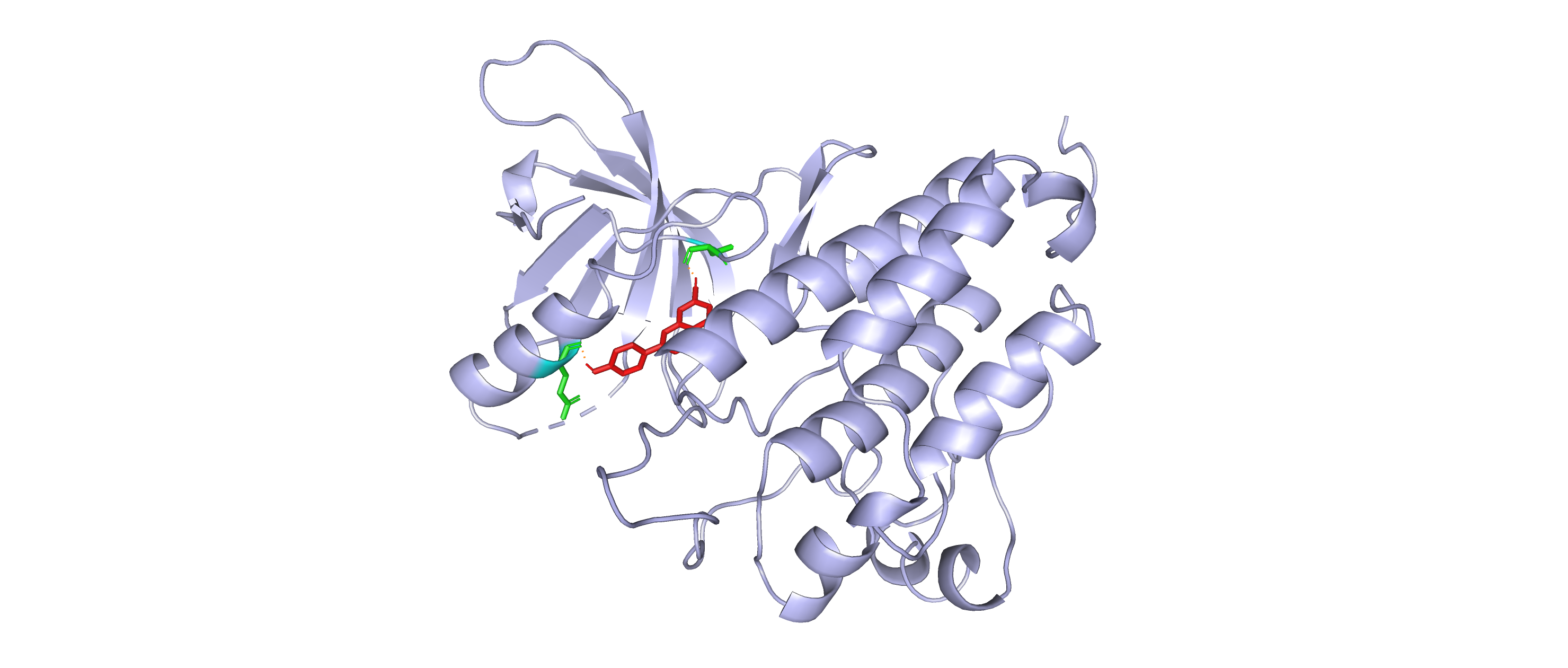

Supplement: Supplementary file 1 — Supplementary Material 1 [file 12876_2025_4196_MOESM1_ESM.zip › Supplementary Materials/Figure 8/Figure8-EGFR-API-A.png]

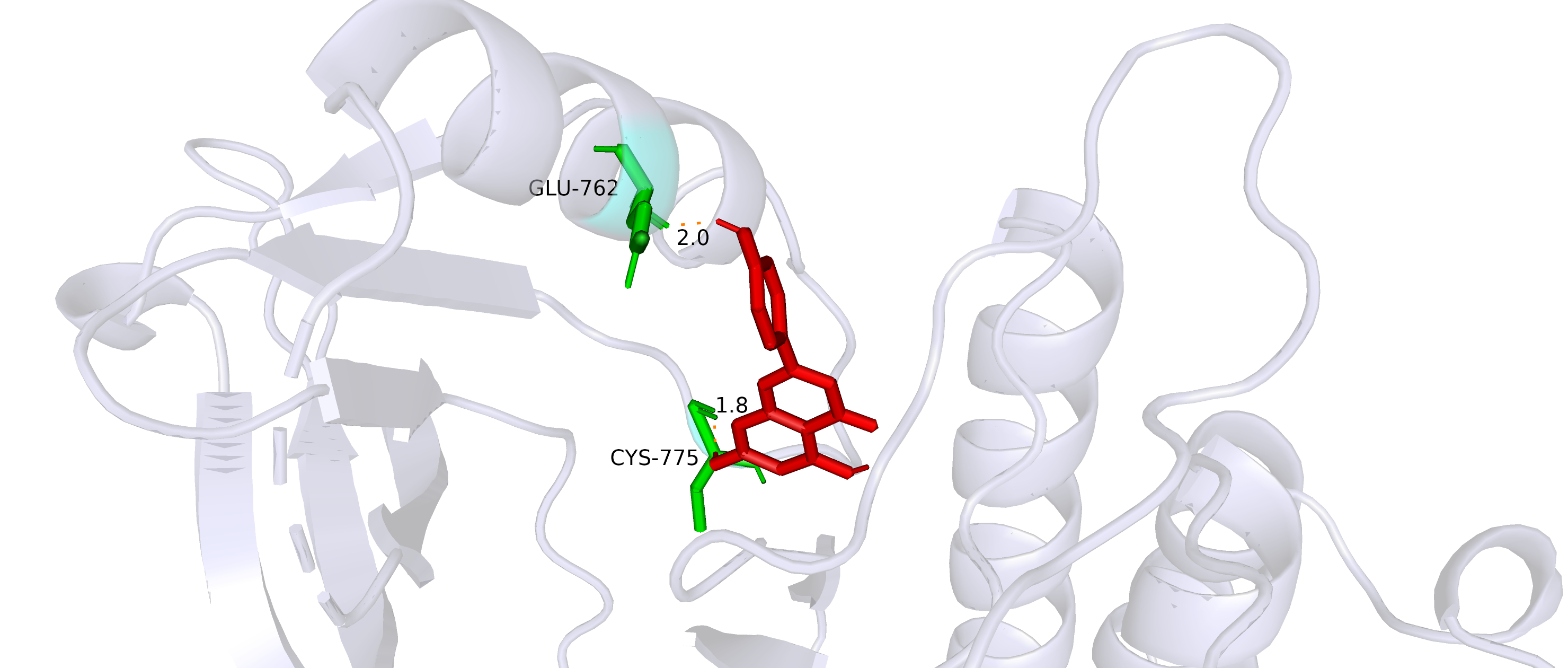

Supplement: Supplementary file 1 — Supplementary Material 1 [file 12876_2025_4196_MOESM1_ESM.zip › Supplementary Materials/Figure 8/Figure8-EGFR-API-B.png]

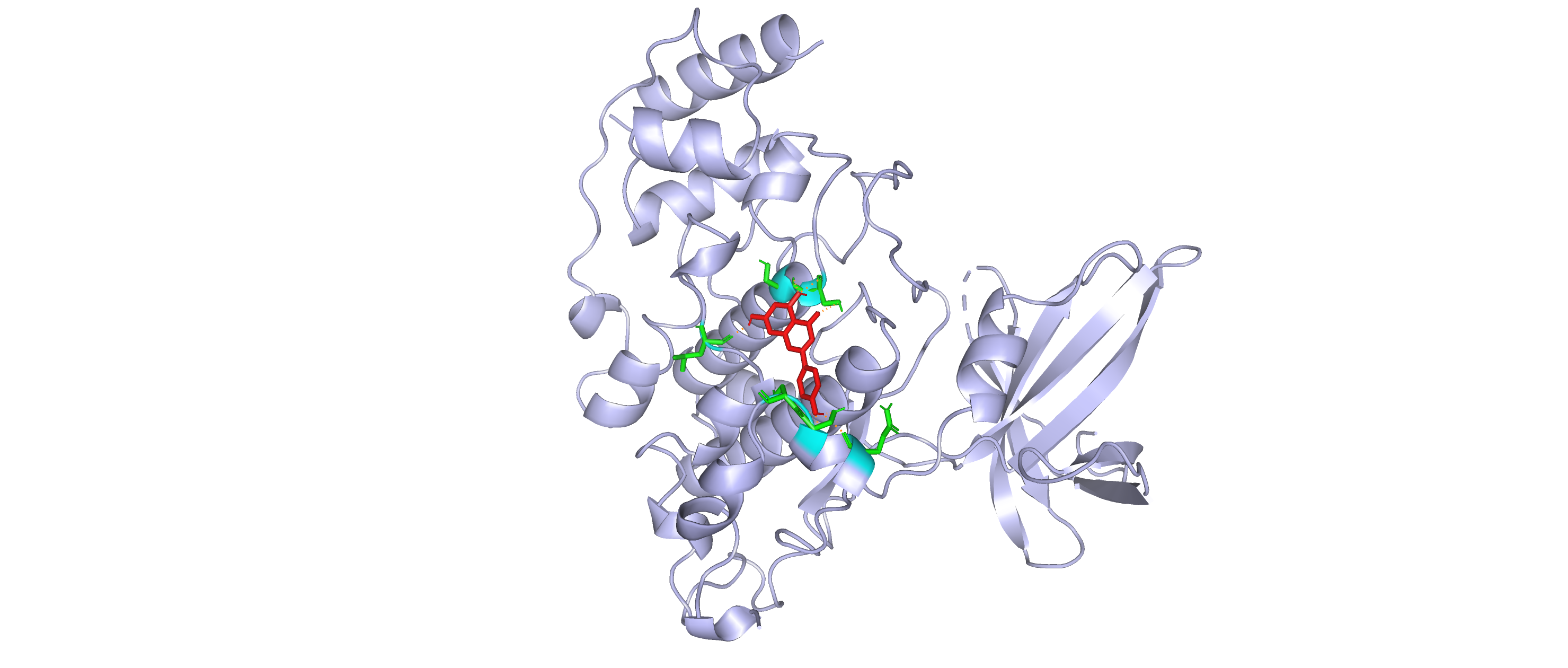

Supplement: Supplementary file 1 — Supplementary Material 1 [file 12876_2025_4196_MOESM1_ESM.zip › Supplementary Materials/Figure 8/Figure8-GSK3B_API_A.png]

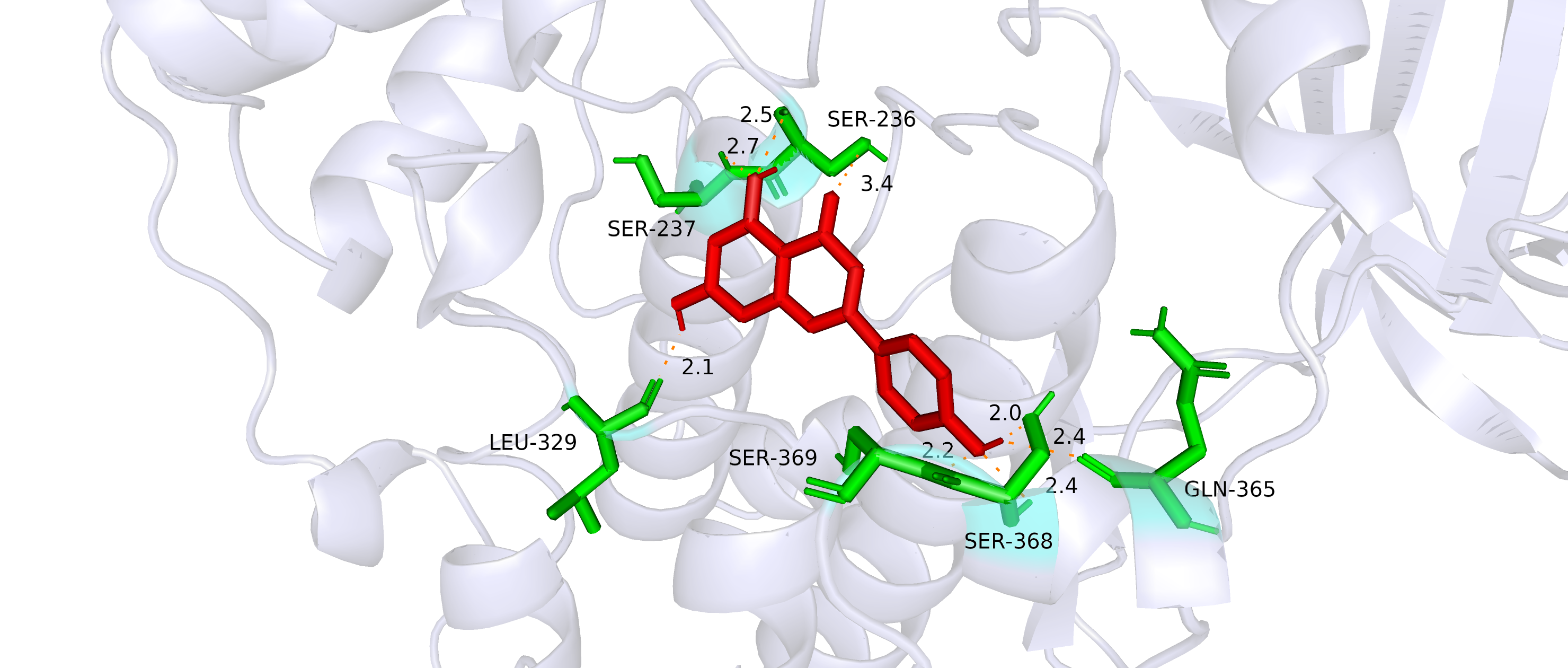

Supplement: Supplementary file 1 — Supplementary Material 1 [file 12876_2025_4196_MOESM1_ESM.zip › Supplementary Materials/Figure 8/Figure8-GSK3B_API_B.png]

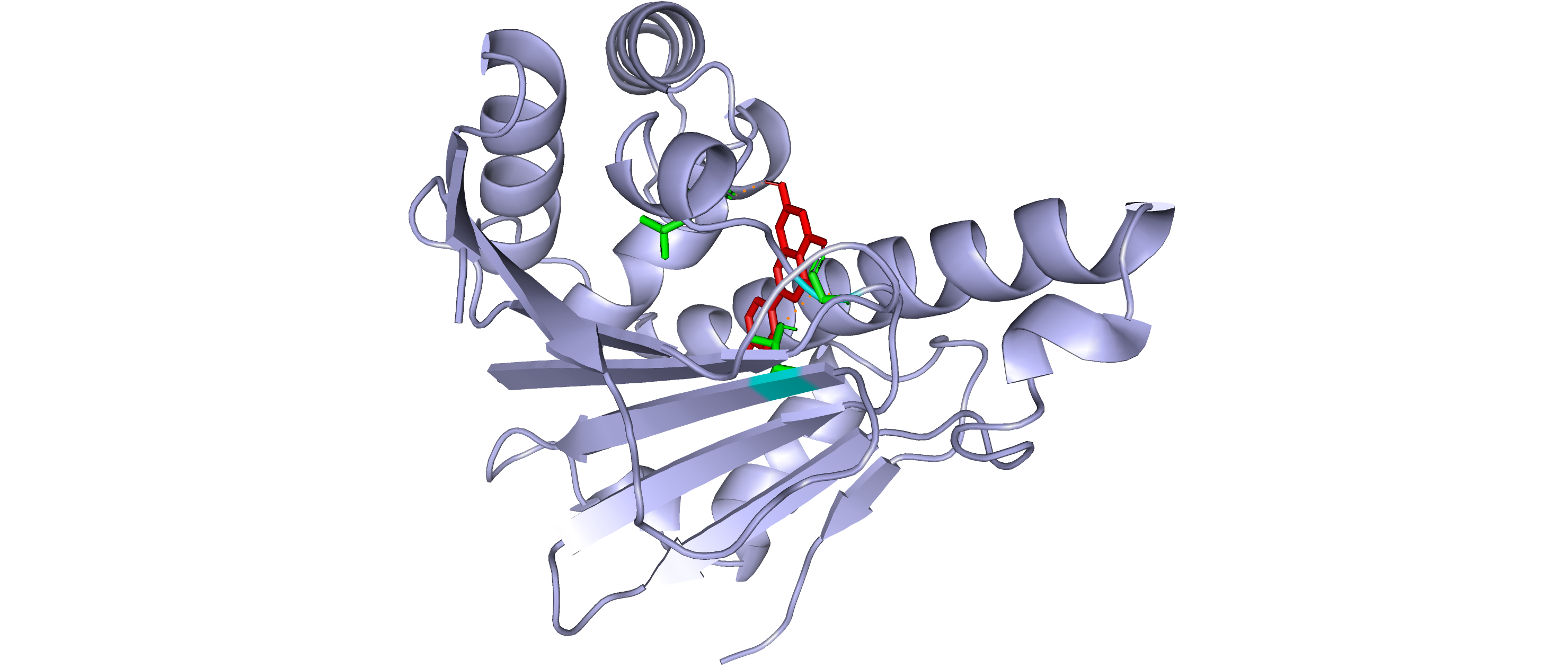

Supplement: Supplementary file 1 — Supplementary Material 1 [file 12876_2025_4196_MOESM1_ESM.zip › Supplementary Materials/Figure 8/Figure8-HAP90AA1_API_A.png]

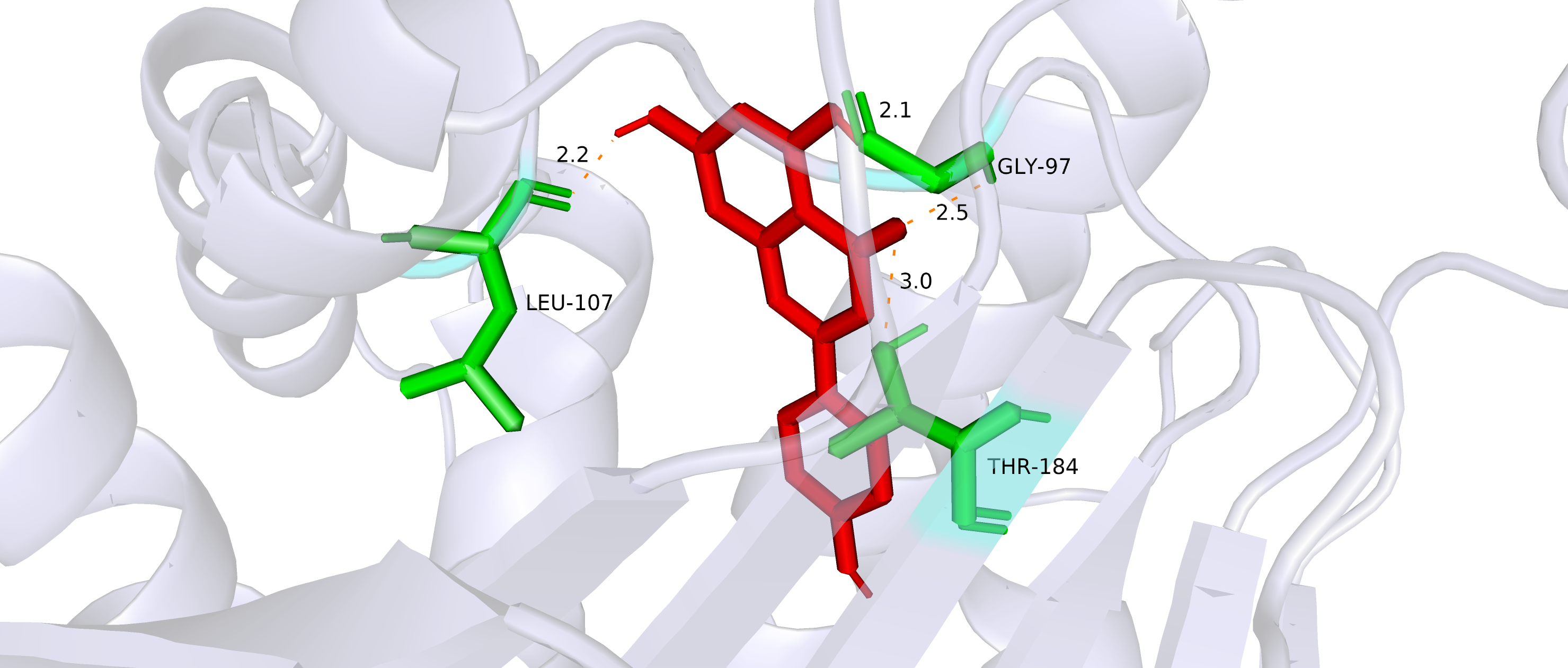

Supplement: Supplementary file 1 — Supplementary Material 1 [file 12876_2025_4196_MOESM1_ESM.zip › Supplementary Materials/Figure 8/Figure8-HAP90AA1_API_B.png]

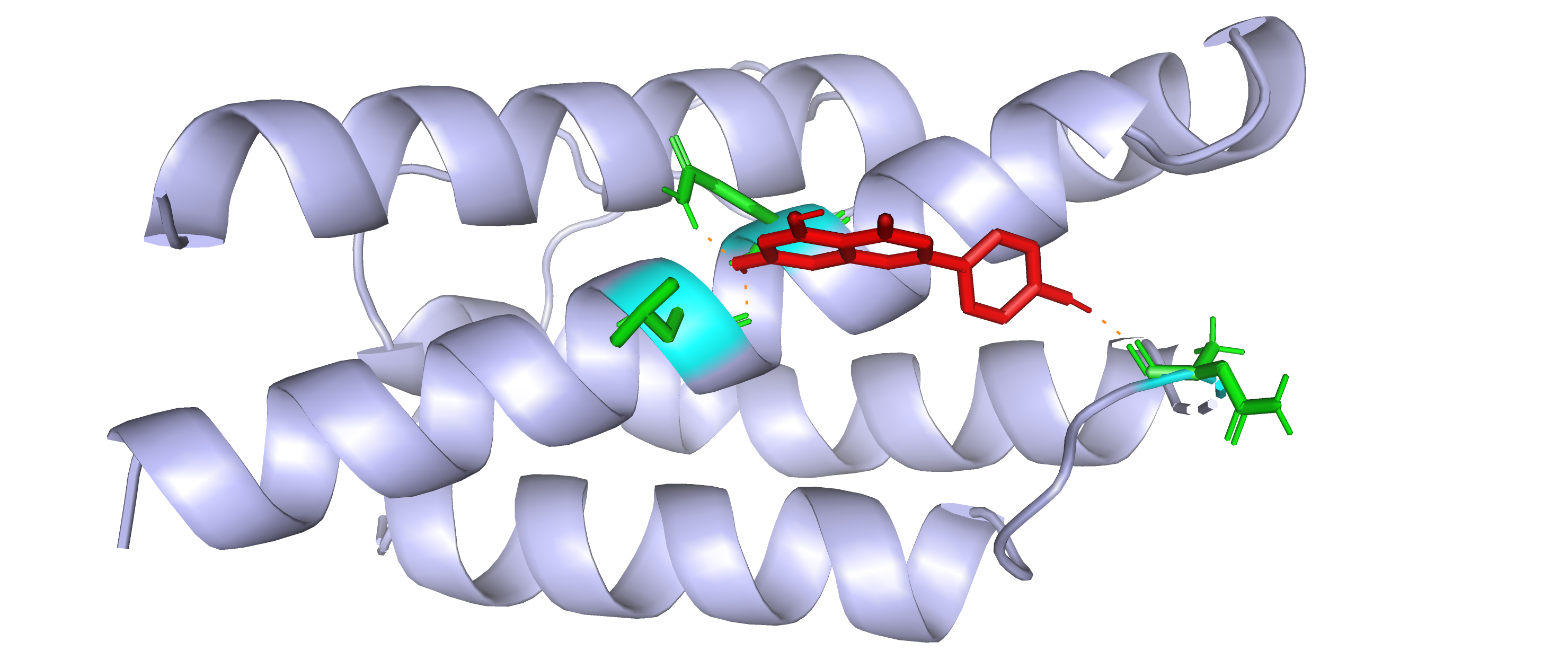

Supplement: Supplementary file 1 — Supplementary Material 1 [file 12876_2025_4196_MOESM1_ESM.zip › Supplementary Materials/Figure 8/Figure8-IL2_API_A.png]

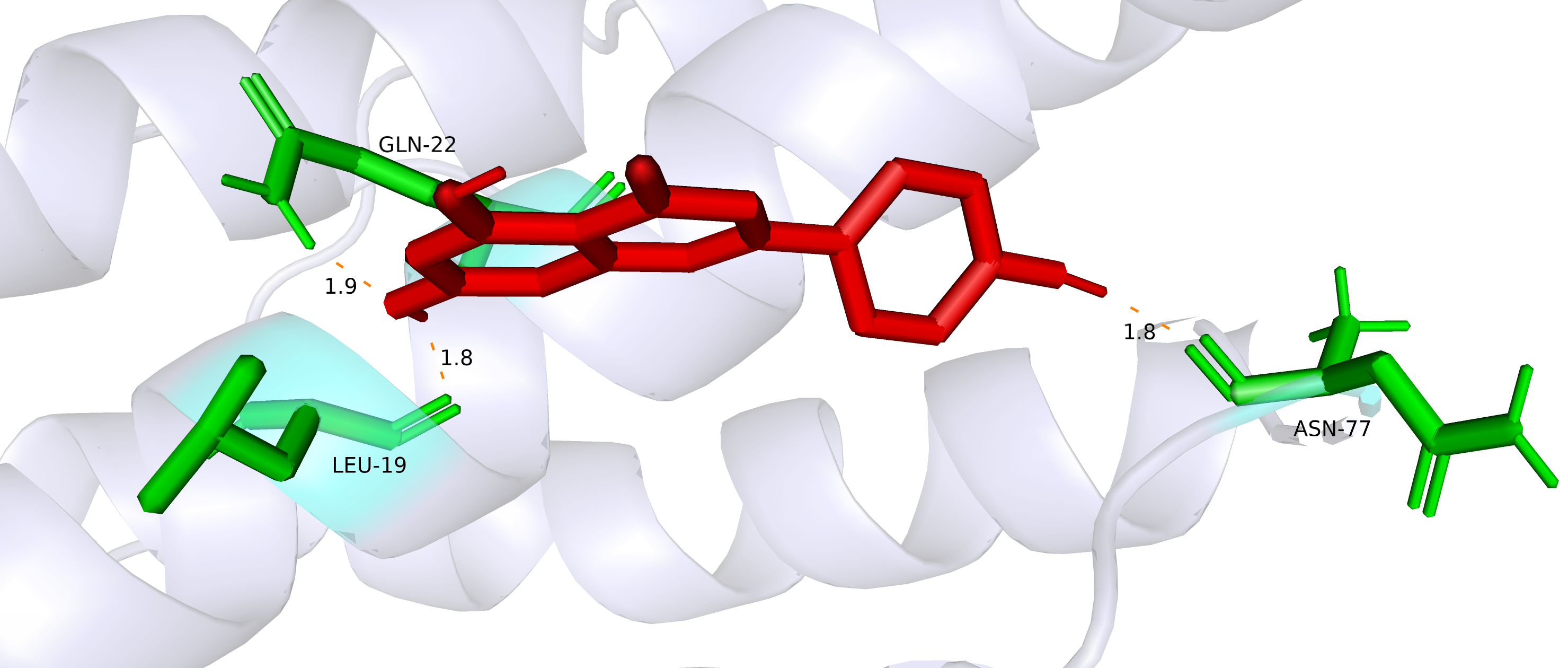

Supplement: Supplementary file 1 — Supplementary Material 1 [file 12876_2025_4196_MOESM1_ESM.zip › Supplementary Materials/Figure 8/Figure8-IL2_API_B.png]

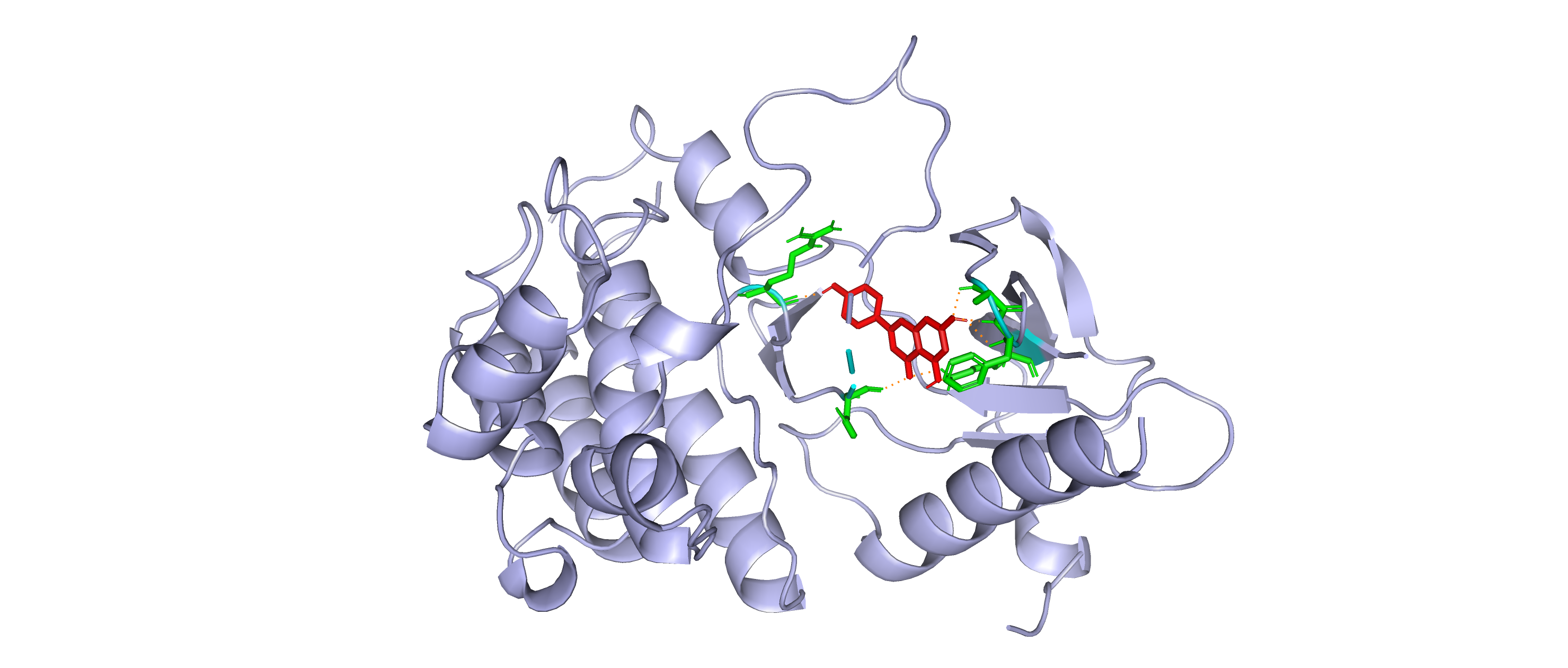

Supplement: Supplementary file 1 — Supplementary Material 1 [file 12876_2025_4196_MOESM1_ESM.zip › Supplementary Materials/Figure 8/Figure8-KDR_API_A.png]

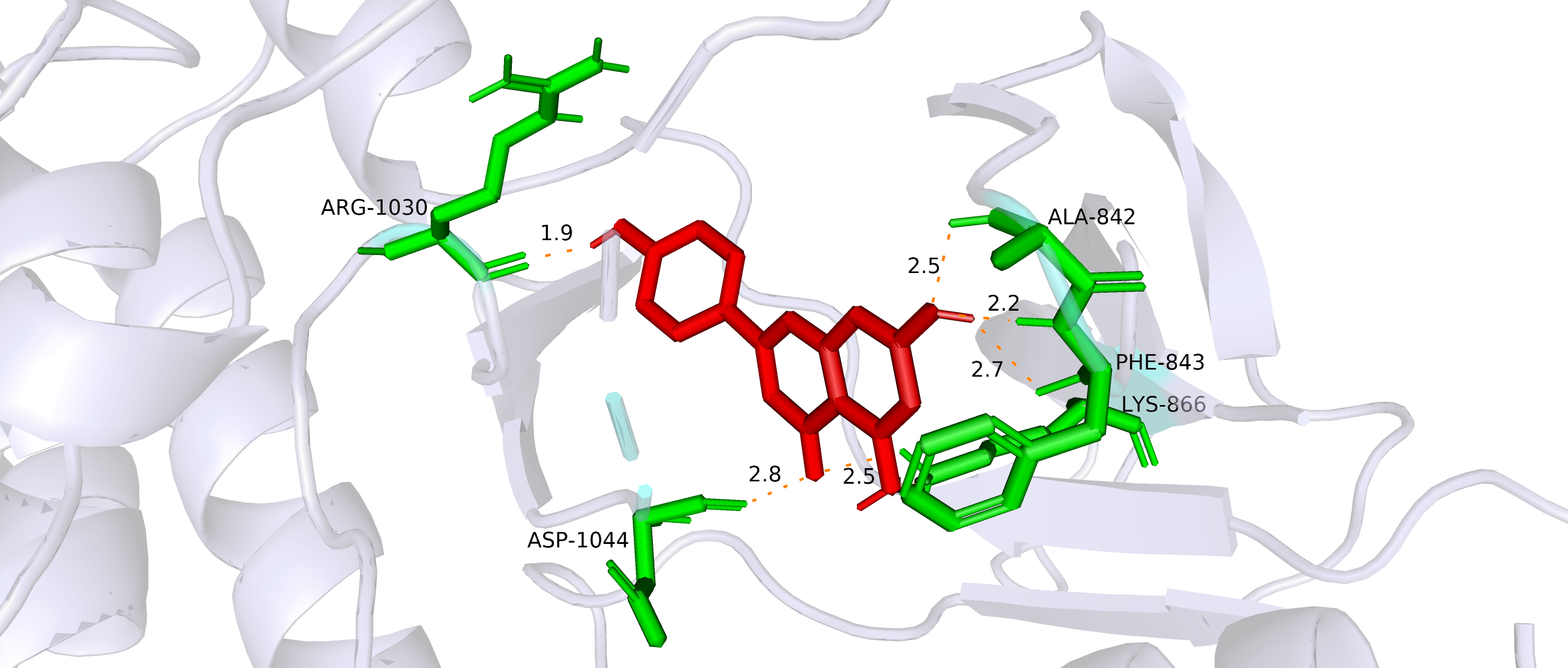

Supplement: Supplementary file 1 — Supplementary Material 1 [file 12876_2025_4196_MOESM1_ESM.zip › Supplementary Materials/Figure 8/Figure8-KDR_API_B.png]

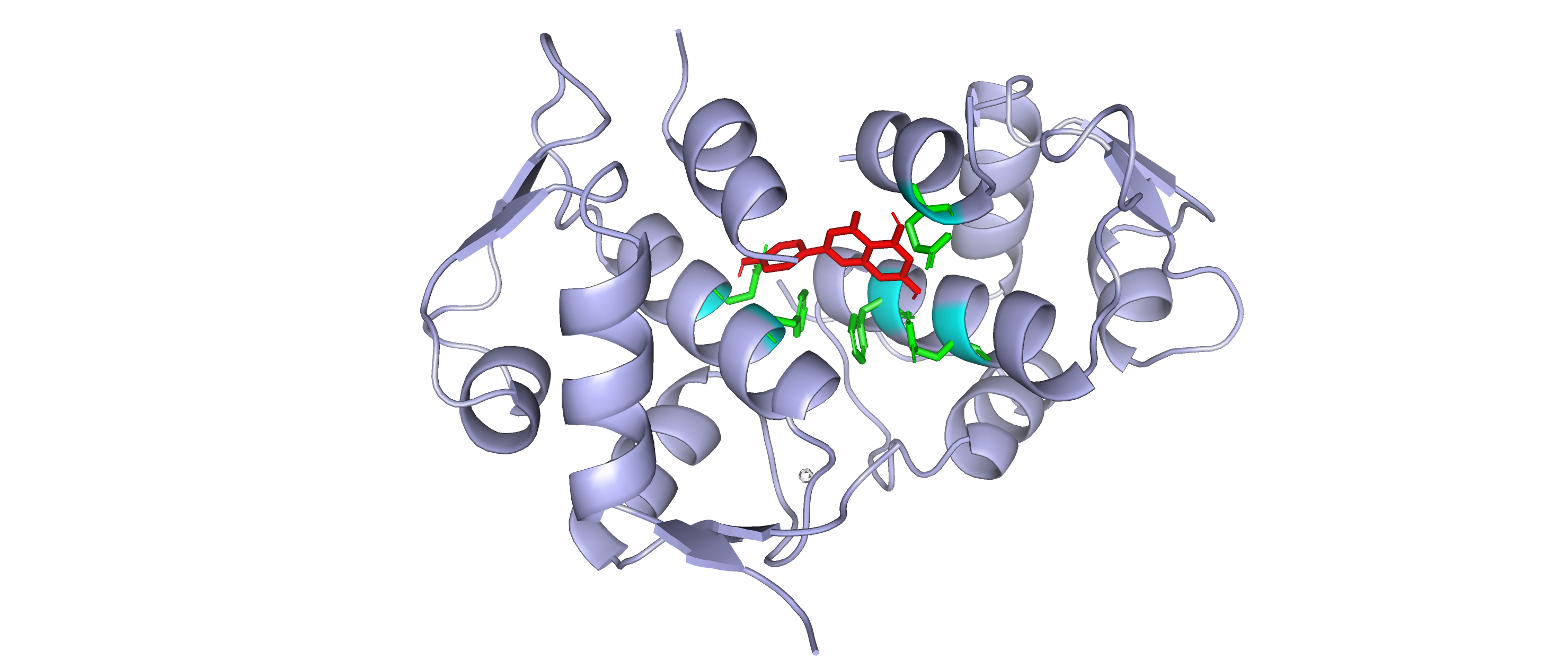

Supplement: Supplementary file 1 — Supplementary Material 1 [file 12876_2025_4196_MOESM1_ESM.zip › Supplementary Materials/Figure 8/Figure8-MDM2_API_A.png]

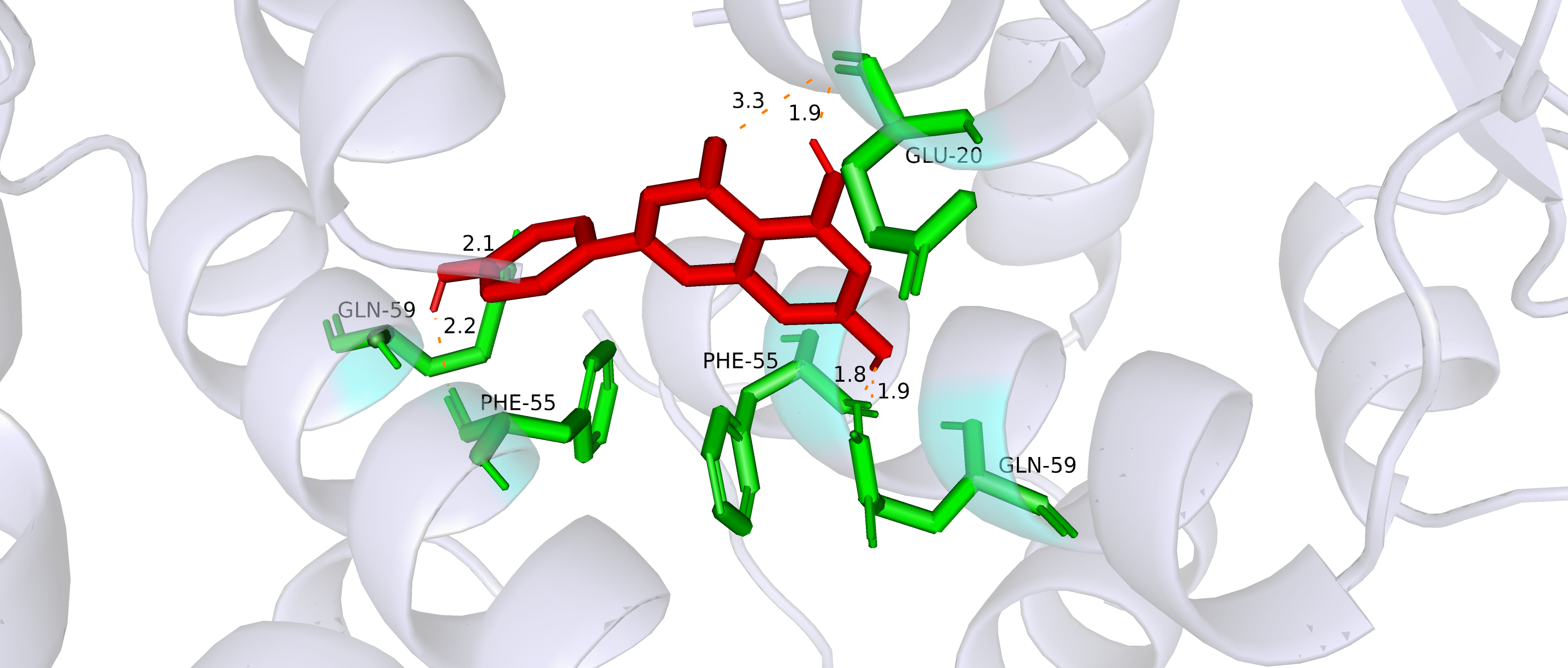

Supplement: Supplementary file 1 — Supplementary Material 1 [file 12876_2025_4196_MOESM1_ESM.zip › Supplementary Materials/Figure 8/Figure8-MDM2_API_B.png]

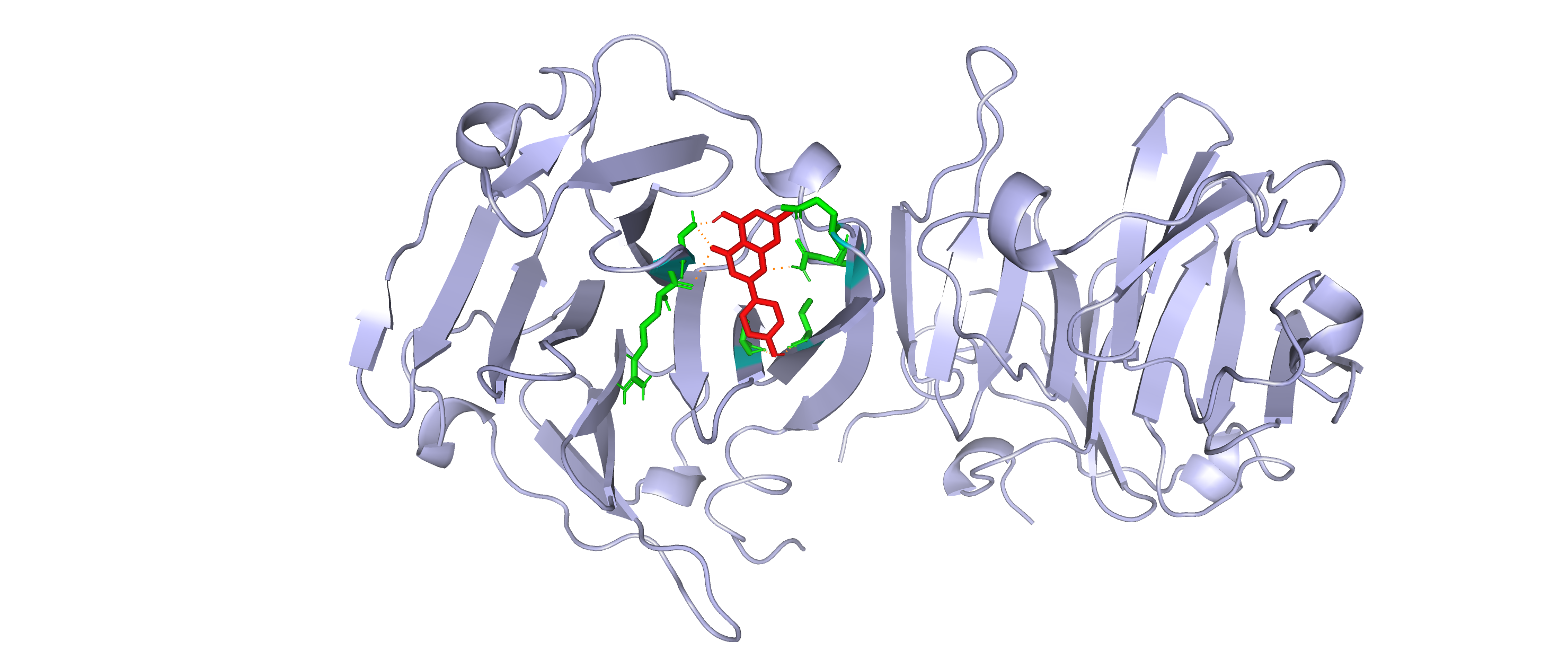

Supplement: Supplementary file 1 — Supplementary Material 1 [file 12876_2025_4196_MOESM1_ESM.zip › Supplementary Materials/Figure 8/Figure8-MMP9_API_A.png]

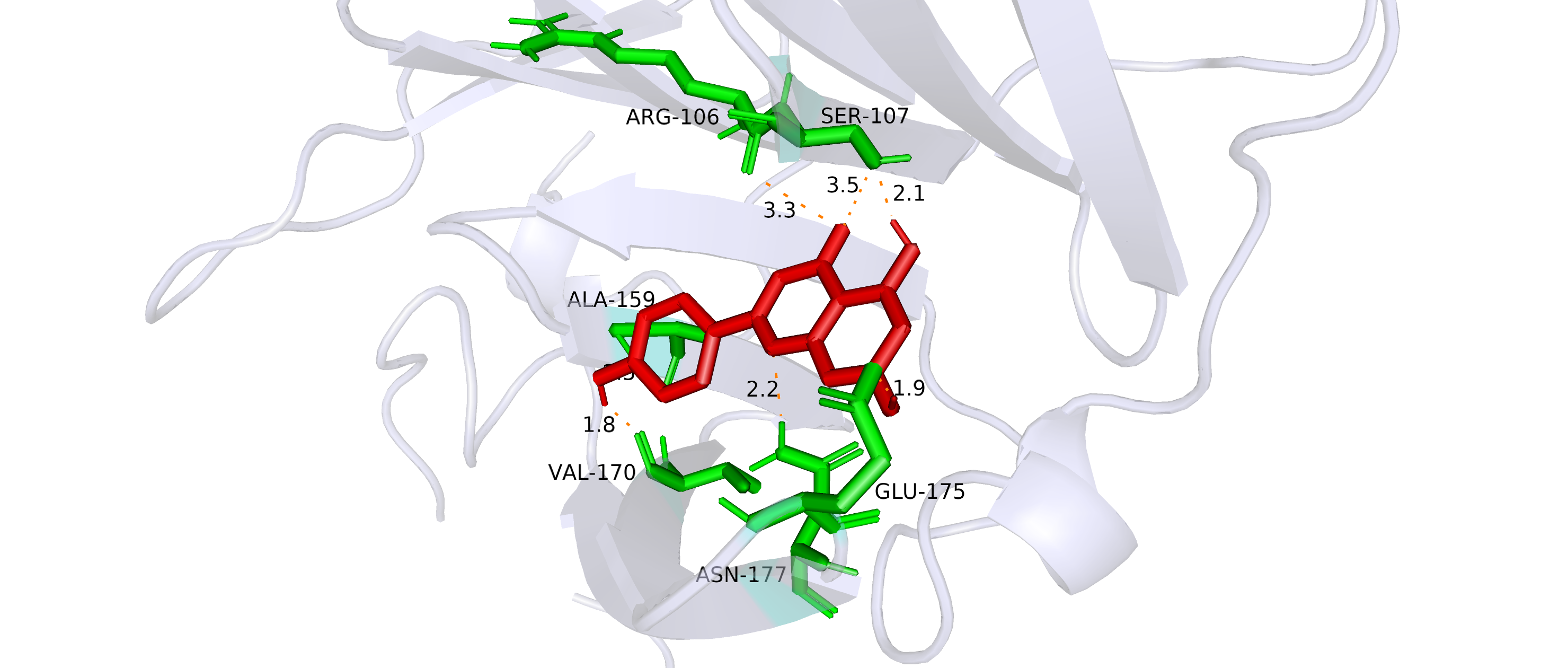

Supplement: Supplementary file 1 — Supplementary Material 1 [file 12876_2025_4196_MOESM1_ESM.zip › Supplementary Materials/Figure 8/Figure8-MMP9_API_B.png]

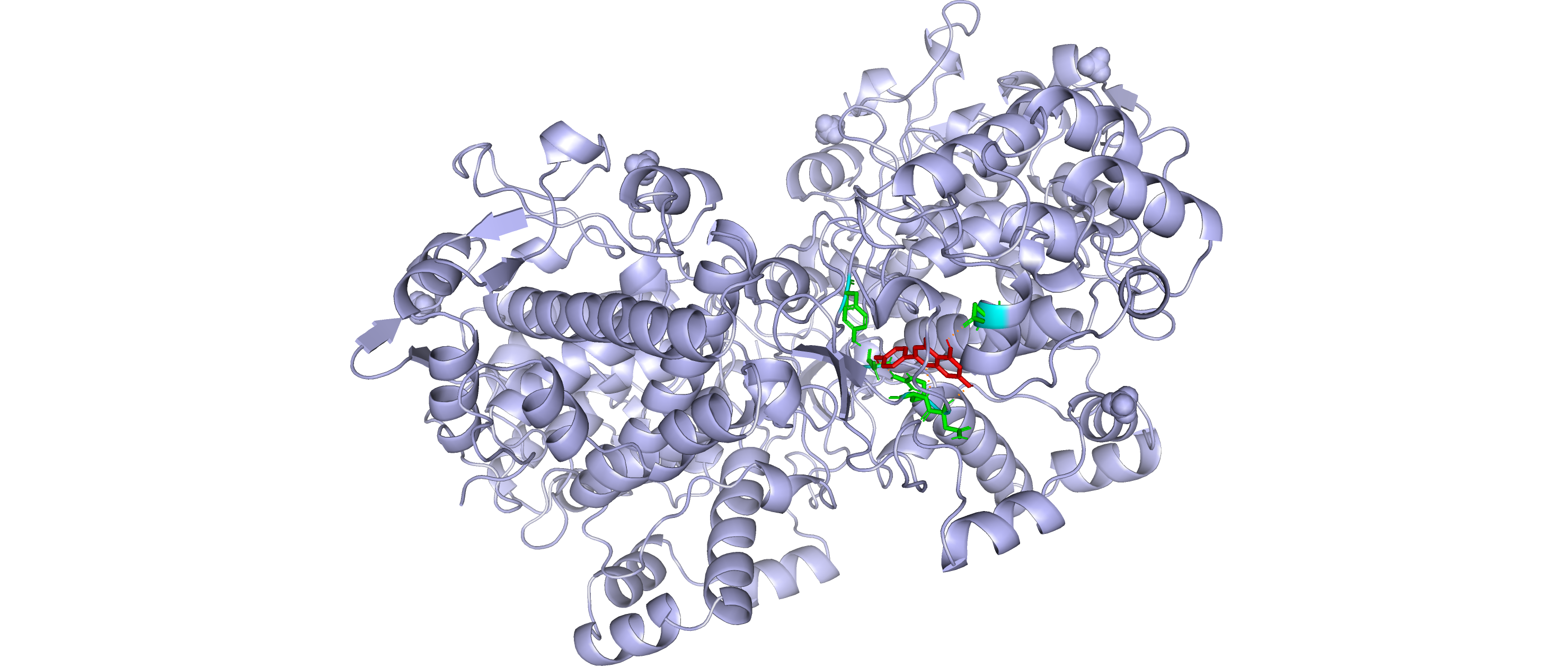

Supplement: Supplementary file 1 — Supplementary Material 1 [file 12876_2025_4196_MOESM1_ESM.zip › Supplementary Materials/Figure 8/Figure8-PTGS2_API_A.png]

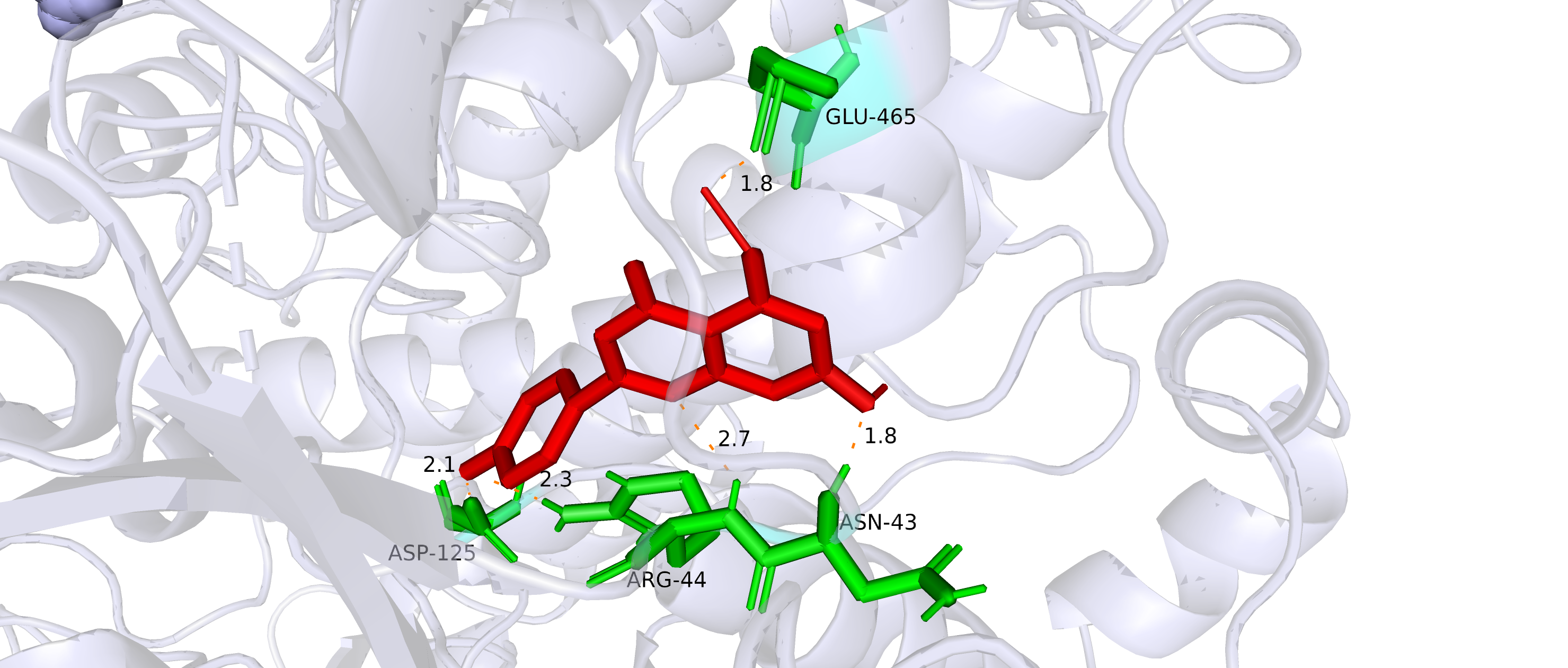

Supplement: Supplementary file 1 — Supplementary Material 1 [file 12876_2025_4196_MOESM1_ESM.zip › Supplementary Materials/Figure 8/Figure8-PTGS2_API_B.png]

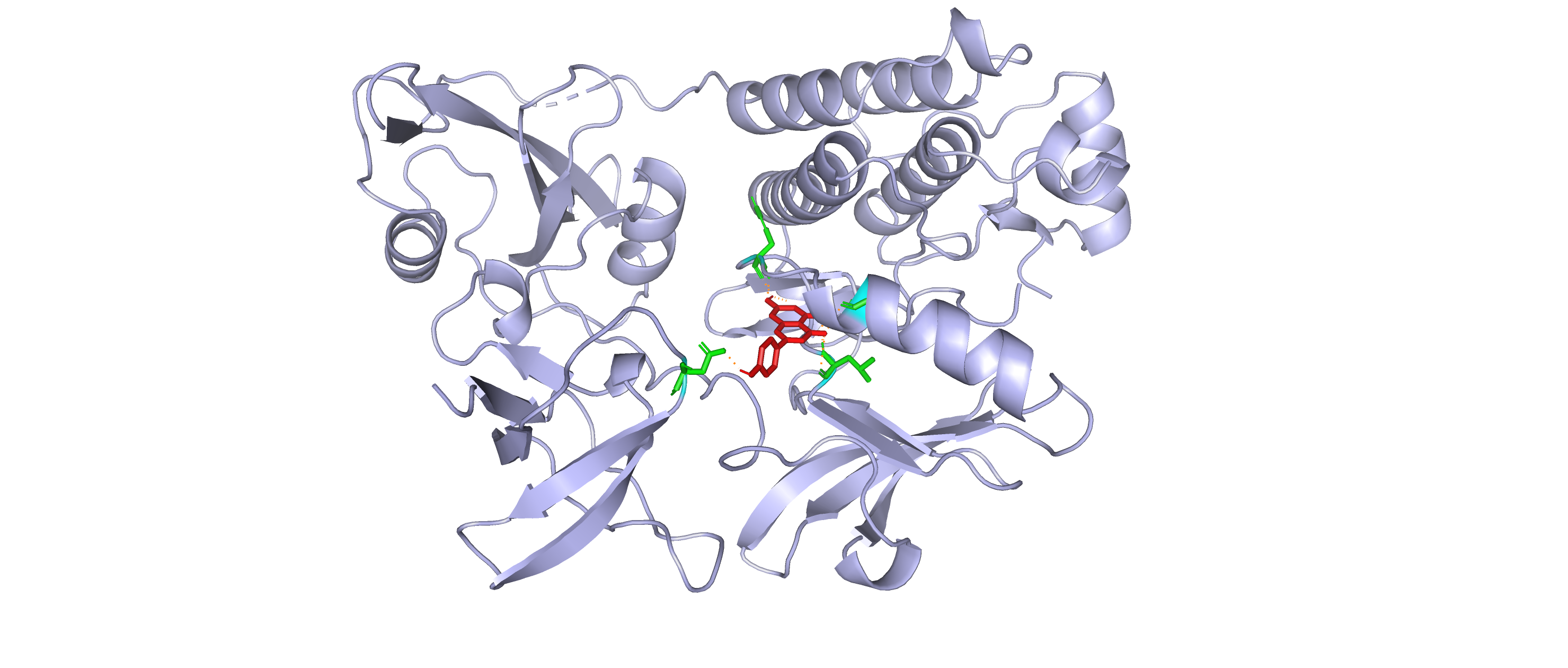

Supplement: Supplementary file 1 — Supplementary Material 1 [file 12876_2025_4196_MOESM1_ESM.zip › Supplementary Materials/Figure 8/Figure8-SRC_API_A.png]

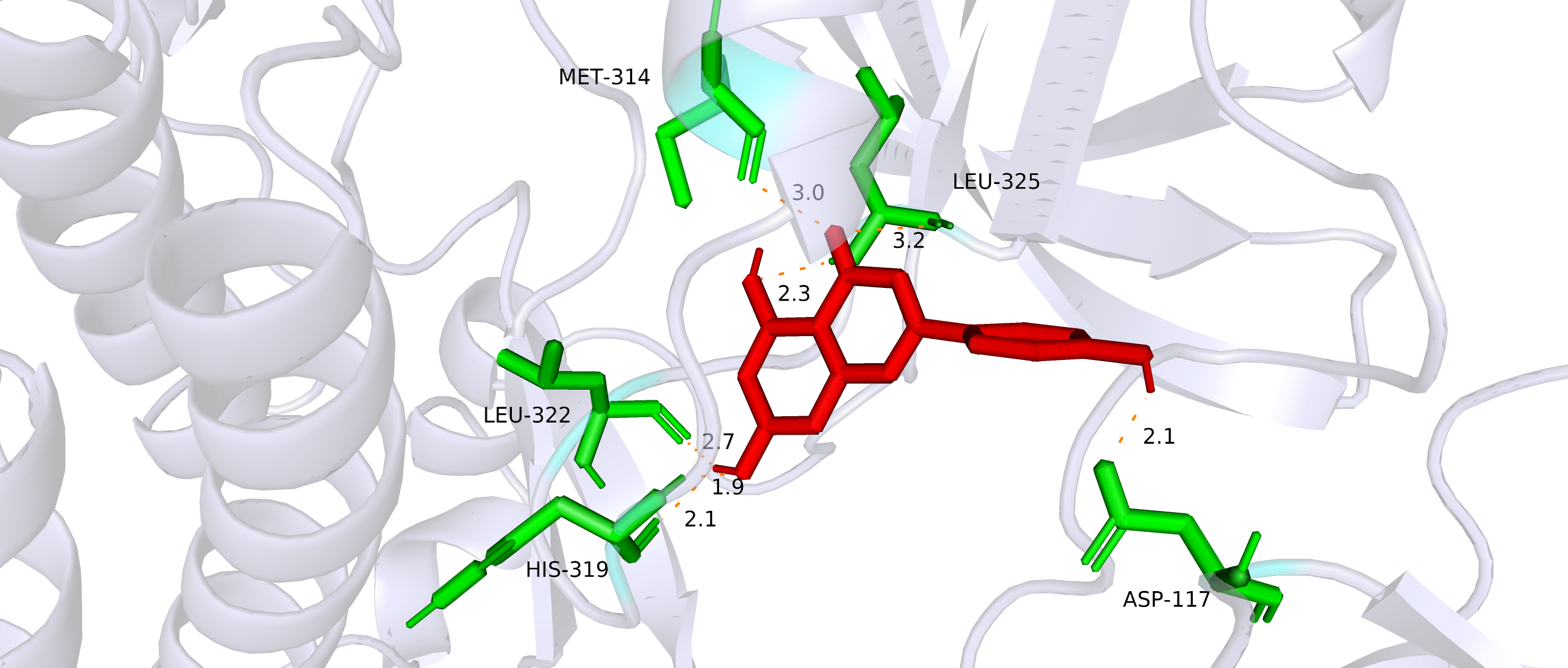

Supplement: Supplementary file 1 — Supplementary Material 1 [file 12876_2025_4196_MOESM1_ESM.zip › Supplementary Materials/Figure 8/Figure8-SRC_API_B.png]

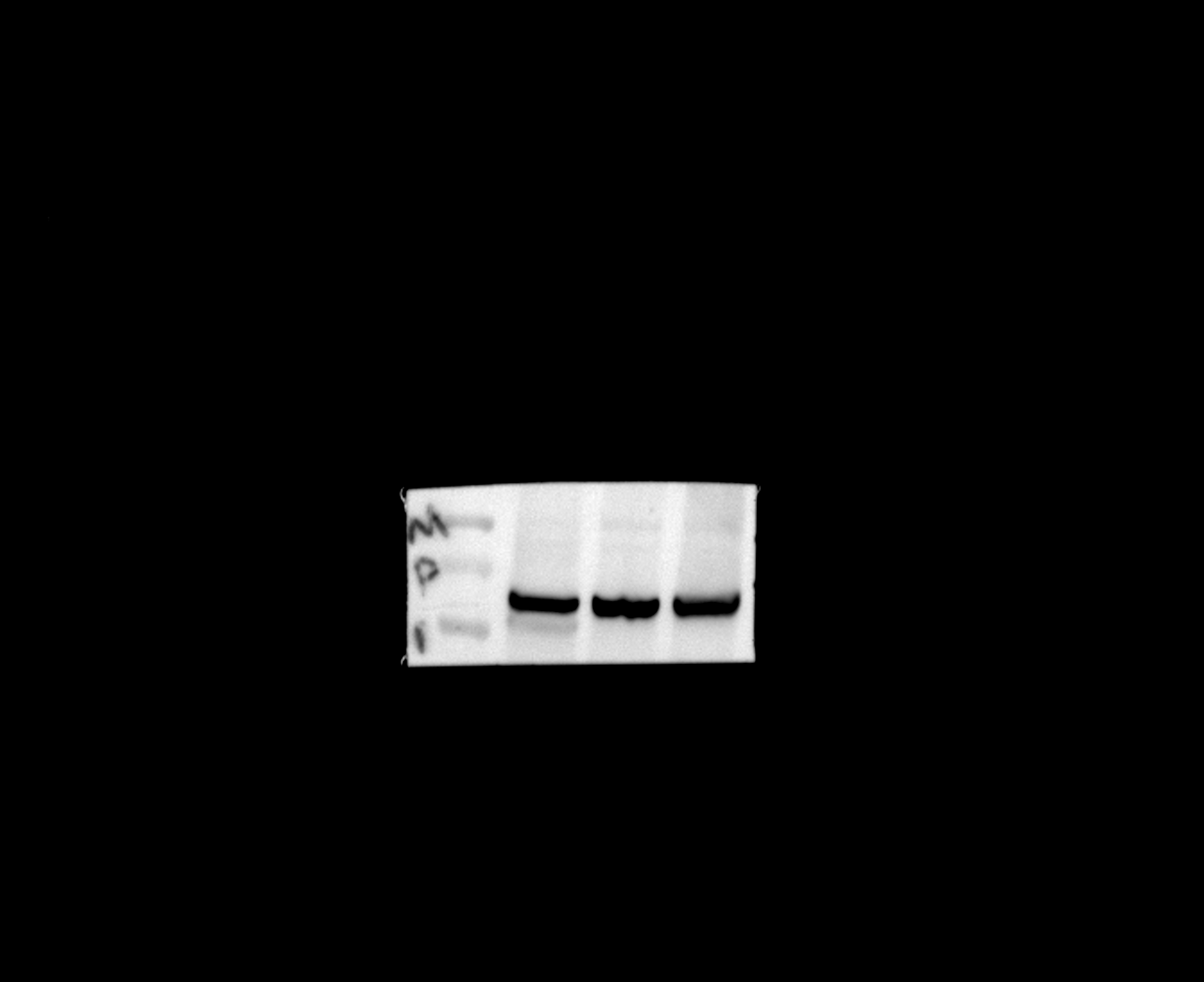

Supplement: Supplementary file 1 — Supplementary Material 1 [file 12876_2025_4196_MOESM1_ESM.zip › Supplementary Materials/Figure 9/Figure9-AKT.Tif]

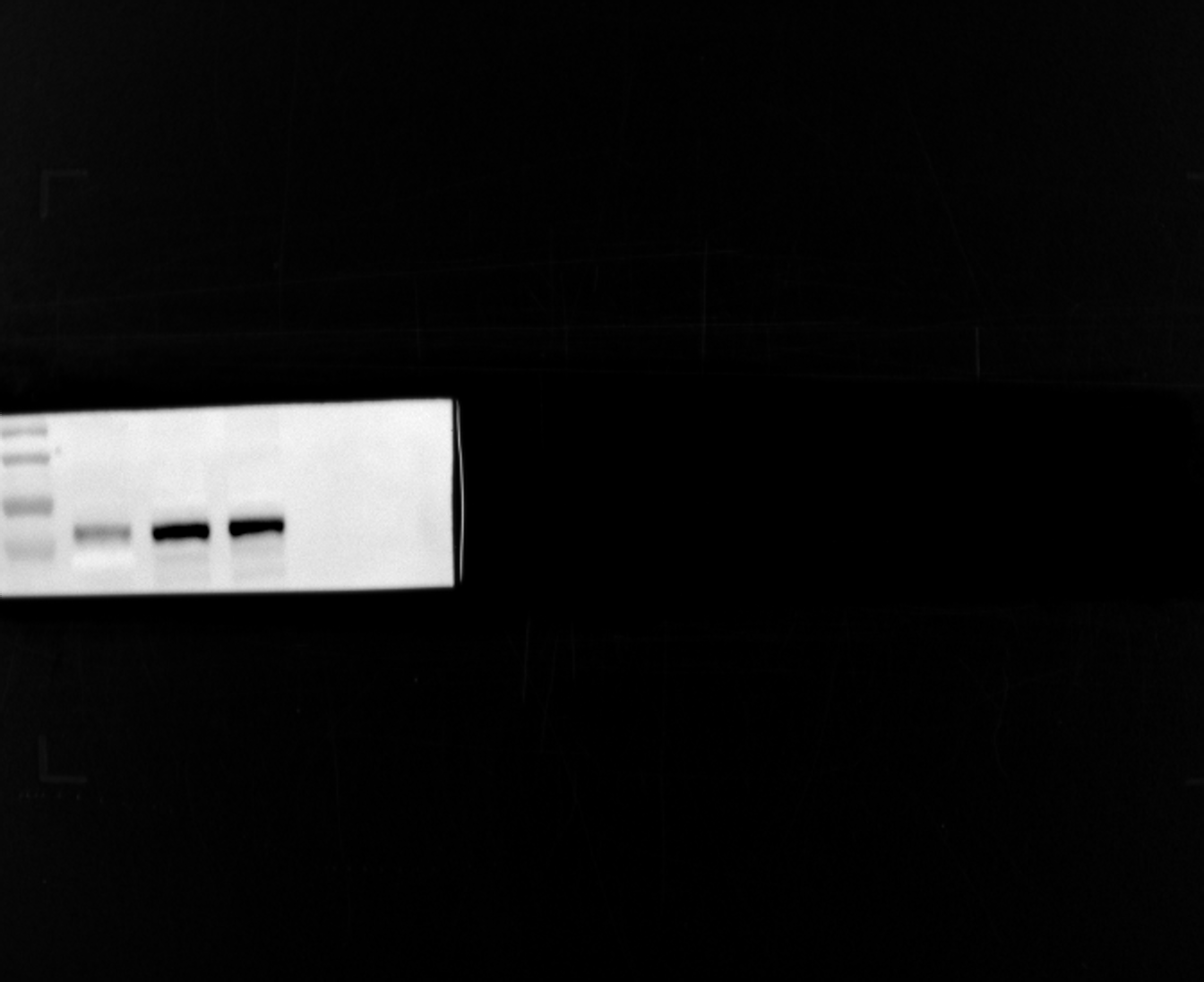

Supplement: Supplementary file 1 — Supplementary Material 1 [file 12876_2025_4196_MOESM1_ESM.zip › Supplementary Materials/Figure 9/Figure9-COX2.Tif]

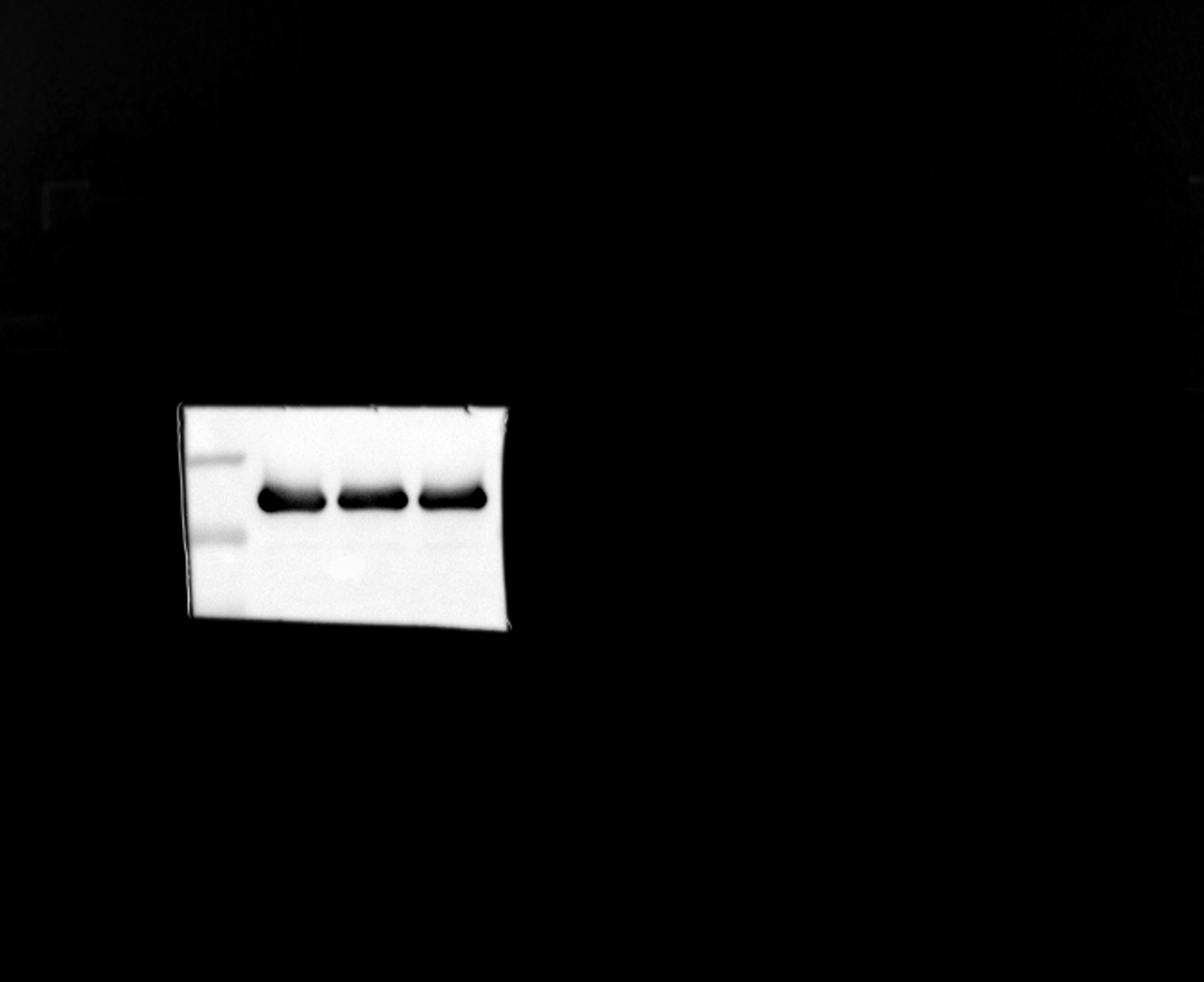

Supplement: Supplementary file 1 — Supplementary Material 1 [file 12876_2025_4196_MOESM1_ESM.zip › Supplementary Materials/Figure 9/Figure9-GAPDH.Tif]

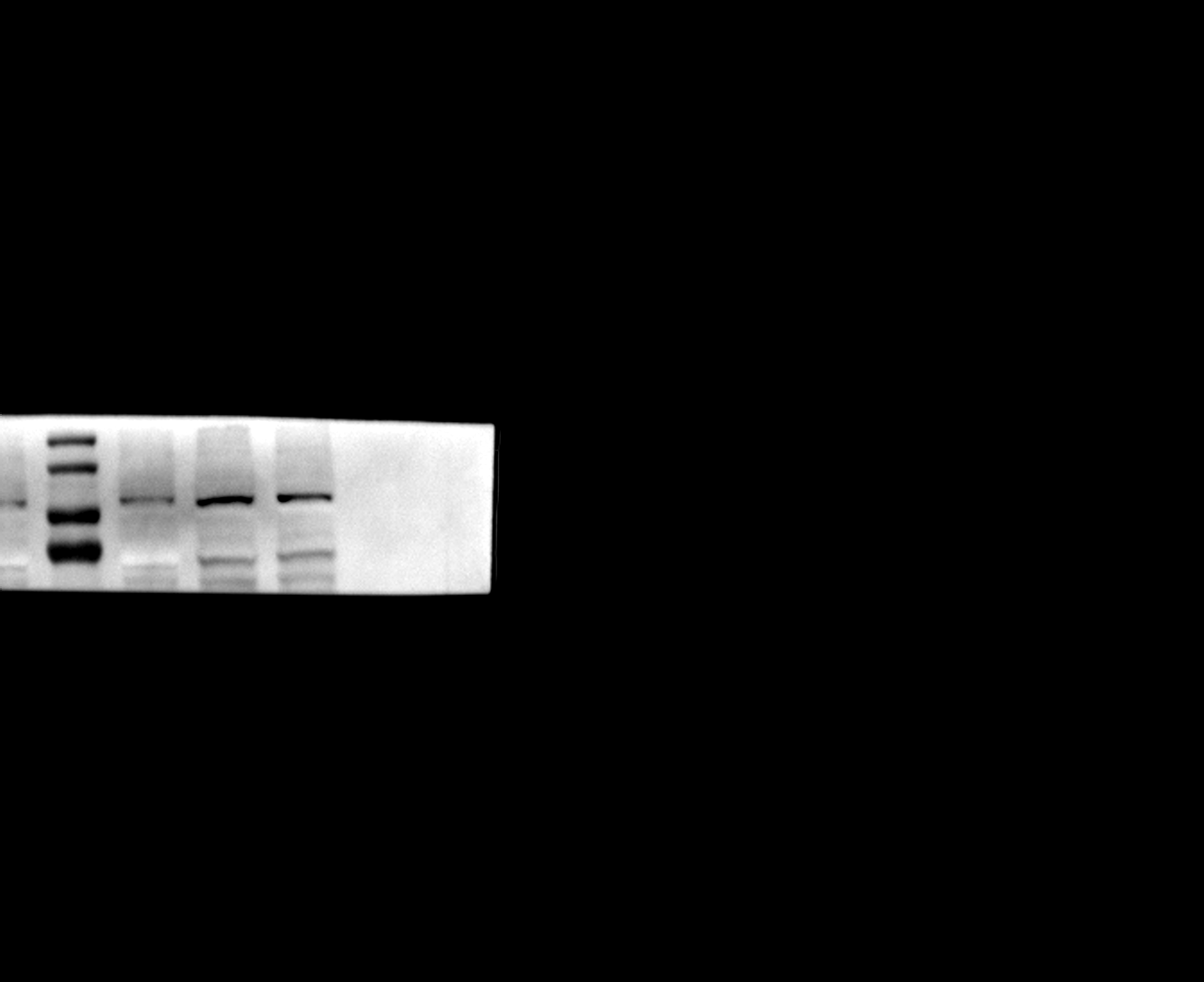

Supplement: Supplementary file 1 — Supplementary Material 1 [file 12876_2025_4196_MOESM1_ESM.zip › Supplementary Materials/Figure 9/Figure9-MMP9.Tif]

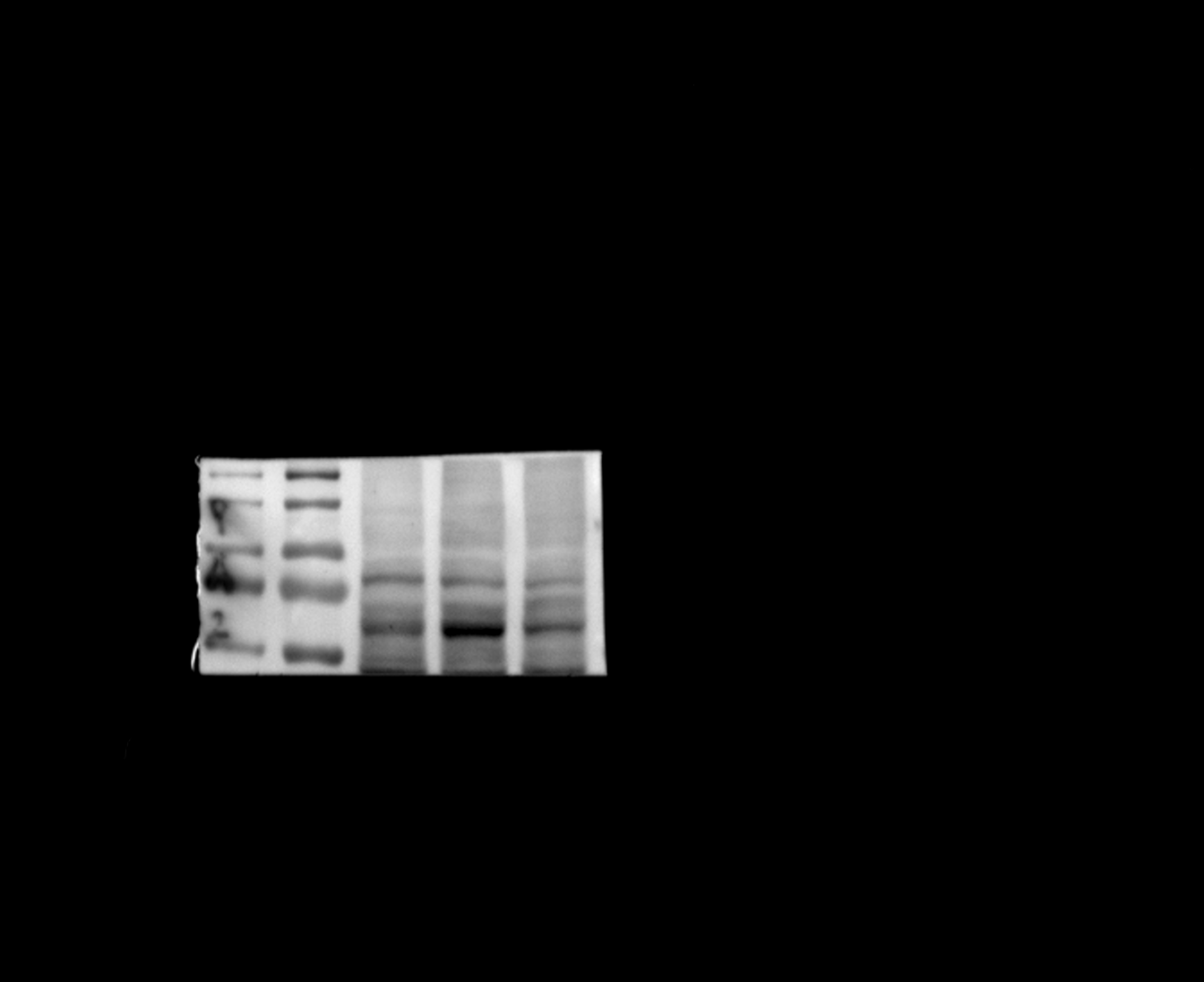

Supplement: Supplementary file 1 — Supplementary Material 1 [file 12876_2025_4196_MOESM1_ESM.zip › Supplementary Materials/Figure 9/Figure9-p-AKT.Tif]

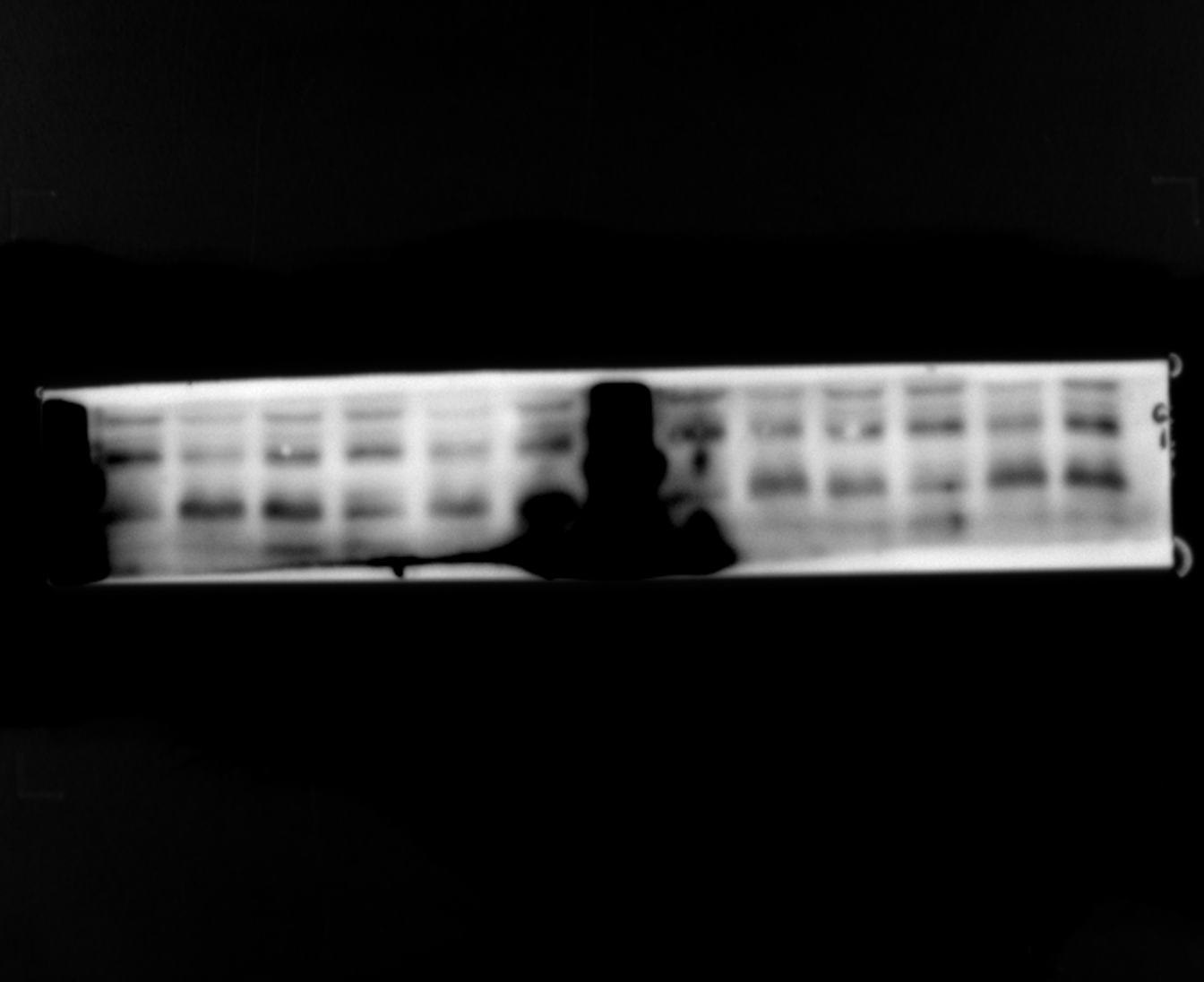

Supplement: Supplementary file 2 — Supplementary Material 2 [file 12876_2025_4196_MOESM2_ESM.zip › wb-2025-5-27/Figure4-G/Figure4-Claudin1.Tif]

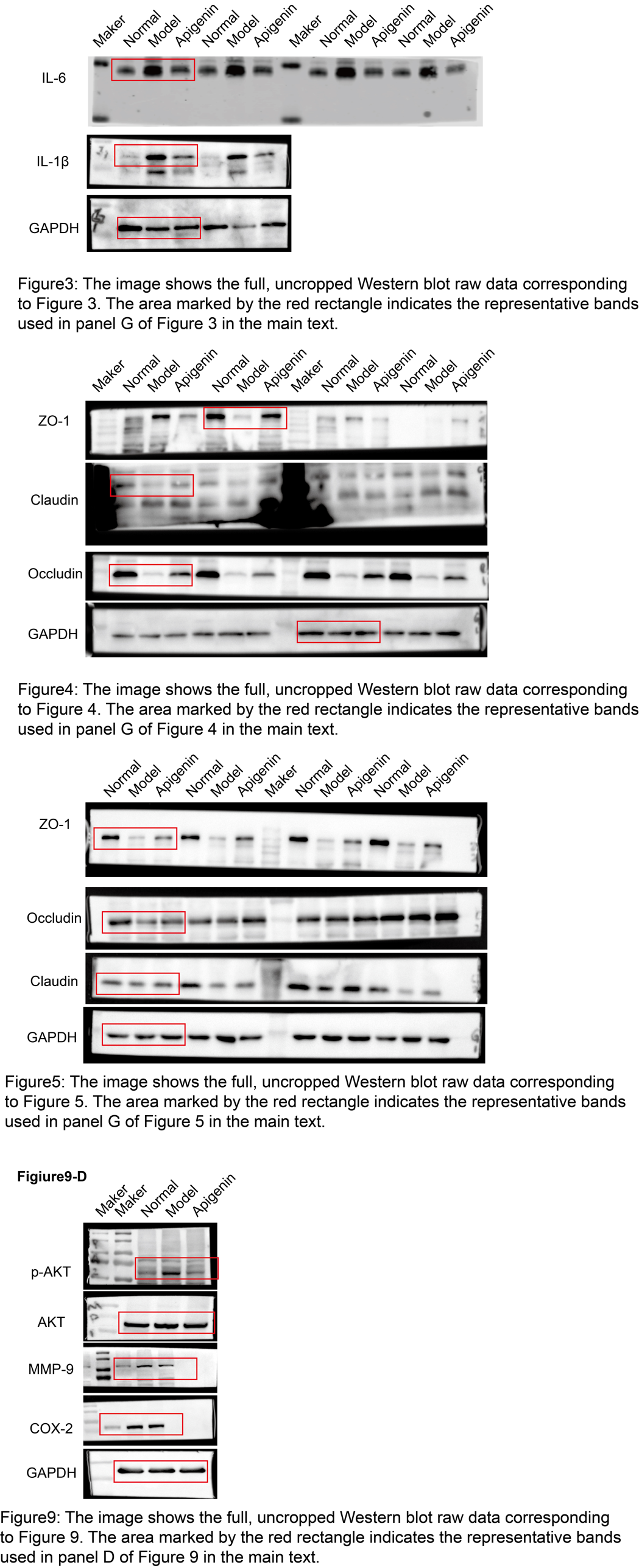

Supplement: Supplementary file 3 — Supplementary Material 3 [file 12876_2025_4196_MOESM3_ESM.jpg]
